# Complete and Efficient Covariants for Three-Dimensional Point Configurations with Application to Learning Molecular Quantum Properties

**DOI:** 10.1021/acs.jpclett.4c02376

**Published:** 2024-12-13

**Authors:** Hartmut Maennel, Oliver T. Unke, Klaus-Robert Müller

**Affiliations:** † Google DeepMind Zürich, Brandschenkestraße 110, 8002 Zürich, Switzerland; ‡ Google DeepMind Berlin, Tucholskystraße 2, 10117 Berlin, Germany; ¶ Google DeepMind, https://deepmind.google/; § TU Berlin, Machine Learning Group, Marchstraße 23, 10587 Berlin, Germany; ∥ Berlin Institute for the Foundation of Learning and Data, Ernst-Reuter-Platz 7, 10587 Berlin, Germany; ⊥ Max Planck Institute for Informatics Saarbrücken, Saarland Informatics Campus, Building E1 4, 66123 Sarbrücken, Germany; # Department of Artificial Intelligence, Korea University, Seoul 136-713, Korea

## Abstract

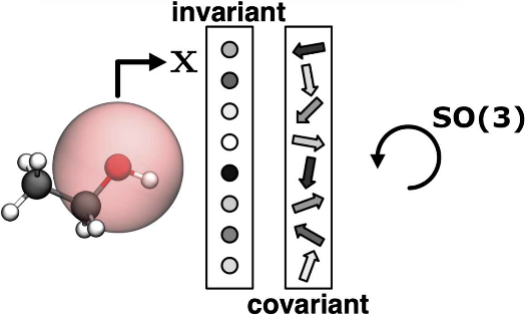

When physical properties of molecules are being modeled with machine learning, it is desirable to incorporate *SO*(3)-covariance. While such models based on low body order features are not complete, we formulate and prove general completeness properties for higher order methods and show that 6*k* – 5 of these features are enough for up to *k* atoms. We also find that the Clebsch–Gordan operations commonly used in these methods can be replaced by matrix multiplications without sacrificing completeness, lowering the scaling from *O*(*l*^6^) to *O*(*l*^3^) in the degree of the features. We apply this to quantum chemistry, but the proposed methods are generally applicable for problems involving three-dimensional point configurations.

Atomistic simulations have proven indispensable for advancing chemistry and materials science, providing insights into the behavior of matter at the atomic level. In the past, these simulations have been computationally demanding, but the advent of Density Functional Theory (DFT)^1^ significantly enhanced the accessibility of atomistic simulations, and recent breakthroughs in machine learning (ML) have further accelerated progress.^2−7^ ML methods trained on *ab initio* data now enable the fast and accurate prediction of quantum properties orders of magnitude faster than traditional calculations.^8−13^ A cornerstone of these methods, whether utilizing kernel-based approaches^14−17^ or deep learning,^18−22^ lies in the effective representation of molecules^23−26^ or materials^27−29^ through carefully chosen features or descriptors. Early examples include the Coulomb Matrix representation^2^ and SOAP,^30^ while recent advancements extend this principle beyond rotationally invariant representations with the design of equivariant model architectures.^31−40^

However, Pozdnyakov et al. pointed out that commonly available descriptors are not able to uniquely identify some molecular structures.^41−43^ This can lead to ambiguities (two distinct structures may be mapped to the same descriptor) that hamper the performance of ML models. Effectively, a lack of uniqueness is similar to introducing a high level of noise into the learning process and may hinder generalization. A second important shortcoming of some modern ML architectures was discussed by Fu et al. and only becomes visible when running molecular dynamics (MD) simulations.^44^ It was observed that ML models with excellent prediction accuracy for energies and forces can nevertheless show unphysical instabilities (e.g., spurious bond dissociation) when simulating longer MD trajectories — limiting their usefulness in practice. Equivariant architectures, however, as broad anecdotal evidence and some theoretical analyses have shown,^44,45^ were found to enable stable MD simulations over long time scales.^16,20,21,36,37,39,44,45^

Both aspects lead to the interesting theoretical question of *how to construct a provably unique invariant, or more generally, a “complete” (to be defined below) equivariant and computationally efficient representation of descriptors for atomistic simulations*. We will study this challenge both by theoretical means and by performing empirical atomistic simulations.

Let us assume that the origin of our coordinate system was fixed meaningfully and we are looking for unique descriptors of point sets that are equivariant under rotations in *SO*(3).

To get invariant features, we can use a rotationally invariant function of *n* points (e.g., distance from the origin for *n* = 1, or angles between two points for *n* = 2), and then sum over all *n*–tuples of points in the configuration. Such descriptors are called “(*n* + 1)-body functions”. It was recently shown that descriptors based on 2- and 3-body information (distances and angles) are unable to distinguish some nonequivalent environments.^46^ Even 4-body information (dihedrals) is not sufficient in all cases (see Figure 1B) and it is necessary to include higher *m*-body information for some structures. Other methods that construct descriptors implicitly, e.g. by message-passing between atoms,^47^ suffer from similar problems^42^ (note that message passing between (*d* – 1)-tuples of points^48^ does not face these issues).

**Figure 1:**
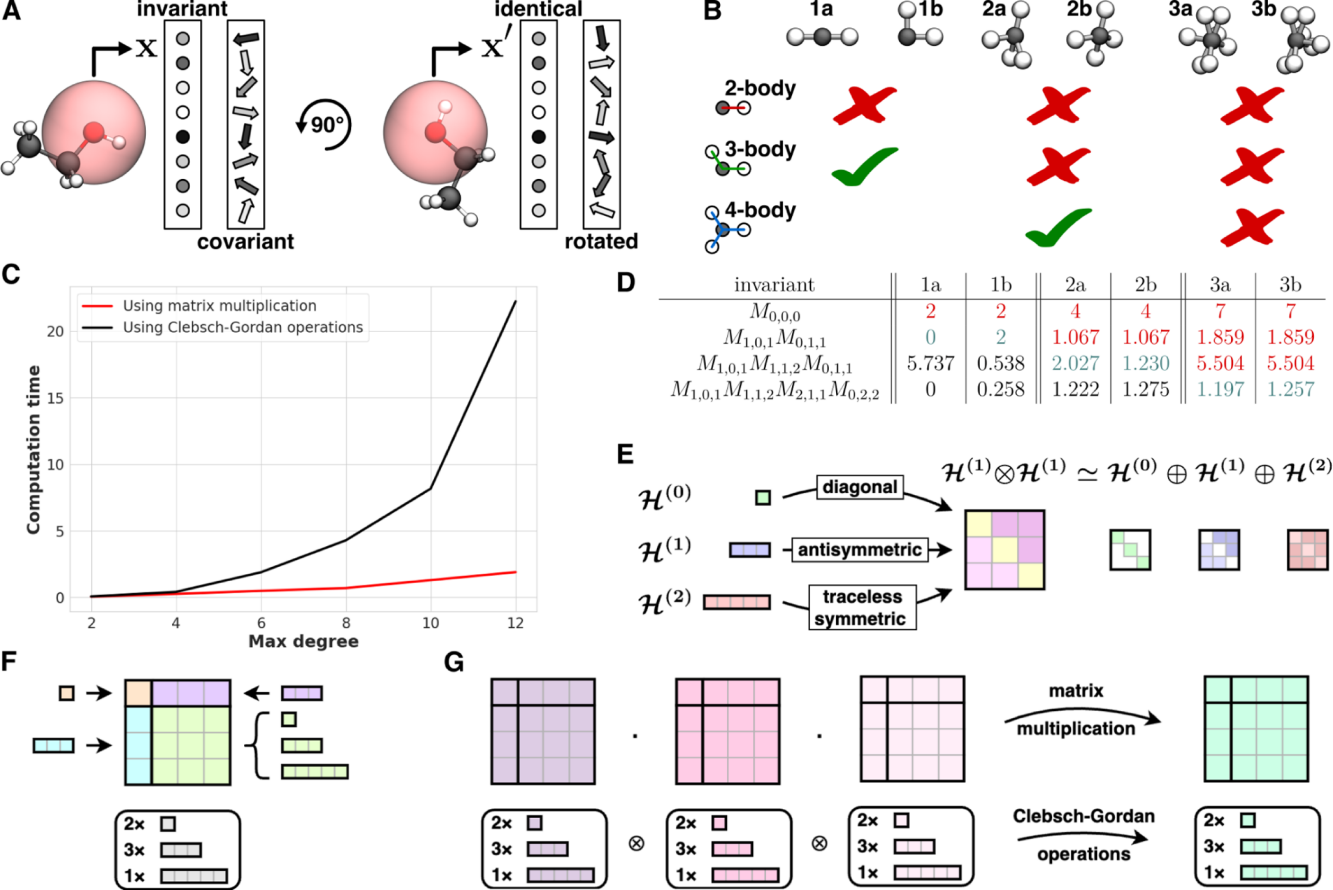
(A) Local chemical environment (translucent red sphere) of an atom (red) described by a fixed-size feature vector that is either invariant or covariant with respect to rotations. (B) Examples for local chemical environments of the black atom (taken from ref (46)) that cannot be distinguished by features constructed from *m*-body information for low *m*. (C) Computational cost for evaluating learned invariants. While Clebsch–Gordan operations scale with *O*(*l*^6^), the proposed implementation replacing them by matrix multiplications scales with *O*(*l*^3^). (D) Completeness theorems say there must be examples of invariants that distinguish the pairs of panel B, given by matrix multiplication as in eq 3. We use *l* = 2; products of *k* matrices give (*k* + 1)-body invariants. (E) We encode lists of features in  as (2*a* + 1) × (2*b* + 1) matrices, visualized here for *a* = *b* = 1. (F) Matrix of (2*a* + 1) × (2*b* + 1) matrices for *a*, *b* ∈ {0, 1} and their irreducible components. (G) Invariants/covariants of higher body order can be constructed from fundamental invariants by either Clebsch–Gordan operations or by matrix multiplication.

Let us start defining an appropriate mathematical language. In applications to chemistry, the points in the point set can belong to different atom types/elements which have to be treated differently. We assume there is a fixed finite set  of “colors” (the atom types/elements), and each point in the point set *S* is assigned a color in , i.e. . We propose to take as potential features all *polynomial point set descriptors* (PPSDs), i.e. all scalar expressions that can be written down for colored point sets, using the coordinates of points, constants from , addition, multiplication, and summations over all points of a given color, such that these expressions can be evaluated for any point set independent of the number of points (see Appendix H1 for formal definitions).

In practice, a variant of PPSDs is more useful, using polynomials only for the angular part (i.e. as a function on the sphere ) and some other function space for the radial part. With the assumptions that these radial functions are analytic and allow approximation of continuous functions in the radius, we can (with some extra effort) prove almost the same theorems, see Appendix B for the definitions, and later sections for details and proofs.

We now describe informally a series of mathematical theorems about PPSDs that we prove in this work, see respective appendices for the precise formulations and proofs.

We first observe (see Appendix H1) that the computation of any scalar PPSD can be arranged into two steps:

1. Evaluate expressions involving only one summation sign: ∑_***r***∈*S*_γ__*P*(***r***) for some color γ and polynomial  acting on point coordinates ***r***. We call them *fundamental features*.
2. Evaluate polynomials in fundamental features.

This separability into two steps allows any PPSD to be **evaluated in time***O*(*n*) where *n* is the number of points (here atoms), which is a major advantage over e.g. descriptors based on rational functions, for which this is generally not possible.

We call a PPSD that can be written such that all polynomials in fundamental features have degree *d* “homogeneous of order *d*”.^a^ The order of such a PPSD is unique, for a proof and a refinement of this notion see Appendix H2. PPSDs of order *d* are also said to be of “**body order***d* + 1” (this convention includes one atom at the origin of the coordinate system in the count).

In this language, there are infinitely many independent *SO*(3)–invariant PPSDs of body order 3, but the examples in ref (46) show that there are inequivalent configurations that cannot be distinguished by invariant functions of body orders ≤4 (see Figure 1B). Our **Topological Completeness Theorem** (Theorem 1 in Appendix D) says that this problem vanishes when we allow arbitrary body orders, even when we restrict the functions to be *polynomial* invariants: Any two *SO*(3)–inequivalent configurations can be distinguished by *SO*(3)–invariant PPSDs, i.e. taking the values of *all polynomial SO(3)–invariant* functions gives a *unique* descriptor. In general for *covariant* functions the values of PPSDs change when we rotate a configuration, so this completeness property has to be expressed differently: We prove that there are enough *SO*(3)–covariant PPSDs to approximate any continuous *SO*(3)–covariant function of colored point sets. (See also ref (49) for functions on configurations of a fixed size.)

Without bound on the number of points in the configurations it is of course necessary to use infinitely many independent invariant functions to distinguish all *SO*(3)–inequivalent configurations, as these form an infinite dimensional space. However, we can ask how many features are necessary to uniquely identify configurations of up to *k* points. Our **Finiteness Theorem** (Theorem 2 in Appendix D) gives a linear upper bound of 6*k* – 5. The proof consists of two parts: The first part asserts that a finite number of features suffice. In the case of polynomial functions, this would follow directly from Hilbert’s (abstract) finiteness theorem, but we also give an explicit bound later in Theorem 4. (For more general radial functions, we use the Noetherian property of the germs of analytic functions as a source of finiteness.) The second part then says that we can orthogonally project down this large space of invariants to dimension 6*k* – 5, with some guarantees for the distance of non–equivalent configurations. Its proof is based on dimensions in real algebraic geometry. A similar statement and proof has been given in ref (50) with a slightly weaker bound (which avoids the construction of the orbifold we get from “dividing by *SO*(3)”). For the case of analytic radial functions we generalize this proof using subanalytic geometry.

The proof shows that, in fact, a randomly chosen projection will work with probability 1; when such a dimensionality reduction is part of a neural network, this means that learning a projection that does not destroy information is very easy, as long as the network can use enough invariants and outputs at least 6*k* – 5 features. (Note that the “probability 1” statement only applies to the coefficients of the linear combination, and hence would only be relevant during training of the network. Once a good invariant mapping has been learned, it will assign unique invariants to *all SO*(3)–orbits of configurations up to *k* points, without exceptions.)

For fundamentally other approaches to characterizing *SO*(3)–orbits of point configurations (of a fixed size and one color), see refs (48, 51−53)

We will now show how to produce unique features in such a way that we never leave the space of covariant features: Let  be the irreducible (2*l* + 1)-dimensional (real) representation of *SO*(3), and  be a *SO*(3)–covariant polynomial (which is unique on the sphere  up to a scalar constant factor, see Appendix G2). These *Y*_*l*_ are given by (real valued) spherical harmonics of degree *l*. We now proceed again in two stages:

1. Evaluate spherical harmonics:^b^ ∑_***r***∈*S*_γ__ |***r***|^2*k*^*Y*_*l*_(***r***) for all colors γ and *k* = 0, 1, 2, ... and *l* = 0, 1, 2, ... These are covariant *fundamental features* (i.e., of order 1) with values in .
2. Iterate for *d* = 1, 2, ...: Compute Clebsch–Gordan operations^c^ for |*l*_1_ – *l*_2_| ≤ *l*_3_ ≤ *l*_1_ + *l*_2_, where the feature in  is a fundamental feature, and the feature in  is of order *d*. This gives covariant features of order *d* + 1.

Clearly, this construction appears to be somewhat special, so we may ask whether it actually gives “enough” invariants (i.e., achieves completeness). This is in fact true in a very strong sense: Our **Algebraic Completeness Theorem** (Theorem 3) says that *all* invariant/covariant PPSDs can be obtained as a linear combination of them; in a sense this is just the isotypical decomposition of the space of all PPSDs (see Appendix I4).

While the above strategy to construct invariant/covariant functions has been used, e.g. in refs (32, 40, 55−59), our novel completeness theorems show that this avoids the potential incompleteness problem pointed out in ref (41). In fact, by our algebraic completeness theorem we get *all* polynomial covariant functions, and by the topological completeness theorem those are *sufficient* to approximate any continuous covariant function. We also get an algebraic completeness theorem for features constructed from tensor products and contractions as in ref (60), see **Theorem 4**; this is based on classical invariant theory.

We now turn to a particular *efficient* variant of our construction. Since invariant PPSDs of order <4 are not sufficient for distinguishing all *SO*(3)–equivalence classes, we need to construct covariants of higher body orders, i.e. in the above procedure we need to use the Clebsch–Gordan products. Note that their computational cost is independent of the number of points, and is linear in the number of products, but scales as *O*(*l*^6^) when we take the tensor product of two representations of the form . But with unrestricted number of points in our configuration, we cannot bound the *l*, even if we are just considering configurations on : Using  only for *l* = 0, 1, ..., *L*–1 yields a –dimensional vector space of fundamental features ∑_***r***∈*S*_γ__*Y*_*l*_(***r***) (and all PPSDs are polynomials in the fundamental features). So this could only describe a configuration space of a dimension , not the ∞–dimensional space of configurations on  with an unbounded number of points.

Consequently, the bottleneck for a larger number of points (necessitating using larger *l* for constructing the fundamental features) can be determined as the *O*(*l*^6^) Clebsch–Gordan operation. We will now propose how to construct local descriptors that alternatively to Clebsch–Gordan operations rely only on matrix–matrix multiplication. This procedure scales as only *O*(*l*^3^) for bilinear operations on two representations of the form . A similar speedup was published recently in ref (61), replacing the Clebsch–Gordan operation by the multiplication of functions. However, since multiplying functions (instead of matrices) is commutative, this does not reproduce the anti–commutative part of the Clebsch–Gordan operations. Therefore, the construction in ref (61) does not have the full expressivity desired and would not satisfy our Algebraic Completeness Theorem or Theorem 5 below. In particular, since commutative products cannot produce pseudo–tensors, its invariants could not distinguish configurations from their mirror images.

Our key idea for removing the computational bottleneck is to apply the Clebsch–Gordan relation
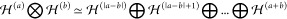
1“backwards” to efficiently encode a collection of features in  as a (2*a*+1) × (2*b*+1) matrix in  (see Appendix G5) and then the matrix multiplication is a covariant map of representations
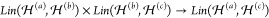
With Schur’s Lemma one can show that it can be expressed as a linear combination of Clebsch–Gordan operations, so unless some coefficients are zero, we can expect this operation to be as useful as the Clebsch–Gordan operations for constructing covariant features of higher body order. This is indeed the case and to formulate the corresponding theorem, we define the involved features: Let  be the embedding given by (1) and define the “matrix moments”
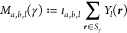
2which are (2*b* + 1) × (2*a* + 1) matrices (see Appendix M for some examples for explicit formulas). Then the result of the multiplication
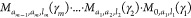
3
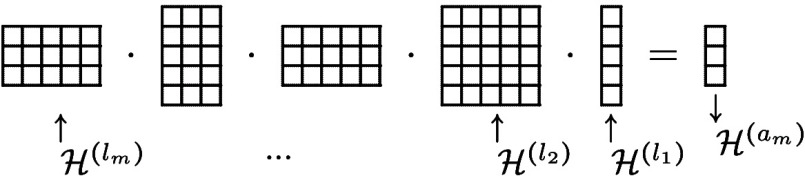
with *l*_1_ = *a*_1_ and |*a*_1_ – *a*_2_ | ≤ *l*_2_ ≤ *a*_1_ + *a*_2_, ..., |*a*_*m*–1_ – *a*_*m*_ | ≤ *l*_*m*_ ≤ *a*_*m*–1_ + *a*_*m*_ are covariant *a*_*m*_ × 1 matrices, i.e. vectors in , given by polynomials of degree *l*_1_ + ... + *l*_*m*_, and computing them takes *O*(*m* · *a*^3^) steps for an upper bound *a* ≥ *a*_*i*_.

Theorem 5 (Algebraic Completeness for features from matrix multiplication). Any *SO*(3)–covariant feature with values in a  can be written as a linear combination of the *SO*(3)–covariants (3) with *a*_*m*_ = *l*. For *O*(3) – covariants it is enough to use those features given by (3) with the appropriate parity of *l*_1_ + ... + *l*_*m*_.

For the proof see Appendix N.

While computing one given invariant of the form (3) would not be more efficient than with Clebsch–Gordan operations (as it would waste whole matrices for encoding only one feature), for applications in Machine Learning we always compute with linear combinations of features (with learnable coefficients), and both the Clebsch–Gordan operation and Matrix Multiplication define maps
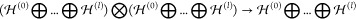
which are used to build up different linear combinations of covariants of higher body order. In the Clebsch–Gordan case we also can add to the learnable coefficients of the input features further learnable parameters that give different weights to the individual parts  that contribute to the same  in the output, whereas in the Matrix Multiplication case these mixture coefficients are fixed (but depend on the shape of the matrices involved). However, Theorem 5 shows that using different shapes of matrices is already sufficient to generate all possible covariants, so both methods can in principle learn the same functions.

For practical applications it is important to organize the matrix multiplications efficiently. In particular when using GPUs/TPUs with hardware support for matrix multiplication, it is much more favorable to compute with a few large matrices than with many small matrices. Therefore, we will use linear combinations of *M*_*a*, *b*, *l*_(γ) for l = |*a* – *b*|, ..., *a* + *b* to fill a (2*b* + 1) × (2*a* + 1) matrix, and pack *r* × *r* small matrices for *a*, *b* in {*l*_1_, *l*_2_, ..., *l*_*r*_} into a large square matrix of side length (2*l*_1_ + 1) + ... + (2*l*_*r*_ + 1), see Figure 2.

**Figure 2:**
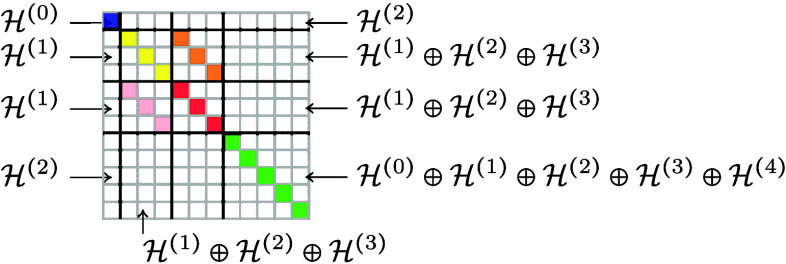
Irreducible components in a matrix of matrices. Traces of square matrices correspond to components , marked here in color.

Then *k*–1 such matrices are multiplied to get a matrix built out of covariants of body order *k*.

This matrix can then be applied to *n*_1_ column vectors from  to get covariant vectors of body order *k* + 1.

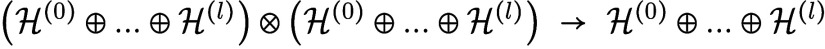



If the end result should be scalars, we can take scalar products of the *n*_1_ column vectors in  with *n*_2_ new covariants in  to obtain *r* · *n*_1_ · *n*_2_ invariants of body order *k* + 2; also the traces of the square submatrices of the matrix product give invariants of body order *k* (marked in color in the above example diagram).

The proposed matrix products approach can be readily used to *replace* Clebsch–Gordan operations across all possible learning architectures giving rise to significant efficiency gains.

As a proof of concept, in the following experiments we will focus on the simplest such architecture which only computes a *linear combination* of many such invariants, see Appendix E for code and more details (e.g., in practice we may want to shift the matrices by the identity to obtain a similar effect to skip connections in ResNets.)

Extensions of this minimal architecture could use a deep neural network instead of a linear combination of invariants, or can use nonlinear activation functions to modify the matrices obtained in intermediate steps. In architectures using several layers of Clebsch–Gordan operations, such activation functions are restricted to functions of the scalar channel, since “you cannot apply a transcendental function to a vector”. Maybe surprisingly, in our matrix formulation this actually becomes possible: Applying any analytic function to our (2*l* + 1) × (2*l* + 1) matrices (not element wise, but e.g. defined by a Taylor series for matrices) is also a covariant operation! Notably, matrix exponentiation has been suggested as an efficient and useful operation in Neural Networks in ref (62).

Our methods yield complete representations and can thus indeed distinguish (molecular) configurations that require higher order features (see refs (42 and 46)). This is demonstrated experimentally in Figure 1B/D.

In Figure 1C we used the library E3x^54^, which allows switching between full tensor layers using the Clebsch–Gordan operation and “Fused Tensor Layers” for which we implemented matrix multiplication instead of the Clebsch–Gordan operations. The plot shows the inference run time measured on CPUs for computing a function defined by two Tensor layers, depending on the setting of “max degree” and whether full or fused layers were used.

In another synthetic experiment, we learn an invariant polynomial of degree 10 with Clebsch–Gordan operations and with our matrix multiplication framework, and plot the training curves averaged over 10 data sets, see Figure 3.

**Figure 3:**
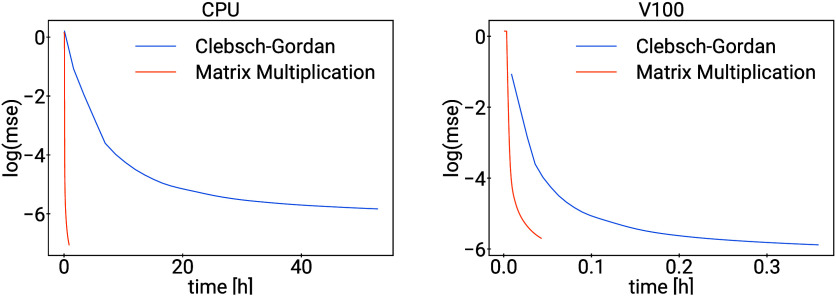
Learning curves for a synthetic toy experiment.

On CPUs, almost all the time is spent in the Clebsch–Gordan operations, and replacing them by the matrix multiplication method makes the training faster by a factor over 100. When using GPUs, the speedup is not quite as dramatic, but still a factor of 8.4 on V100 (details in Appendix P).

As a first demonstration of our framework for atomistic simulations we show that with the simple architecture that *linearly combines* the resulting polynomial invariants, we can learn forces with local features alone that, interestingly, can match the accuracy of other more complex methods which use several message passing/self-attention steps with nonlinear networks (So3krates,^39^) or global kernel methods (sGDML,^16^). Specifically, our experiments (see Table 1) show that accuracies align, notably, independent of the molecule sizes, see Appendix P for details. Since our model is just a linear combination of features of known body order and *L*, in future studies, one could use such models to investigate body order expansions or study the influence of larger *L*s in detail.

**Table 1:** Comparison of Force Accuracies (kilocalories per mole per angstrom) for Our Simple Linear Combination of Polynomial Features with Two More Sophisticated Models

molecule	no. of atoms	no. of samples	sGDML	ours	So3krates
Ac-Ala3-NHMe	42	6000	0.80	0.47	0.24
DHA	53	8000	0.75	0.42	0.24
AT-AT	60	3000	0.69	0.43	0.22
stachyose	85	8000	0.67	0.33	0.44
AT-AT-CG-CG	118	2000	0.70	0.48	0.33
buckyball catcher	148	600	0.68	0.27	0.24
nanotubes	370	800	0.52	0.77	0.73

Appropriately representing chemical structure and atomic environments in molecules and materials is an important prerequisite for accurate machine learning models in chemistry. Ideal descriptors are unique, computationally efficient, and covariant. In this work we have established an algebraic framework that enables a practical construction of provably *complete* system(s) of features with these desired properties that holds for any 3D point configurations. Apart from the abstract theoretical contribution of this work, we show that our construction can be readily implemented as matrix–matrix multiplication – reducing computational complexity from *O*(*l*^6^) to *O*(*l*^3^) compared to Clebsch-Gordan operations. This yields large efficiency gains while maintaining the performance level of standard machine learning models for atomistic simulation.

In summary, our theoretically well founded unique, covariant, and efficient descriptors provide a versatile basis for future atomistic modeling and potentially other applications of machine learning on point configurations.

## Supporting Information Available

Exact mathematical theorems, their context, and their mathematical proofs and details of the experiments (PDF)

## Footnotes

a Note that this is only the degree of the polynomial in fundamental features (step 2), it does not take into account the degrees of the polynomials used to construct the fundamental features themselves. When we multiply out and move all summations to the left (see Appendix H1) this order corresponds to the depth of the summations, since each fundamental feature comes with one summation sign.

b To be precise, we multiply the spherical harmonics with radial basis functions. We use exponent 2*k* here to have only polynomial functions. In practice, we would rather use different, decaying functions, this is treated as “case ii” in general as one of the variations, see Appendix B.

c These project the tensor product of two representations to an irreducible component, see, e.g., ref (54).
