# Supplementary material for: Complete and Efficient Covariants for Three-Dimensional Point Configurations with Application to Learning Molecular Quantum Properties
Source: J Phys Chem Lett. 2024 Dec 13;15(51):12513–9. doi: 10.1021/acs.jpclett.4c02376 (PMC11684023; doi:10.1021/acs.jpclett.4c02376)
Supplement: Supplementary file 1 — jz4c02376_si_001.pdf [file jz4c02376_si_001.pdf]

# Supplement for "Complete and Efficient Covariants for 3D Point Configurations with Application to Learning Molecular Quantum Properties"

Hartmut Maennel,*,† Oliver T. Unke,† and Klaus-Robert Müller*,†,‡,¶,§,‖

*†Google DeepMind*

*‡TU Berlin, Machine Learning Group, Berlin, Germany*

*¶Berlin Institute for the Foundation of Learning and Data, Germany*

*§Max Planck Institute for Informatics Saarbrücken, Germany*

*‖Korea University, Department of Artificial Intelligence, Seoul, Korea*

E-mail: hartmutm@google.com; klausrobert@google.com

# Organization of the appendix

(Headlines in bold are hyperrefs to the corresponding section.)

**A. Overview diagram statements and proofs**

Page 5: We give an overview diagram that explains the main statements and their logical connection. This is meant to accompany the later sections, it is not intended to be fully read and understood on its own.

**B. Variants of $G, X$, and function spaces**

Page 7: Most statements will be true in different settings that we consider, we explain these

settings here. The most important distinction is between the polynomial case (which is used in the main part) and the practically more relevant case of combining polynomial functions of the angular part with more general radial functions; some first relations between these two cases are explained.

**C. Weaker conditions on radial functions**

Page 14: We prove two "finitary" consequences of the two conditions on radial basis functions. In many cases one can directly use these consequences instead of the original conditions to prove statements about configurations with fixed numbers of points.

**D. General theorems**

Page 19: We give exact formulations of the general theorems (Theorems 1 – 4) in the different settings we consider.

**E. Pseudo code for algorithm**

Page 23: We detail the simplest algorithm for parameterized invariants using matrix multiplication and the "matrix of matrices" approach.

**F. Incompleteness of 3 body functions**

Page 29: We illustrate with 2-dimensional examples why 3 body functions are not enough to uniquely characterize point configurations on the circle.

**G. Some background from representation theory**

Page 30: Some specific points from the representation theory of compact groups that are used in the following.

**H. Polynomial point set descriptors (PPSDs)**

Page 40: We define PPSDs, compare them to functions on point configurations of fixed size, and prove the uniqueness of a Normal Form, which is used in the proof of Theorem 4.

**I. Proof of Theorem 1**

Page 55: Topological completeness: Using *all* invariant (/covariant) polynomial point set

descriptors (with values in a given representation of $G$) gives "complete" sets of descriptors. These are mainly topological arguments (embedding into finite dimensional representation, separation of compact orbits, approximation by polynomials on compact subsets). This will be complemented in later sections by algebraic completeness theorems that say that specific constructions span the relevant subspaces of polynomials.

**J. Proof of Theorem 2**

Page 65: Finiteness: Assuming polynomial functions (case i), or analytic radial basis functions and points in a compact region $X$ (case 2ii), we prove that $5n-6$ features are enough to distinguish $G$–equivalence classes of configurations of $n$ points in $\mathbb{R}^3$. We also give counterexamples that this statement fails in case 3ii (no compactness assumption) or when we only require the radial basis functions to be smooth instead of analytic. From weaker assumptions we can at least show that finitely many features are enough.

**K. Proof of Theorem 3**

Page 81: Algebraic completeness for features based on spherical harmonics and Clebsch–Gordan operations. This is essentially the isotypical decomposition of the $G$–representation given by the PPSDs.

**L. Proof of Theorem 4**

Page 83: Algebraic completeness for features based on tensor moments and tensor contractions. This is added for completeness and since the explicit version of the finiteness theorem is most naturally formulated and proven in this context; but otherwise this theorem is not used in the rest of the paper.

**M. Matrix moments examples**

Page 88: The $3 \times 3$ and $5 \times 5$ matrix moments given as concrete matrices of polynomials.

**N. Proof of Theorem 5**

Page 92: Algebraic completeness for features based on spherical harmonics and matrix multiplication.

## O. JAX implementation of matrix multiplication

Page 99: A peculiar run time observation for large numbers of small matrix multiplication on accelerators with hardware support for matrix multiplication. This was a major motivation for the "matrix of matrices" construction.

## P. Details for experiments

Page 100: Description of the numerical experiments on synthetic data and quantum chemistry data.

# A Overview diagram statements and proofs

**Cases:** A,B: $SO(3)$,$O(3)$, 1,2,3: $\mathbb{S}^2$, $r_0 < r < r_1$, $\mathbb{R}^3$, i,ii: Polynomials, radial basis functions.

**Conditions** on radial basis functions in case ii:

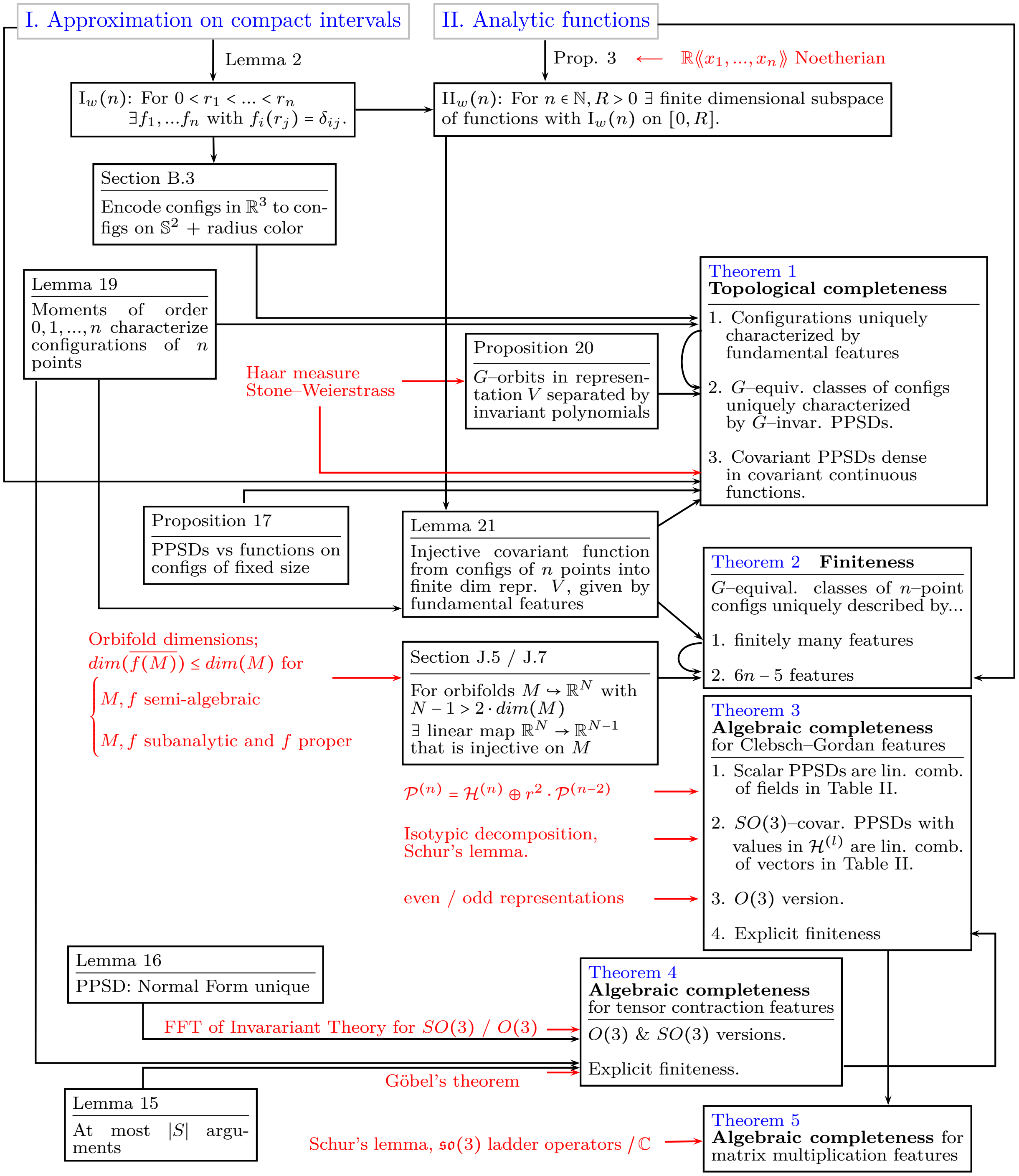


The boxes refer to statements formulated and proven in this appendix, the red text snippets

refer to statements that can be found in the references. Arrows show which statements are used in the proof of another statement. (Crossings of lines have no particular significance, the arrows should be considered as independent.)

**Disclaimer:** This diagram is meant as a slightly simplified overview to accompany the actual definitions, theorems, and proofs. The short formulations should remind the reader which statement is meant, but not give a complete formulation of the prerequisites and detailed statements.

Explanations for some specific parts of the diagram:

- Theorem 1, part 1:

  Lemma 19 is enough in case i (polynomials), but we need the arguments from section B.3 for case ii (radial basis functions).

- Haar measure:

  This means the existence of an invariant measure on our compact group $G = SO(3)$ or $G = O(3)$, it is used to average polynomials over $G$ to get $G$–invariant (or $G$–covariant) polynomials.

- Lemma 21:

  In case i (polynomials) the statement as formulated in the diagram follows from Lemma 19.

  The actual formulation of Lemma 21 in Section I.3 is for case ii (radial basis functions) and assumes the points in the configurations are inside some large ball with radius $R$. This allows applying $\mathrm{II}_w(n)$, but only gives Theorem 2 in cases 1,2 (and it cannot give more, since Theorem 2 is not true in case 3ii).

- Theorem 1, part 2:

  We could use Lemma 21 together with Proposition 20, but that requires the strong

condition II, or its finite version $\mathrm{II}_w(n)$. Instead we use the compactness of the orbits and the weak condition $\mathrm{I}_w(n)$, which is enough for Theorem 1 part 2.

- Proposition 17 / Theorem 1, part 3:

  The full density statement as formulated in Appendix D is requiring density only on compact subsets of the (infinite dimensional) configuration space, Proposition 17 relates this to (finite dimensional) bounded pieces of the configuration space of a fixed number of points of each color.

- Theorem 2: This is only true in cases 1, 2i, 2ii, 3i, we give counterexamples for case 3ii.

- $\mathcal{P}^{(n)} = \mathcal{H}^{(n)} \oplus r^2 \cdot \mathcal{P}^{(n-2)}$:

  $\mathcal{P}^{(n)}$ are the homogeneous polynomials of degree $n$ in $x, y, z$, $\mathcal{H}^{(n)}$ is the subspace of homogeneous polynomials, $r^2 = x^2 + y^2 + z^2$.

- Schur's lemma:

  This is printed in red since it is a well known theorem of representation theory. However, we also include its proof as Lemma 4 since we use it not (as usual) for complex representations, but for odd dimensional real representations.

- FFT:

  First Fundamental Theorem, see Appendix L.

# B Variants of $G, X$, and function spaces

## B.1 Description of variants

We will distinguish the different set ups based on three criteria:

The symmetry group (options A/B), the point domains (options 1/2/3), and the function spaces (i/ii). We will refer to the arising cases by combinations of these tags, e.g. the tag

"2i" will mean any symmetry group (i.e. options A or B), point domain 2, and function class i. Most theorems are valid for any of these cases, with slightly differing proofs. The exception is the finiteness theorem (Theorem 2), which is valid only in the cases 1, 2, and 3i, but not in the case 3ii. We now describe these three criteria in turn.

## Symmetry group $G$

We can distinguish the symmetry groups

A. G=SO(3)

B. G=O(3)

**Usage:**

We would construct $O(3)$ invariants for scalars that are invariant under reflection. If the scalars we want to approximate can distinguish between a point set and its mirror image, then we need $SO(3)$–invariants (or if we know that it is a pseudoscalar, i.e. gets a factor $(-1)$ when reflected, we model it as a $O(3)$–covariant). More generally for covariants, $O(3)$ gives finer information, so if we have the information how the output should change under reflection, we would use $G = O(3)$.

**Representations:**

The group $O(3)$ is the direct product of $SO(3)$ and $\{Id, -Id\}$. Therefore, irreducible representations of $\rho_{l,\sigma} : O(3) \to O(2l+1)$ correspond to irreducible representations $\rho_l : SO(3) \to SO(2l+1)$ with an additional sign $\sigma \in \{\pm 1\}$ such that $\rho(-Id_3) = \sigma \cdot Id_{2l+1}$. For $\sigma = 1$ we call the representation even, for $\sigma = -1$ odd.

## Point domains $X \subseteq \mathbb{R}^3$

We distinguish three cases of subsets of $\mathbb{R}^3$ from which the points are taken:

1. Sphere: $X = \mathbb{S}^2$

2. Spherical shell: $X = \{\boldsymbol{r} \in \mathbb{R}^3 \,\big|\, r_0 \le |\boldsymbol{r}| \le r_1\}$

3. Full space: $X = \mathbb{R}^3$

**Usage:**

In chemistry we may want to model energy contributions from each atom of a molecule. If another atom gets too close to the central atom (that we use as origin of our coordinates), the energy approaches infinity. Since we are usually only interested in conformations below some energy cutoff, this also gives a lower limit $r_0$ of the distances between atoms. On the other hand, we may only use contributions of atoms that are within a certain radius $r_1$ from the central atom, and ignore or model separately longer range interactions. So for this application, a domain of the form 2, i.e.

$$X = \{\boldsymbol{r} \in \mathbb{R}^3 \,\big|\, r_0 \le |\boldsymbol{r}| \le r_1\}$$

seems the most appropriate. The variant 1 ($X = \mathbb{S}^2$) is mainly important for us as an intermediate step to prove theorems about variants 2 and 3, and variant 3 is mainly to state general theorems without specifying $r_0, r_1$.

**Function spaces**

When $X$ is not only the sphere, we also allow the function spaces:

i. polynomials, or

ii. linear combinations of the constant 1 and of functions of the form

$$\boldsymbol{r} \;\mapsto\; g_i(|\boldsymbol{r}|) \cdot P\Big(\frac{\boldsymbol{r}}{|\boldsymbol{r}|}\Big)$$

with a polynomial $P$ and $g_i$ one of a set of continuous radial basis functions $g_i : \mathbb{R}_{\ge 0} \to \mathbb{R}$ with $g_i(0) = 0$ for $i$ in some index set $I$.

**Conditions on the radial basis functions:**

We required $g_i(0) = 0$ above because otherwise the product with a non-constant polynomial on $\mathbb{S}^2$ would not be well defined at the origin. Furthermore, we require

I. Any continuous function on a compact interval $[a, b]$ with $0 < a < b$ can be approximated uniformly by linear combinations of the $g_i$.

II. The $g_i$ are analytic functions.

The condition II is only fully needed in Theorem 2 for obtaining the upper bound on the number of features needed for unique characterization. For other statements, weaker conditions are enough, see section C below.

**Usage:**

For chemistry applications the functions on $X = \{\boldsymbol{r} \in \mathbb{R}^3 \big| r_0 \le |\boldsymbol{r}| \le r_1\}$ that model the influence of atoms around a central atom will most likely decay towards infinity, in fact, we would use functions that are 0 for $r \ge r_1$ to limit the number of atoms we have to process per central atom. Similarly, need a better resolution for small $r$ than for large $r$ will be needed. So while with polynomials in $r^2$ we can in principle approximate any continuous function on $[r_0, r_1]$, it may be more efficient to use radial functions that are tailored to the problem at hand. Since $G$ does not mix points of different radii, using these functions instead of polynomials of $r^2$ as the radial part does not change the theory significantly.

**Fundamental features:**

In case ii, we write $\mathcal{R}$ for the vector space of allowed radial functions, so for 2ii these are the $h : [r_0, r_1] \to \mathbb{R}$ that are linear combinations of the $g_i$, and for 3ii these are the $h : \mathbb{R}_{\ge 0} \to \mathbb{R}$ that are the linear combinations of the $g_i$ (so in particular, we have $h(0) = 0$ for all $h \in \mathcal{R}$).

In the case i any PPSD can be given as a polynomial in fundamental features

$$\sum_{\boldsymbol{r} \in S_\gamma} P(\boldsymbol{r})$$

for a polynomial $P : \mathbb{R}^3 \to \mathbb{R}$ (see section H.1).

In the case ii we consider the features

$$\sum_{\boldsymbol{r} \in S_\gamma} c + P\Big(\frac{\boldsymbol{r}}{|\boldsymbol{r}|}\Big) \cdot h(|\boldsymbol{r}|)$$

for $c \in \mathbb{R}$, $P : \mathbb{S}^2 \to \mathbb{R}$ a polynomial (considered as a function on $\mathbb{S}^2$) and $h \in \mathcal{R}$ as the fundamental features. (The additive constant $c$ is necessary since in case 3ii $h(0) = 0$; this ensures that — like in the case i — the $|S_\gamma|$ are also fundamental features.) We then define the PPSDs to be the polynomials in the fundamental features. (This is a slight abuse of the acronym PPSD, since as functions on configurations with $k$ points, i.e. on $\mathbb{R}^{3k}$ these will no longer be polynomials in general.)

To keep arguments in cases i and ii similar, we define in case i the vector space $\mathcal{R}$ to be the functions $f(r)$ that can be given by a polynomial $P$ as $f(r) = P(r^2)$ and satisfy $f(0) = 0$. Then the fundamental features that *only depend on the radius* are

$$\sum_{\boldsymbol{r} \in S_\gamma} f(|\boldsymbol{r}|) \qquad \text{for } f \in \mathbb{R} + \mathcal{R}.$$

in both cases i and ii.

In case i the

$$\sum_{\boldsymbol{r} \in S_\gamma} P(\boldsymbol{r}) f(|\boldsymbol{r}|) \qquad \text{for } f \in \mathcal{R},\ P \text{ polynomial}$$

are also fundamental features, and for case ii

$$\sum_{\boldsymbol{r} \in S_\gamma} P\Big(\frac{\boldsymbol{r}}{|\boldsymbol{r}|}\Big) f(|\boldsymbol{r}|) \qquad \text{for } f \in \mathcal{R},\ P \text{ polynomial}$$

are also fundamental features.

## B.2 Separation properties of radial functions

We here point out a property of the radial functions $\mathcal{R}$ that will be used in different proofs and follows from Condition I alone.

Its proof is based on the following well known property of polynomials:

**Lemma 1.** *Let $x_0, x_1, ..., x_n$ be $n+1$ different real numbers, then there is a polynomial $P(x)$ of degree $n$ with $P(x_0) = 1$ and $P(x_i) = 0$ for $i = 1, 2, ..., n$.*

*More generally, for any values $v_0, ..., v_n \in \mathbb{R}$ there is a polynomial $Q(x)$ of degree $\leq n$ with $Q(x_i) = v_i$.*

*Proof.* For the first part, set

$$P(x) := \frac{(x - x_1) \cdot (x - x_2) \cdot ... \cdot (x - x_n)}{(x_0 - x_1) \cdot (x_0 - x_2) \cdot ... \cdot (x_0 - x_n)}$$

For the general case, build for all $i = 0, 1, ..., n$ in the same way polynomials $P_i(x)$ for which $P_i(x_j) = \delta_{ij}$ for all $j = 0, 1, ..., n$, and then set

$$Q(x) := v_0 \cdot P_0(x) + ... + v_n \cdot P_n(x).$$

□

For radial functions we get from this:

**Lemma 2** (Separation of radial functions)**.**

*Assume only Condition I. Then for any list of radii $0 \leq t_0 < t_1 < ... < t_n$ there exist functions $f_i \in \mathbb{R} + \mathcal{R}$ such that*

$$f_i(t_i) = 1 \quad \textit{and} \qquad f_i(t_j) = 0 \quad \textit{for } j \neq i. \tag{1}$$

*Proof.* **Case i: Polynomials**

With Lemma 1 we can find a polynomial $P_i(t)$ such that $P_i(t_j^2) = \delta_{ij}$ for $i = 0, 1, 2, ..., n$ and this gives the $f_i \in \mathbb{R} + \mathcal{R}$ of the form $f_i(r) := P_i(r^2)$ with (1).

**Case ii: General radial basis functions**

Let $f : \mathbb{R} + \mathcal{R} \to \mathbb{R}^{n+1}$ be the linear map that evaluates a function $g \in \mathbb{R} + \mathcal{R}$ at the points $t_0, t_1, ..., t_n$. We claim that this map is surjective: Let $v := (v_0, ..., v_n)$ be a given $(n+1)$–tuple, then construct a polynomial $p$ with $p(r_i) = v_i$. By assumption on the basis functions $g_i$ there must be a sequence of functions in $\mathbb{R} + \mathcal{R}$ that approximates $p$, and hence $\boldsymbol{v}$ must lie in the closure of $f(\mathbb{R} + \mathcal{R})$. Since this image $f(\mathbb{R} + \mathcal{R})$ is a vector subspace of $\mathbb{R}^{n+1}$, it is closed, and hence $\boldsymbol{v}$ must already lie in $f(\mathbb{R} + \mathcal{R})$. Now this surjectivity shows that also in the case ii we have functions $f_0, ..., f_n \in \mathbb{R} + \mathcal{R}$ with (1) as in the case i. □

## B.3 Capabilities to distinguish configurations

As an illustration how the different cases of function spaces and point domains are related to each other, we here prove that they have the "same capabilities to distinguish between configurations" — this will be used in the first part of the topological completeness theorem: Any two different configurations of colored points can be distinguished by fundamental features.

Obviously, the claim for 2i, 2ii, 3i, 3ii implies the claim for case 1, so we only have to prove the other direction.

Assume we know this is true in case 1, i.e. on $X = \mathbb{S}^2$, and we have a pair of configurations in $\mathbb{R}^3$ with colors in $\mathcal{C}$ that we want to distinguish by fundamental features. Let $t_0 = 0$ and $0 < t_1 < ... < t_n$ be the positive radii appearing among any points in one of the two configurations.

We first note that by Lemma 2 radial functions can pick out any particular radius. We will use the functions of Lemma 2 to show that encoding configurations on shells of $n$ radii $r_1, ..., r_n$ is equivalent to encoding configurations on $\mathbb{S}^2$ with $n$ times more colors:

First, we can discard a potential point at the origin: From the value of the fundamental feature $\sum_{\boldsymbol{r} \in S_\gamma} f_0(|\boldsymbol{r}|^2)$ we already see whether the origin is in a configuration and which color

it has. So it is enough to consider the points outside of the origin.

We can now "reduce" the two configurations in $\mathbb{R}^3$ colored by $\mathcal{C}$ to two configurations on $\mathbb{S}^2$ colored by $\mathcal{C}' := \mathcal{C} \times \{1, 2, ..., n\}$: Map any point $\boldsymbol{r} \in \mathbb{R}^3$ with color $c \in \mathcal{C}$ and radius $|\boldsymbol{r}| = t_i$ to $\boldsymbol{r}/|\boldsymbol{r}| \in \mathbb{S}^2$ with color $(c, i) \in \mathcal{C}'$. On the other hand, given one of the derived configurations on $\mathbb{S}^2 \times \mathcal{C}'$, we can reconstruct the original configuration on $\mathbb{R}^3 \times \mathcal{C}$, and the original configurations are $G$–equivalent if and only if the reduced configurations are $G$–equivalent.

Since we assume we know the statement for $X = \mathbb{S}^2$, there is a fundamental feature $\sum_{\boldsymbol{r}' \in S'_{(\gamma,j)}} P(\boldsymbol{r}')$ with $(\gamma, j) \in \mathcal{C}'$ that is able to distinguish them. By decomposing $P$ into its homogeneous components, we can assume $P$ is a homogeneous polynomial of degree $d$. But then we can re-write the fundamental feature on $X = \mathbb{S}^2$ as a fundamental feature in the original setup using the $f_i \in \mathbb{R} + \mathcal{R}$ from Lemma 2:

$$\sum_{\boldsymbol{r}' \in S'_{(\gamma,j)}} P(\boldsymbol{r}') = \sum_{\boldsymbol{r} \in S_\gamma} f_j(|\boldsymbol{r}|) P\Big(\frac{\boldsymbol{r}}{|\boldsymbol{r}|}\Big) = \sum_{\boldsymbol{r} \in S_\gamma} f_j(|\boldsymbol{r}|) \cdot t_j^{-d} \cdot P(\boldsymbol{r})$$

(we use the second expression in case ii and the third in case i). So then this feature in the original setup of cases 2 and 3 must also be able to distinguish the two configurations, q.e.d.

# C Weaker conditions on radial functions

In case ii we required Conditions I, II from the radial basis functions $g_i$. These conditions are enough to prove all theorems we are going to prove, and are partly selected for their simple formulation. However, for most theorems they are more restrictive and idealized than they need to be; in particular Condition I can only be satisfied for an infinite collection of $g_i$.

Here we formulate two consequences of Conditions I, II that are used in some proofs, and that are enough on their own for some statements. These are weaker conditions, and they are parameterized by a natural number $n$ and can also be applied to finite sets of $g_i$ and give results for point sets up to a given size.

- Condition $\mathrm{I}_w(n)$:

  For any $n$ different real numbers $t_i > 0$ there is a linear combination $f$ of the $g_i$ such that $f(t_1) = 1$ and $f(t_i) = 0$ for $i > 1$.

- Condition $\mathrm{II}_w(n)$: For any $R > 0$ there is a *finite* subset $J$ of indices such that for any $n$ different numbers $0 < t_i < R$ there is a linear combination $f$ of the $g_j$ with $j \in J$ such that $f(t_1) = 1$ and $f(t_i) = 0$ for $i = 1, 2, ..., n$.

As an example, the $g_i(t) := t^i$ for $i = 1, 2, ...n$ satisfy Conditions $\mathrm{I}_w(n)$ and $\mathrm{II}_w(n)$.

Obviously, Condition $\mathrm{II}_w(n)$ is stronger than Condition $\mathrm{I}_w(n)$. Lemma 2 above showed that $I \Rightarrow I_w(n)$ for all $n$. The main result of this section will be that Condition $\mathrm{II}_w(n)$ follows from Condition $\mathrm{I}_w(n)$ + Condition II (Proposition 3 below).

We can also include $t = 0$ in these conditions if we allow also the constant 1 in the linear combinations for $f$:

- Condition $I'_w(n)$:

  For any $n$ different real numbers $t_i \geq 0$ there is a linear combination $f$ of 1 and the $g_i$ such that $f(t_1) = 1$ and $f(t_i) = 0$ for $i > 1$.

- Condition $II'_w(n)$: For any $R > 0$ there is a *finite* subset $J$ of indices such that for any $n$ different numbers $0 \leq t_i < R$ there is a linear combination $f$ of 1 and the $g_j$ with $j \in J$ such that $f(t_1) = 1$ and $f(t_i) = 0$ for $i = 1, 2, ..., n$.

The primed versions are equivalent to the original conditions: If $t_1 > 0$, the condition $f(0) = 0$ is automatically satisfied since all $g_i$ for $i \in I$ satisfy $g_i(0) = 0$; if on the other hand $t_1 = 0$, then set $f := 1 + \sum_{j=1}^{m} \alpha_j g_{i_j}$ and solve for $\sum_{j=1}^{m} \alpha_j g_{i_j}(t_k) = -1$ for $k = 2, 3, ..., n$ (we can find such a linear combination with the same argument as we did in the second part of Lemma 1.)

Note that the formulation of Condition $\mathrm{II}_w(n)$ involved an upper bound $R$, and the reason is that without this finite upper bound $R$ it does *not* follow from Conditions I and II:

**Counterexample** for $\mathrm{II}_w(n)$ without finite $R$:

Assume the radial basis functions are (the constant 1 and) $\sin\left(\frac{2a-1}{2^b}\cdot r\right)$, and $\cos\left(\frac{2a-1}{2^b}\cdot r\right)-1$ for natural numbers $a,b=1,2,\ldots$. Linear combinations of these functions are enough to approximate any continuous function in any compact interval of $r$, but for finitely many of them there will be an upper bound $b<B$ and then these functions cannot distinguish distance $r$ from $r+2^B\pi$, so even Condition $\mathrm{II}_w(2)$ would not hold without finite $R$.

We also cannot relax the Condition II from analytic to smooth:

**Counterexample** for $\mathrm{II}_w(n)$ with smooth functions:

Let $\{g_i\,|\,i\in I\}$ be all smooth functions that are of the form $f(t)=c+d\cdot(t-1)^2$ in an arbitrarily small neighborhood of $t=1$. Let $K$ be a compact interval $[a,b]$ with $a\leq 1\leq b$. Then any continuous function on $K$ can be uniformly approximated by using a smooth partition of one and interpolating between the original function on $K\smallsetminus[1-\epsilon,1+\epsilon]$, and a function of the form $c+d\cdot(r-1)^2$ on $[1-\epsilon,1+\epsilon]$ and letting $\epsilon$ go to 0. So these functions would satisfy the strong condition $I$.

However, given only finitely many basis functions $g_i$, there would be a neighborhood of 1 in which every linear combination of these $g_i$ is symmetric around 1, and these functions could not distinguish between $r=1-\epsilon$ and $r=1+\epsilon$ for $\epsilon$ small enough, so these function would not satisfy Condition $\mathrm{II}_w(n)$ for $n=2$.

This counterexample shows the importance of the diagonal $\Delta:=\{(t,t)\in K\times K\,|\,t\in K\}$: While for each compact subset of $K\times K\smallsetminus\Delta$ we can find finitely many functions that distinguish between $r\neq r'$ when $(r,r')$ is in the compact subset, we cannot achieve this for all of $K\times K\smallsetminus\Delta$ because this is not a compact set. So the proof in the analytic case will use local conditions that also include the diagonal.

**Proposition 3.**

*Conditions $I_w(n)$ + II imply Condition $II_w(n)$.*

*Proof.* For given $R>0$ let $K$ be the compact interval $[0,R]$.

Since we need to include 0 in $K$ to use compactness, we will use the version $I'_w(n)$. We will continue to use $\mathcal{R}$ for the vectors space generated by the $g_i$, and consider them as functions on $[0,R]$. We add a new symbol $*$ to $I$ and set $g_*(t) := 1$, then the $g_i$ with $i \in I^* := I \cup \{*\}$ generate $\mathbb{R} + \mathcal{R}$.

We define $\Delta_n$ as the subset of $(t_1, t_2, ..., t_n) \in K^n$ for which two (or more) of the numbers $t_1, t_2, ..., t_n$ are equal, and for $i_1, ..., i_n \in I^*$ we define the functions

$$f_{i_1,...,i_n}(t_1,t_2,...,t_n) := \begin{vmatrix} g_{i_1}(t_1) & g_{i_1}(t_2) & \dots & g_{i_1}(t_n) \\ g_{i_2}(t_1) & g_{i_2}(t_2) & \dots & g_{i_2}(t_n) \\ \vdots & \vdots & \ddots & \vdots \\ g_{i_n}(t_1) & g_{i_n}(t_2) & \dots & g_{i_n}(t_n) \end{vmatrix}$$

and subsets

$$Z_{i_1,...,i_n} := \{(t_1,...,t_n) \in K^n \,\big|\, f_{i_1,...,i_n}(t_1,t_2,...,t_n) = 0\}.$$

If $(t_1, t_2, ..., t_n) \in \Delta_n$, then two (or more) columns in the determinant above are the same, so $f_{i_1,...,i_n}(t_1, t_2, ..., t_n) = 0$. On the other hand, if $t_1, t_2, ..., t_n$ are all different, then Condition $I'_w(n)$ implies we can find functions $h_1, ..., h_n \in \mathbb{R} + \mathcal{R}$ such that $h_j(t_k) = \delta_{j,k}$; this means the $n$ column vectors $g_i(t_1), ..., g_i(t_n)$ for such $i \in I^*$ that occur in one of the $f_j$ cannot be linearly independent, which means there must be $n$ indices $i_1, ..., i_n$ such that the $n \times n$–matrix of the $g_{i_l}(t_k)$ is non-singular. Taken together, this means that

$$\Delta_n = \bigcap_{(i_1,...,i_n)\in(I^*)^n} Z_{i_1,...,i_n}.$$

Furthermore, for $(t_1, t_2, ..., t_n) \notin \Delta_n$, we have $i_1, ..., i_n$ such that $f_{i_1,...,i_n}(t_1, t_2, ..., t_n) \neq 0$ and since all functions are continuous, this is still true in some open neighborhood $U_{t_1,t_2,...,t_n}$ of $(t_1, t_2, ..., t_n)$. This means we have an open neighborhood of $(t_1, t_2, ..., t_n)$ in which

$$\Delta_n \cap U_{t_1,t_2,...,t_n} = \varnothing = Z_{i_1,...,i_n} \cap U_{t_1,t_2,...,t_n}.$$

Now we need to find something similar for $(t_1, t_2, ..., t_n) \in \Delta_n$ (this is the part that would not be possible if we only require smoothness of the $g_i$). If we can find an open neighborhood of $(t_1, t_2, ..., t_n) \in \Delta_n$ such that in this neighborhood $\Delta_n$ is given as intersection of finitely many $Z_{i_1,...,i_n}$, this will allow us to use a compactness argument and get the required finite set $J$. To do this, we will look at a sort of "infinitesimal neighborhood"; the ring of functions in this "infinitesimal neighborhood" is given by germs of analytic functions, i.e. functions that can be expressed locally as a power series in some neighborhood of $(t_1, t_2, ..., t_n)$ with some convergence radius $\epsilon > 0$. This ring of convergent power series is the same (up to isomorphism) at every point $(t_1, t_2, ..., t_n)$, in particular we can identify it with the ring $\mathbb{R}\langle\langle x_1, ..., x_n \rangle\rangle$ of convergent power series in $n$ variables, which is a Noetherian ring, see e.g. Definition 2A and Theorem 102 in.[1] (The ring of germs of smooth functions is not Noetherian.) Let $\mathcal{I}_{t_1,t_2,...,t_n}$ be the ideal in the ring of convergent power series at $(t_1, t_2, ..., t_n)$ generated by all $f_{i_1,...,i_n}$ for $(i_1, ..., i_n) \in (I^*)^n$. Then, since the ring is Noetherian, this ideal can already be generated by a subset $J_{t_1,t_2,...,t_n}$ of finitely many tuples $(j_1, ..., j_n) \in (I^*)^n$. If $\epsilon > 0$ is the smallest convergence radius for the $f_{j_1,...,j_n}$ with $(j_1, ..., j_n) \in J_{t_1,t_2,...,t_n}$, let $U_{t_1,t_2,...,t_n}$ be the open $\epsilon$–neighborhood of $(t_1, t_2, ..., t_n)$. Then this means that

$$\Delta_n \cap U_{t_1,t_2,...,t_n} = \bigcap_{(j_1,...,j_n) \in J_{t_1,t_2,...,t_n}} Z_{j_1,...,j_n} \cap U_{t_1,t_2,...,t_n}.$$

Now we can apply compactness: Since the open subsets $U_{t_1,t_2,...,t_n}$ cover the compact set $K^n$, already finitely many of them do, let $J_n$ be the union of the corresponding finitely many $J_{t_1,t_2,...,t_n}$. That means we have

$$\Delta_n = \bigcap_{(j_1,...,j_n) \in J_n} Z_{j_1,...,j_n}.$$

So for any $n$ different numbers $0 \le t_i < R$ there must be a tuple $(j_1, ..., j_n) \in J_n$ with $f_{j_1,...,j_n} \neq 0$, so we can find a linear combination $f$ of those $g_j$ with $j \in J$ such that $f(t_1) = 1$ and $f(t_i) = 0$ for $i = 1, 2, ..., n$, so $\mathrm{II}_w(n)$ is satisfied. □

# D General theorems

In this section we formulate the general, abstract theorems described informally in the main text.

**Theorem 1** (Topological Completeness). *Let $G$ be either $O(3)$ or $SO(3)$.*

1. *Any two different configurations of points in $X \subseteq \mathbb{R}^3$ colored by $\mathcal{C}$ can be distinguished by their fundamental features.*

2. *Any two configurations of points in $X \subseteq \mathbb{R}^3$ colored by $\mathcal{C}$ that are not* equivalent under *$G$ can be distinguished by $G$–*invariant *PPSDs.*

3. *$G$–*covariant *PPSDs are uniformly dense in $G$–*covariant *continuous functions on compact subsets of configurations colored by $\mathcal{C}$.*

This theorem applies to all cases 1,2i,2ii,3i,3ii. For a proof (and an exact description of the third statement) see Appendix I. In case ii, it only uses Condition I for the first two parts, and additionally Condition $\mathrm{II}_w$ (or the stronger Condition II) for the last part.

These are infinitely many features, and as explained in the main text, without bound on the number of points a finite subset cannot give unique descriptors. For a given bound on the number of points one can show:

**Theorem 2** (Finiteness). *Let $G$ be $O(3)$ or $SO(3)$, and assume we are not in case 3ii.*

1. *Given $k_1, \ldots, k_c \in \mathbb{N}$, there are finitely many $G$–invariant PPSDs that distinguish all $G$–equivalence classes of colored point sets with at most $k_1, \ldots, k_c$ points of colors $1, \ldots, c$.*

2. *Given a finite set of $G$–invariant PPSDs that distinguishes all $G$–equivalence classes of colored point sets with at most $k_1, \ldots, k_c$ points of colors $1, \ldots, c$, and $k := k_1 + \ldots + k_c$, there are $2\dim(X) \cdot k - 5$ linear combinations of them that already distinguish all equivalence classes.*

With $dim(X) = dim(\mathbb{R}^3) = 3$ in case 3 this gives the bound $6k - 5$ stated in the main part. This theorem is true in the cases $1, 2i, 2ii, 3i$, but not in case $3ii$. For the proof we use dimension arguments, depending on the function classes used, this uses dimensions of semi–algebraic sets or of subanalytic sets. In case 2ii it uses Conditions I and II. The first statement that finitely many features suffice for configurations of $k$ points can be proven from only Conditions I and $\mathrm{II}_w(2k)$.

The proof also shows that not only such linear combinations exist, but that $6k - 5$ linear combinations "picked at random" will have this property with probability 1, and that reducing a set of features to this size will reduce distances between configurations by at most a constant factor. See Appendix J for further discussion and proofs.

To describe the algebraic completeness theorem in more detail, we use the schematic depiction of PPSDs in Table 1:

Given a point set in $\mathbb{R}^3$ colored by $c$ colors, the first row contains fundamental features.

Table 1: **Schematic depiction of PPSDs by body order and isotypical component** In the first row, for each $l$ and color a $2l + 1$–dimensional vector, sum of the spherical harmonics $Y_l(\boldsymbol{r})$ over all points $\boldsymbol{r}$ of that color. The $n$–th row for $n > 1$ consists of the results of all Clebsch–Gordan operations of rows $n - 1$ and 1.

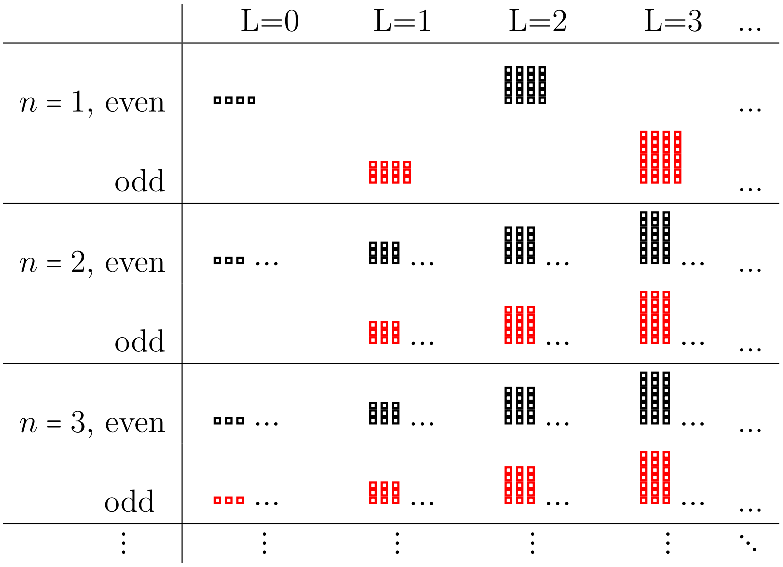


At $L = l$ these are in case...

1 : $c$–tuple of vectors of dimension $2l+1$. These are the sum of the spherical harmonics $Y_l(\boldsymbol{r})$ summed over all points $\boldsymbol{r}$ of color $i = 1, 2, ..., c$.

and in cases 2,3 depending on the function class in case...

i : Infinitely many vectors of dimension $2l+1$, sums of $|\boldsymbol{r}|^{2k} \cdot Y_l(\boldsymbol{r})$ of points of one color.

ii : Infinitely many vectors of dimension $2l+1$, sums of $g_i(|\boldsymbol{r}|) \cdot Y_l(\boldsymbol{r}/|\boldsymbol{r}|)$ of points of one color.

The $n$–th row consists of the results of all Clebsch–Gordan operations of rows $n-1$ and 1. In each row $n > 1$ at column $L = l$ there are infinitely many vectors of dimension $2l+1$.

We can now prove:

**Theorem 3** (Algebraic completeness when using Spherical Harmonics and Clebsch–Gordon operations)**.**

1. *All scalar PPSDs are some linear combination of fields in this schema.*
2. *Any* $SO(3)$–covariant *PPSD with values in* $\mathcal{H}^{(l)}$ *is a linear combination of vectors in the* $l$*–th column.*
3. *Any* $O(3)$–covariant *PPSD with values in* $\mathcal{H}^{(l)}$ *is a linear combination of vectors in the* $l$*–th column of the appropriate parity.*
4. *For case i (polynomial functions), the fundamental features* $\sum_{\boldsymbol{r}\in S_\gamma} |\boldsymbol{r}|^{2k} \cdot Y_l(\boldsymbol{r})$ *are polynomials of degree* $2k+l$ *in the coordinates, and in each Clebsch–Gordan operation the degrees of the polynomials add up to the new degree. To distinguish sets of* $n$ *points in* $\mathbb{R}^3$ *that are not equivalent under* $O(3)$ *it is enough to use the invariants of degree* $< n^2 \cdot (n+1)^2/4$*, and for* $G = SO(3)$ *we get a similar bound that is* $O(n^6)$*.*

The first three parts of this theorem ares valid for all cases 1,2i,2ii,3i,3ii. For a proof of them (and details for part 3) see Appendix K. The fourth part is actually part of theorem 4, see

Appendix L, we only copied it here since it can as well be applied in the context of this theorem and gives an explicit form of the (first part of the) finiteness theorem.

The first two parts of the topological completeness theorem can be expressed in this schema using the values of the functions on configurations:

- Two colored point sets have the same first row if and only if they are equal.
- Two colored point sets have the same first column if and only if they are $SO(3)$–equivalent.

In this schema, the $(n+1)$–body information appears in row $n$. So the results of[2] show that the first 3 rows are not enough to distinguish all $SO(3)$–equivalence classes of colored point sets (even these are infinitely many invariants). However, Theorems 1 and 3 together show that without the restriction of rows we can distinguish all equivalence classes.

There is in fact a different route to describe $SO(3)$–invariant PPSDs that does not use the decomposition into irreducible components. This starts with the moment tensors $\sum_{\boldsymbol{r}\in S_\gamma} \boldsymbol{r}^{\otimes k}$ as fundamental features, and then applies tensor products and contractions. For $G = SO(3)$, also the 3-tensor giving the orientation, i.e. the Levi-Civita symbol $\epsilon_{ijk}$ is allowed in the tensor products. If any such sequence of tensor products and contractions arrive at a scalar, this must be an $SO(3)$–invariant feature of the colored point set, and one can show:

**Theorem 4** (Algebraic completeness for tensor contraction features)**.**

1. *Any $O(3)$–invariant PPSD is a linear combination of features obtained by multiplications / contractions of moment tensors.*
2. *Any $SO(3)$–invariant PPSD is a linear combination of features obtained by multiplication / contractions of moment tensors and optionally one Levi–Civita symbol.*
3. *To distinguish $O(3)$–inequivalent point sets of $n$ points in $\mathbb{R}^3$, it is enough to multiply / contract up to $n$ tensor moments. Alternatively, it is enough to multiply / contract*

*tensor moments of orders* $\leq n$.

*As another option, we can also restrict the total degree of the invariants to be less than* $n^2 \cdot (n+1)^2/4$. *For* $G = SO(3)$ *we get an upper bound on the degrees that is* $O(n^6)$.

We formulated this theorem for case i only (polynomial functions).

The first statement for invariant scalar polynomial functions in a fixed number of points and $|\mathcal{C}| = 1$ was proved in.[3] The first two statements for invariant scalars follow directly from the First Fundamental Theorems of Invariant Theory for $G = O(3)$ and $G = SO(3)$ once we have the uniqueness of the Normal Form. The third statement follow from the facts that the first $n$ moment tensors characterize configurations of $n$ points up to $O(3)$, that sums over $k$–tuples of points for $k > n$ can be rewritten as linear combinations of other sums over $\leq n$–tuples of points, and from Göbel's theorem on invariants of permutation groups. See Appendix L for full proofs.

This approach gives a much smaller list of invariants than Theorem 3, but that is mainly an advantage for $|\mathcal{C}| = 1$, which is less relevant for applications in chemistry. In the straightforward implementation the tensor product / contraction features are expensive to calculate, we will not make use of them in the rest of the paper; we only use this theorem to get the explicit finiteness statement for the previous theorem.

# E Pseudo code for algorithm

When we want to learn an invariant property $f(\{(\boldsymbol{r}_i, \gamma_i)\})$ of colored point sets, we can use different Machine Learning algorithms to match it to parameterized functions $g(\{(\boldsymbol{r}_i, \gamma_i)\}, \theta)$ for a collection of parameters $\theta$. For example, for Stochastic Gradient Descent we would start with random initial parameters $\theta_0$, and then in each step improve the $\theta$ to minimize a loss function of $\theta$ that depends on how close the $g(\{(\boldsymbol{r}_i, \gamma_i)\}, \theta)$ match the $f(\{(\boldsymbol{r}_i, \gamma_i)\})$ for the configurations in a training set. The description in the main part gives invariant / covariant

features of configurations $\{(\boldsymbol{r}_i, \gamma_i)\}$ that can be used to construct such parameterized functions $g(\{(\boldsymbol{r}_i, \gamma_i)\}, \theta)$. In the following, we give pseudo code for an implementation of the simplest such parameterized function, which just computes a linear combination of invariant features.

Unless we have some special information that the true function $f$ must be homogeneous of a certain degree in the fundamental features, we will in general use $n_{mat} > 1$ matrix products with different lengths $b_i$, to be able to mix invariants of different body order.
Since we want invariants, the covariants of form (3) should actually be scalars, i.e. the output is in $\mathcal{H}^{(a_m)}$ with $a_m = 0$, so the products look like

$$M_{a_{m-1},0,l_m}(\gamma_m) \cdot \ldots \cdot M_{a_1,a_2,l_2}(\gamma_2) \cdot M_{0,a_1,l_1}(\gamma_m)$$

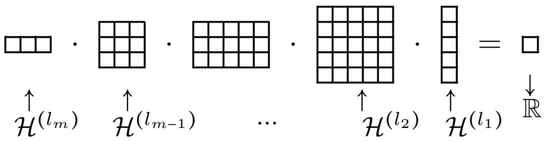


In a concrete implementation, we can choose to re–use some of the computations for different invariants. Here we will do this in a simple way – we re–use the matrix product in the middle for $n_{vec}$ different pairs of vectors at both ends.
Bundling $n_{vec}$ vectors of size $2l_1 + 1$ on the right end can be expressed by removing the first vector and using a $(2l_1 + 1) \times n_{vec}$ matrix $V$ on the right:

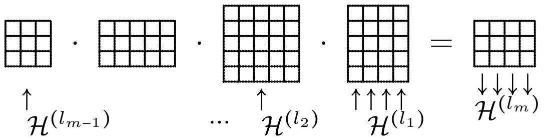


Each of the resulting $n_{vec}$ vectors in $\mathcal{H}^{(l_m)}$ can then be paired with each of another $n_{vec}$ vectors in $\mathcal{H}^{(l_m)}$ to give $n_{vec}^2$ scalar products.

To apply the "matrix of matrices" approach, we pick lengths $2l_i+1$ for $i = 1, 2, ..., r$ which

then leads to vectors of length $K$ and $K \times K$ matrices with

$$K := (2l_1 + 1) + (2l_2 + 1) + ...(2l_r + 1).$$

In the Algorithm 1 outlined below these $K$–dimensional vectors are computed in step 2 as linear combinations (with learnable coefficients) of the fundamental features computed in step 1. The $K \times K$–matrices are computed in step 3.
In step 4 we compute the matrix products of $b_i$ $K \times K$–matrices with $n_{vec}$ $K$–dimensional vectors collected in a $K \times n_{vec}$ matrix $V$. The resulting $n_{vec}$ $K$–dimensional vectors are then decomposed into $n_{mat} \cdot n_{vec}$ vectors of dimensions $2l_i + 1$ for $i = 1, 2, ..., r$, taking the scalar products with $n_{vec}$ vectors of dimensions $2l_i + 1$ for $i = 1, 2, ..., r$ then gives $n_{mat} \cdot r \cdot n_{vec}^2$ invariants.
In the final step 5, we use some more parameters to output a linear combination of the invariants. The completeness theorems say that such linear combinations are already capable of approximating any invariant feature, and we will use this also in a concrete application.

In the main part we also mentioned another way to extract invariant scalars from a covariant matrix of matrices: Take the traces of the square sub–matrices. This is implemented in `E3x`, together with methods to build up a matrix of matrices from vectors in irreducible representations. This variant of Algorithm 1 (for simplicity, for one matrix product) would then be formulated with `E3x` as:

```
from e3x.matrix import matmat
from e3x.so3 import irreps
from jax import numpy as jnp

def f(params, conf, max_degree, ls, mult, n_factors, shift_by_id):
  """Function approximation by matrix products.

  Args:
    params: List of parameters.
```

**Algorithm 1:** Parameterized $SO(3)$–invariant

**Input:** Points $\boldsymbol{r}_i \in \mathbb{R}^3$ of color $\gamma_i \in \mathcal{C}$ for $i = 1, ..., n$

**Hyperparameters:**

Number of vectors for scalar products: $n_{vec}$

Number matrix products $n_{mat}$

Integers $0 \leq l_1 \leq ... \leq l_r$ corresponding to matrix sides $2l_i + 1$ of submatrices.

For $i = 1, ..., n_{mat}$: Integers $b_i \geq 0$ (corresponding to body orders $b_i + 2$).

**Parameters:** Coefficients for linear combinations in vectors, matrices, and combined invariants.

**Compute:**

1. Spherical harmonics:
   For $l = 0, ..., 2l_r$, $\gamma \in \mathcal{C}$ set $Y_l(\gamma) \coloneqq \sum_{\boldsymbol{r} \in S_\gamma} Y_l(\boldsymbol{r})$
2. Vectors:
   For $i = 1, ..., r$:
   Compute $2 \cdot n_{mat} \cdot n_{vec}$ linear comb. of the $Y_{l_i}(\gamma)$
3. Matrices:
   For $(a, b) \in \{l_1, ..., l_r\}^2$:
   Compute $b_1 + ... + b_{n_{mat}}$ matrices of shape $a \times b$ by linear combinations of $\iota_{a,b,l}(Y_l(\gamma))$ for $l = |a - b|, ..., a + b$.
   Assemble them to $b_1 + ... + b_{n_{mat}}$ square matrices of shape $l_1 + ... + l_r$.
4. Products:
   For $i = 1, ..., n_{mat}$:
   Assemble $n_{vec}$ column vectors from 2. into $V$, use matrices from 3. to compute products $W \coloneqq M_1 \cdot ... \cdot M_{b_i} \cdot V$.
   Take all scalar products of irreducible parts of columns of $W$ with vectors from 2.
   This gives $n_{mat} \cdot r \cdot n_{vec}^2$ invariants.
5. **Output:**
   Linear combination of these invariants.

```
    conf:    Float[n_colors, n_points, 3]  Configuration of points
    l_max:   Maximal degree (L) of irreducibles in the matrices.
    ls:      List of L's for the submatrices.
    mult:    Multiplicity of each L.
    n_factors: Number of factors in matrix product.
    shift_by_id: Shift matrix multiplication by identity matrix.

  Returns:
    Estimated function.
  """
  sh = irreps.spherical_harmonics(conf, max_degree=max_degree)
  sum_sh = jnp.sum(sh, axis=1)            # [n_colors, (L+1)**2]
  sh_features = jnp.transpose(sum_sh)     # [(L+1)**2, n_colors]
  primary_features = matmat.combine_irreps(sh_features, params[0], 'high')
  product = matmat.make_square_matrix(primary_features, ls, mult, max_degree,
                                      shift_by_id, 'high')
  for i in range(1, n_factors):
    primary_features = matmat.combine_irreps(sh_features, params[i], 'high')
    matrix_features = matmat.make_square_matrix(primary_features, ls, mult,
                                                 max_degree, shift_by_id, 'high')
    product = jnp.matmul(product, matrix_features, precision='high')
  prod_traces = matmat.get_traces(product, ls, mult, shift_by_id)
  result = jnp.dot(prod_traces, params[-1], precision='high')
  return result
```

and the parameters would be initialized by

```
def init_params(
    key, ls, mult, max_degree, n_colors, n_factors,
    factor_mat, factor_final
):
  keys = jax.random.split(key, n_factors + 1)
  dict_irreds = matmat.make_dict_irreps_mult(ls, max_degree = max_degree)
  init = matmat.init_matrix_irreps_weights
  params = [
      init(keys[i], n_colors, dict_irreds, mult, factor_mat)
      for i in range(n_factors)
  ]
  params.append(
      jax.random.normal(keys[-1], (len(ls) * mult**2,)) * factor_final
  )
  return params
```

where `factor_mat` would need to be adjusted such that the product of matrices does not go to infinity or zero. It is easier and more efficient to use a product of matrices "shifted by the identity matrix"

$$(Id + A_1) \cdot (Id + A_2) \cdot \ldots \cdot (Id + A_n) - Id$$

where the $A_i$ are initially small. Here the identity matrices $Id$ are similar to skip connections in ResNets[4] and allow a smooth learning of all factors, even for large $n$. In the code using `E3x` both methods are supported: The original matrix product corresponds to setting the bool parameter `shift_by_id` to `False`, for the second method it would be set to `True`.

We have pointed out that both the Clebsch–Gordan operation and matrix multiplication define maps

$$\left(\mathcal{H}^{(0)} \oplus \ldots \oplus \mathcal{H}^{(l)}\right) \otimes \left(\mathcal{H}^{(0)} \oplus \ldots \oplus \mathcal{H}^{(l)}\right) \;\rightarrow\; \mathcal{H}^{(0)} \oplus \ldots \oplus \mathcal{H}^{(l)}$$

which are used to build up different linear combinations of covariants of higher body order, and that while the Clebsch–Gordon maps are of complexity $O(l^6)$, the matrix multiplication is of complexity $O(l^3)$. However, when we use the matrix multiplication approach, we also need to convert spherical harmonics of degrees $0, \ldots, l$ into a $(l+1) \times (l+1)$–matrix (and similar for rectangular matrices). While this can be efficiently encoded as a tensor contraction (`einsum` in JAX), it is theoretically of order $O(l^4)$. In theory, this can be avoided using complex spherical harmonics, which makes this also an $O(l^3)$ operation. However, for moderate $l$ it seems the simple and efficient tensor operation is preferable to the more complicated operation using complex numbers, so in `E3x` we use the simple implementation.

While these linear combinations of step 5 of the outlined Algorithm 1 are enough in theory, it is very easy and may be beneficial in practical applications to add e.g. a neural network computation on top of the invariants to further increase the flexibility of the resulting functions.

Further easy flexibility increases could be obtained (if necessary) by parameterized operations on the partial matrix products $M_1 \cdot \ldots \cdot M_j$, e.g. by using linear combinations of different

$a \times b$ submatrices and the transpose of $b \times a$ submatrices or by multiplying $a \times b$ submatrices with learnable non-linear scalar functions of the traces of $c \times c$ submatrices.
Other possible operations include fast implementations of transcendental matrix functions like $exp$ on $a \times a$ submatrices, and linear combinations of the individual $\mathcal{H}^{(l)}$ components of different submatrices, as implemented by the `FusedTensor` layers in `E3x` for $r = 1$.

# F Incompleteness of 3 body functions

An $SO(3)$–invariant PPSD of order $m$ (i.e. a $(m+1)$ body function) gives the same value on point sets $S, S'$ if their (multi–)set of "subsets of cardinality $m$ modulo $G$" is the same.

To illustrate this in the case $m = 2$ note that 2–element subsets of $\mathbb{S}^2$ modulo $SO(3)$ are given by the angle between them (or, equivalently, their scalar product).
So $SO(3)$–invariant 3 body functions on $\mathbb{S}^2$ only depend on the (multi–)*set* of angles between the points.

We now give examples of point configurations on the circle $\mathbb{S}^1 \subset \mathbb{S}^2$ that are substantially different, but still have the same set of angles.
For the first example, we divide the unit circle into 30 equal angles and position points at a subset of these 30 angles, so the points are given by a residue class modulo 30.
The points at

$$0, 1, 8, 11, 13 \qquad \text{or} \qquad 0, 10, 11, 13, 18$$

give the same *set* of differences mod 30 (each difference occurs only once):

$$\{1, 2, 3, 5, 7, 8, 10, 11, 12, 13\}$$

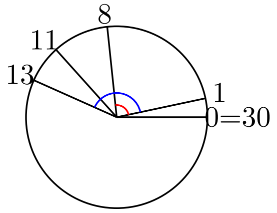


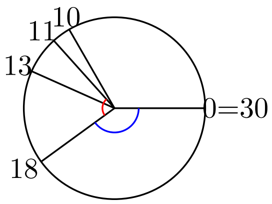


While this may look like some coincidence, there are actually whole submanifolds of pairs of configurations that cannot be distinguished, here is the simplest example:

The four points at angles $0, \alpha, \alpha + \frac{\pi}{2}, \pi$ or $0, \alpha, \pi, \alpha - \frac{\pi}{2}$ give the same *set* of angles between them: $\left\{\alpha, \frac{\pi}{2} + \alpha, \frac{\pi}{2} - \alpha, \pi - \alpha, \frac{\pi}{2}, \pi\right\}$

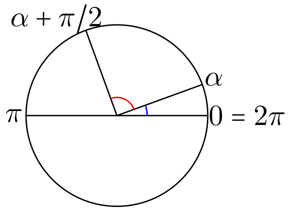


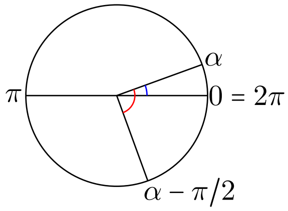


For more examples and a closer analysis, see.[5]

# G Some background from representation theory

We collect here some notations and remarks about representation theory. Most of the time we focus on representations on real vector spaces, but for the comparison between matrix products and Clebsch–Gordan operations we will also make use of complex representations.

An elementary introduction to representations over the real numbers, tailored to our purposes, can be found in,[6] we only repeat here some notations and point out some special details.

## G.1 The irreducible representations $\mathcal{H}^{(L)}$ of $SO(3)$

There is one irreducible representation of $SO(3)$ of degrees $2L{+}1$ for $L = 0, 1, 2, ...$ given by the harmonic homogeneous polynomials of total degree $L$ in three variables $x, y, z$; we denote

this real vector space by $\mathcal{H}^{(L)}$. Every irreducible representation over $\mathbb{R}$ is isomorphic to exactly one of these $\mathcal{H}^{(L)}$, and likewise every irreducible representation over $\mathbb{C}$ is isomorphic to exactly one of the corresponding complex representations $\mathcal{H}^{(L)} \otimes_{\mathbb{R}} \mathbb{C}$.

One particular basis of the vector space $\mathcal{H}^{(L)}$ is given by the real–valued spherical harmonics $Y_l^m$ with $m = -l, ..., l-1, l$.

## G.2 Equivariant maps $\mathbb{S}^2 \to \mathcal{H}^{(L)}$

Let $(\rho_L, \mathcal{H}^{(L)})$ be an irreducible representation of $SO(3)$. An $SO(3)$–equivariant function $f : \mathbb{S}^2 \to \mathcal{H}^{(L)}$ is already determined by its value at one point, e.g. at $(0,0,1)^T$, since for every other point $\boldsymbol{u} \in \mathbb{S}^2$ there is a rotation $g \in SO(3)$ that moves $(0,0,1)^T$ to $\boldsymbol{u}$. Furthermore, the point $(0,0,1)^T \in \mathbb{S}^2$ is the fixed point of the subgroup $T \subset SO(3)$ of rotations around the $z$–axis. So it must be mapped by the equivariant $f$ to a fixed point of the group $\rho_L(T)$ in $\mathcal{H}^{(L)}$. However, this fixed point set in $\mathcal{H}^{(L)}$ is only a 1-dimensional vector space: Over $\mathbb{C}$ a basis of the $2l+1$–dimensional $\mathcal{H}^{(L)}$ is given by the $2l+1$ complex spherical harmonics of weight $-l, ..., l-1, l$ (see N.3), only the subspace of weight 0 is invariant under $T$, over $\mathbb{R}$ it is given by the real spherical harmonics $Y_l^0$.

Therefore, $(0,0,1)^T$ must be mapped to a point on that line, so all equivariant maps $f : \mathbb{S}^2 \to \mathcal{H}^{(L)}$ are the same up to a multiplicative factor.

## G.3 Schur's lemma

Given representations with decompositions into irreducibles, Schur's lemma describes what the maps between those representations can be. It is almost trivial to prove, but has powerful consequences. We give a proof here since usually it is only formulated and proven for complex vector spaces, but we want to apply it also to real representations of $SO(3)$.

**Lemma 4** (Schur's lemma)**.** *Let $V, W$ be $K$–vector spaces for $K = \mathbb{R}$ or $K = \mathbb{C}$, and let $\rho_1 : G \to GL(V)$, $\rho_2 : G \to GL(W)$ be* **irreducible** *representations of $G$, and $f : V \to W$ a*

*covariant linear map.*

1. *$f$ is either 0 or bijective.*

2. *If these representations are not isomorphic, $f = 0$.*

3. *If $V$ is a finite dimensional $\mathbb{C}$–vector space, the only covariant linear maps $f : V \to V$ are the $f = \lambda \cdot Id$ for some $\lambda \in \mathbb{C}$.*

4. *If $V$ is an* **odd dimensional** *$\mathbb{R}$–vector space, the only covariant linear maps $f : V \to V$ are the $f = \lambda \cdot Id$ for some $\lambda \in \mathbb{R}$.*

*Proof.*

1. Kernel and image must be mapped by $G$ into themselves, so they must be $\{0\}$ or the full space. If $ker(f) = V$ or $im(f) = \{0\}$, we have $f = 0$. Otherwise, we must have $ker(f) = \{0\}$ and $im(f) = W$, i.e. $f$ is bijective.

2. Follows from 1: If $f$ is bijective, there is an inverse $f^{-1}$ and these define an isomorphism.

3. Over $\mathbb{C}$, $f$ must have some eigenvalue $\lambda$ with eigenvector $\boldsymbol{v} \neq 0$. Then $f - \lambda Id$ is again a covariant linear map; since $(f - \lambda \cdot Id)\boldsymbol{v} = 0$, it is not bijective, so it must be 0, i.e. $f = \lambda \cdot Id$.

4. Over $\mathbb{R}$, the (multi-) set of eigenvalues of $f$ (with multiplicities) must be invariant under complex conjugation, since its cardinality $dim(V)$ is odd, there must be at least one real eigenvalue $\lambda$. Then we can apply the same argument as in 3.

□

The last part is no longer true for even dimensions:

The two-dimensional representation $\rho : SO(2) \to GL(2), g \mapsto g$ is irreducible, but any rotation is also $G$–covariant.

However, since all irreducible representations of $SO(3)$ are odd–dimensional, we can apply 4. as a replacement of 3.

**Corollary 5.** *Let $\mathbb{K}$ be $\mathbb{R}$ or $\mathbb{C}$, and let $\rho : G \to GL(V)$, $\rho' : G \to GL(V')$ be representations that can be decomposed into irreducibles:*

$$V \simeq V_1^{\oplus m_1} \oplus V_2^{\oplus m_2} \oplus \ldots \oplus V_n^{\oplus m_n} \tag{2}$$

*and*

$$V' \simeq V_1^{\oplus m'_1} \oplus V_2^{\oplus m'_2} \oplus \ldots \oplus V_n^{\oplus m'_n} \tag{3}$$

*with $V_1, .., V_n$ different (i.e. non-isomorphic) irreducible representations and*

$$V_i^{\oplus m_i} := V_i \oplus \ldots \oplus V_i \qquad \textit{sum of } m_i \textit{ copies of } V_i.$$

*(We allow exponents to be 0 to be able to use the same $V_i$, with $V_i^{\oplus 0} := \{0\}$.) If $\mathbb{K} = \mathbb{R}$, also assume that the $\dim(V_i)$ are odd. Then the vector space of covariant maps $V \to V'$ can be described by a vector space isomorphism*

$$Hom[(\rho, V), (\rho', V')] \simeq \bigoplus_{\substack{i=1 \\ m_i>0, m'_i>0}}^{n} M_{m_i, m'_i}(\mathbb{K}) \tag{4}$$

Among the many applications, we mention:

**Lemma 6.** *Let $V$ be an odd dimensional vector space over $\mathbb{R}$ and $\rho : G \to GL(V)$ an irreducible representation. Then the invariant scalar product on $V$ is unique up to a factor.*

*Proof.* Assume the invariant scalar products for $j = 1, 2$ are given by matrices $A_j$ as $\langle \boldsymbol{v}, \boldsymbol{w} \rangle_j = \boldsymbol{v}^T A_j \boldsymbol{w}$.

Then being invariant under $G$ means for the matrices that $\rho(g)^T A_j \rho(g) = A_j$. Then we get for the matrix $A_1^{-1} \cdot A_2$ the invariance

$$\begin{aligned} A_1^{-1} \cdot A_2 &= \rho(g)^{-1} A_1^{-1} \rho(g)^{-T} \cdot \rho(g)^T A_2 \rho(g) \\ &= \rho(g)^{-1} \left( A_1^{-1} A_2 \right) \rho(g) \end{aligned}$$

which means $A_1^{-1}A_2$ is a covariant map. So by part 4 of Schur's lemma above $A_1^{-1} \cdot A_2$ is a scalar multiple of the identity. $\square$

## G.4 Isotypic decomposition

**Definition 1** (Isotypic component)**.**
*Let $\mathbb{K}$ be either $\mathbb{R}$ or $\mathbb{C}$, and let $W$ be a finite dimensional $\mathbb{K}$–vector space, and $\rho : G \to GL(W)$ a representation of a group $G$.*
*For each irreducible representation $\lambda : G \to GL(V_\lambda)$ write $W_\lambda$ for the subspace of $W$ generated by the* $\mathrm{Im}(f)$ *for covariant maps $f : V_\lambda \to W$, it is called the isotypic component of $\lambda$ in $W$.*

Assume now $G$ is compact, then there exists a decomposition of $W$ into irreducible components

$$(W, \rho) \simeq (W_1, \rho_1) \oplus \ldots \oplus (W_m, \rho_m) \tag{5}$$

Then all $(W_i, \rho_i) \simeq (V_\lambda, \lambda)$ are contained in $W_\lambda$ since we have the covariant inclusion maps

$$f : V_\lambda \simeq W_i \hookrightarrow W \qquad \text{with} \quad \mathrm{Im}(f) = W_i.$$

So the sum of the $(W_i, \rho_i) \simeq (V_\lambda)$ are contained in $W_\lambda$.

Using Corollary 5 we can see that $W_\lambda$ cannot be larger:

**Corollary 7.** *Let $G$ be a compact group, $\mathbb{K} = \mathbb{R}$ or $\mathbb{C}$, and $\rho : G \to GL(W)$ a finite dimensional representation over $\mathbb{K}$. If $\mathbb{K} = \mathbb{R}$, also assume all irreducible representations are odd dimensional. Then for any decomposition* (5) *the isotypic components $W_\lambda$ are the sum of the $(W_i, \rho_i) \simeq (V, \lambda)$.*

*Proof.* For any covariant map $f : V \to W$ we have maps

$$\begin{array}{ccccc} \boldsymbol{v} & V & \xrightarrow{f_i} & W_i & \boldsymbol{w}_i \\ \downarrow & {\scriptstyle f}\downarrow & & \uparrow{\scriptstyle \pi_i} & \uparrow \\ f(\boldsymbol{v}) & W & \xrightarrow{\simeq} & W_1 \oplus \ldots \oplus W_m & \boldsymbol{w}_1 + \ldots + \boldsymbol{w}_m \end{array}$$

and can decompose the map $f$ as

$$f(\boldsymbol{v}) = f_1(\boldsymbol{v}) + f_2(\boldsymbol{v}) + \ldots + f_m(\boldsymbol{v}), \qquad f_i(\boldsymbol{v}) \in W_i \subseteq W$$

If $\rho_i$ is not isomorphic to $\lambda$, then by Schur's lemma $f_j = 0$, so $V_\lambda$ can also not be larger than the sum of all $W_i$ with $\rho_j \simeq \lambda$.
So $V_\lambda$ is exactly the sum of all $W_i$ with $\rho_i \simeq \lambda$. □

This means that we get for representations of a compact group an isotypic decomposition:

**Corollary 8.** *Let $G, W, \rho$ as before, and let $\{\lambda_1, \ldots, \lambda_k\}$ be the set of (isomorphism classes of) irreducible representations of $G$ for which $W_{\lambda_i} \neq 0$.*
*Then*

$$W = W_{\lambda_1} \oplus \ldots \oplus W_{\lambda_k} \tag{6}$$

Note that this decomposition is unique (up to the order in which we write the direct sum, of course), whereas (5) was not.

## G.5 Dual, tensor product, and Hom

Given a representation $(\rho, V)$ over $\mathbb{K} = \mathbb{R}$ or $\mathbb{C}$, the dual space $V^*$ is the vector space of linear maps $L : V \to \mathbb{K}$, on it the dual representation $(\rho^*, V^*)$ is defined by

$$\rho^*(g)L := \Big(v \mapsto L(g^{-1}x)\Big).$$

For $v \in V, L \in V^*$ we also write $\langle L, v \rangle$ for $L(v)$.

The tensor product of representations $(\rho^V, V), (\rho^W, W)$ is a representation on the vector space $V \otimes W$. This vector space is spanned by expressions $v \otimes w$, if $v$ and $w$ go over bases of $V, W$, the $v \otimes w$ go through a basis of $V \otimes W$. The group operation on $V \otimes W$ has the

property

$$\rho(g)(v \otimes w) = \rho^V(g)(v) \otimes \rho^W(g)(w).$$

For slightly more details and examples see e.g.[6]

For two representations $(\rho^V, V), (\rho^W, W)$ we can form the vector space $Lin(V, W)$ of linear maps $V \to W$, this is a representation under

$$\rho^{VW}(g)f := \Big(v \mapsto \rho^W(g)f(\rho^V(g^{-1})v)\Big)$$

With these definitions of representations, the matrix multiplication

$$Lin(U, V) \times Lin(V, W) \to Lin(U, W)$$

for representations $(\rho^U, U), (\rho^V, V), (\rho^W, W)$ and linear maps $f : U \to V, h : V \to W$ is a covariant map:

$$\begin{aligned}
&\rho^{UW}(h \circ f)(u) \\
&\quad = \rho^W(g)\ hf\Big(\rho^U(g^{-1})u\Big) \\
&\quad = \rho^W(g)\ h\Big(\rho^V(g^{-1})\ \rho^V(g)\ \ f\Big(\rho^U(g^{-1})u\Big)\Big) \\
&\quad = \Big(\rho^{VW}(g)(h)\Big)\ \Big(\rho^{UV}(g)(f)\Big)\ (u)
\end{aligned}$$

If $V$ or $W$ are finite dimensional, the notions of $Lin$, $\otimes$ and dual representations are related by the isomorphism of representations

$$V^* \otimes W \simeq Lin(V, W)$$

which maps $v^* \otimes w$ to the linear map $v \mapsto \langle v^*, v\rangle w$.

(We are usually only interested in finite dimensional representations, but this makes also

sense for $W$ the infinite dimensional space of all PPSDs and $V$ a finite dimensional representation. If both $V$ and $W$ are infinite dimensional, this algebraic isomorphism is no longer true: For $V = W$ the identity in $Lin(V,V)$ cannot be given by a finite linear combination of maps of rank 1.)

The homomorphisms in the category of $G$–representations are the covariant maps, they are just the $G$–invariant elements of the representation $Lin(V,W)$:

$$Hom_G(V,W) = Lin(V,W)^G = (V^* \otimes W)^G$$

## G.6 Function spaces

Let $X$ be a set on which $G$ acts, then $G$ also acts on the set $Map(X,\mathbb{R})$ of functions $f : X \to \mathbb{R}$ by

$$\rho(g)f := \Big(x \mapsto f(g^{-1}x)\Big).$$

The set of $G$–fixed points $Map(X,\mathbb{R})^G$ is just the set of invariant functions $X \to \mathbb{R}$.

More generally, for a representation $(\rho^V, V)$ of $G$, the group $G$ also acts on the set $Map(X,V)$ by

$$\rho(g)f := \Big(x \mapsto \rho^V(g)\big(f(g^{-1}x)\big)\Big).$$

The fix point set $Map(X,V)^G$ is the set of all $f$ for which for all $x \in X, g \in G$

$$\rho^V(g)\big(f(g^{-1}x)\big) = f(x)$$

or equivalently $f(gx) = \rho^V(g)f(x)$ for all $x \in X, g \in G$; so this is exactly the set of covariant maps $X \to V$.

We can map the tensor product $V \otimes Map(X,\mathbb{R})$ bijectively to $Map(X,V)$ by requiring

$$\iota(v \otimes f) : x \mapsto f(x) \cdot v,$$

applying this to $v_i \otimes f_j$ for bases $v_i$ of $V$ and $f_j$ of $Map(X, \mathbb{R})$ we can see that this gives an isomorphism

$$\iota : V \otimes Map(X, \mathbb{R}) \xrightarrow{\sim} Map(X, V).$$

of $G$–representations. Similarly, we have

$$Lin(V, Map(X, \mathbb{R})) \simeq Map(X, V^*),$$

an isomorphism $\iota$ is given by setting the image of $L : V \to Map(X, \mathbb{R})$ to

$$\iota(L) : X \to V^*, x \mapsto \big(v \mapsto L(v)(x)\big)$$

and this is compatible with the operation of $G$.

## G.7 Complex representations and scalar products

The aim of this subsection is to clarify a potentially confusing identification of a representation $\rho$ with its dual $\rho^*$. Our irreducible representations $\mathcal{H}^{(l)}$ are defined over the real numbers (are given by real valued matrices with respect to the basis of the real spherical harmonics), but we can also consider the $2l+1$–dimensional vector space on which these $(2l+1)\times(2l+1)$–matrices operate as complex vector spaces on which we then have the additional structure of complex conjugation.

We fix a scalar product on the real vector space that is invariant under $\rho(G)$. In general, if we have a real vector space $V$ with scalar product $\langle\, , \rangle$, we have two different notions of scalar product on its complexification $V \otimes \mathbb{C}$: The algebraic scalar product, which in an orthonormal basis of $V$ (which identifies $V \otimes \mathbb{C}$ with $\mathbb{C}^d$) is given by

$$\langle \boldsymbol{w}, \boldsymbol{v} \rangle_{alg} := \boldsymbol{w}^T \cdot \boldsymbol{v}$$

and the usual Hermite scalar product

$$\langle \boldsymbol{w}, \boldsymbol{v} \rangle := \langle \overline{\boldsymbol{w}}, \boldsymbol{v} \rangle_{alg} = \boldsymbol{w}^H \cdot \boldsymbol{v}.$$

If the real representation $\rho$ was orthogonal on $V$, it will be unitary on $V \otimes \mathbb{C}$ with respect to this Hermite scalar product.

For a representation $(V, \rho)$ on vector spaces over any field $\mathbb{K}$, the dual representation $(V^*, \rho^*)$ is defined to operate on linear forms $L : V \to \mathbb{K}$ in $V^*$ by

$$\rho^*(g)L := \Big(\boldsymbol{v} \mapsto L\big(\rho(g^{-1})\boldsymbol{v}\big)\Big)$$

The algebraic scalar product defines a vector space isomorphism between $V$ and its dual $V^*$: We represent the linear form $L$ by a vector $\boldsymbol{w} \in V$ such that $L(\boldsymbol{v}) = \langle \boldsymbol{w}, \boldsymbol{v} \rangle_{alg}$. For the representation $\rho^*$ this means we have

$$\langle \rho^*(g)\boldsymbol{w}, \boldsymbol{v} \rangle_{alg} = \langle \boldsymbol{w}, \rho(g^{-1})\boldsymbol{v} \rangle_{alg} = \langle (\rho(g)^{-1})^T \boldsymbol{w}, \boldsymbol{v} \rangle_{alg}$$

for all $\boldsymbol{v}, \boldsymbol{w}$, and for a unitary representation this is equivalent to $\rho^*(g) = (\rho(g)^H)^T = \overline{\rho(g)}$. So for a scalar product on $V$ that is invariant under $\rho(G)$, this representation is unitary and the dual representation to $(V, \rho)$ is just given by the complex conjugate $\bar{\rho}$. Since our representations $\mathcal{H}^{(l)}$ are actually already defined over $\mathbb{R}$, this representation is isomorphic (as an abstract representation) to its dual. (If we choose an orthonormal basis of the real vector space, $\rho$ and $\rho^*$ are given by the same matrices, and for arbitrary complex bases the isomorphism can be realized by the base change that maps the complex basis vectors to their complex conjugate.) While we are free to choose any complex basis of $\mathcal{H}^{(l)}$, we have to keep this basis for both representations $\rho$ and $\rho^*$, so we have to distinguish them when they both occur in the same computation.

From two of our representations $(\mathcal{H}^{(l_1)}, \rho_1)$, $(\mathcal{H}^{(l_2)}, \rho_2)$ we get a representation on $(2l_2 + 1) \times$

$(2l_1+1)$–matrices by identification with the linear maps $\mathcal{H}^{(l_1)} \to \mathcal{H}^{(l_2)}$, on which $G = O(3)$ operates by

$$\begin{aligned} \left(f : \mathcal{H}^{(l_1)} \to \mathcal{H}^{(l_2)}\right) &\quad \mapsto \\ \rho_{Lin}(g)(f) \quad : &\quad \mathcal{H}^{(l_1)} \to \mathcal{H}^{(l_2)}, \boldsymbol{v} \mapsto \rho_2(g) f(\rho_1(g^{-1}) v) \end{aligned}$$

When we choose bases of $\mathcal{H}^{(l_1)}$ and $\mathcal{H}^{(l_2)}$, the resulting operation on $(2l_2+1) \times (2l_1+1)$–matrices $A$ is given by

$$A \mapsto \rho_2(g) \cdot A \cdot \rho_1(g)^{-1} = \rho_2(g) \cdot A \cdot \rho_1(g)^H.$$

We get an isomorphism of representations $Lin(\mathcal{H}^{(l_1)}, \mathcal{H}^{(l_2)}) \simeq \mathcal{H}^{(l_2)} \otimes \mathcal{H}^{(l_1)^*}$ by mapping

$$\boldsymbol{v}_2 \otimes \boldsymbol{v}_1^* \mapsto \left(\mathcal{H}^{(l_1)} \to \mathcal{H}^{(l_2)}, \boldsymbol{r} \mapsto \boldsymbol{v}_2 \cdot \langle \boldsymbol{v}_1^*, \boldsymbol{r} \rangle_{alg}\right)$$

# H Polynomial point set descriptors (PPSDs)

In this section we only consider PPSDs in the original, narrow meaning, i.e. we only consider case i (polynomial functions), not case ii (general radial basis functions).

## H.1 PPSDs and their Normal Form

Just in case the informal description of PPSDs leaves room for ambiguity, we give here a version of increased formality:

**Definition 2** (PPSD).
*Fix a finite set $\mathcal{C}$ of colors. Consider the smallest set of expressions which contain*

- *the constants from $\mathbb{R}$,*
- *for any index symbol $\nu$ variables $x_\nu, y_\nu, z_\nu$ (referring to $x, y, z$–coordinates of points*

*indexed with $\nu$),*

- *for any two expressions also their sum and product,*
- *for any expression $E$ and index symbol $\nu$ and color $\gamma \in \mathcal{C}$ also $\sum_{\nu \in S_\gamma} E$.*

*The scalar polynomial point set descriptors (PPSDs) for finite point sets with colors in $\mathcal{C}$ are such expressions in which each occurring index symbol $\nu$ is bound unambiguously to some preceding summation sign.*
*For a finite dimensional vector space $V$ the polynomial point set descriptors with values in $V$ are functions from finite point sets with colors in $\mathcal{C}$ to $V$, such that when followed by any linear map $V \to \mathbb{R}$, we get a scalar PPSD.*

Here is an example for a (scalar) polynomial point set descriptor (PPSD) for finite point sets $S_1, S_2 \subset \mathbb{R}^3$ of two colors:

$$\mathcal{D}(S_1, S_2) := \sum_{(x_\kappa, y_\kappa, z_\kappa) \in S_1} \left( \left( \sum_{(x_\nu, y_\nu, z_\nu) \in S_2} x_\nu x_\kappa \right) \cdot \left( \sum_{(x_\nu, y_\nu, z_\nu) \in S_2} (y_\nu - 2 \cdot z_\kappa)^2 \right) \right)$$

Most of the time, we just say PPSD for scalar PPSD. But sometimes (e.g. when embedding a space of configurations into a $\mathbb{R}^d$) we also use vector valued PPSDs. When the values are in some $\mathbb{R}^d$, these are just given by $d$ scalar PPSDs.
In the above example, by expanding all products and moving all summations to the left of the resulting monomials, we can write any such expressions as a linear combination of sums of monomials. For example, the last such term in the expansion for the above descriptor would be

$$\mathcal{D}(S_1, S_2) = \ldots + 4 \cdot \sum_{(x_\kappa, y_\kappa, z_\kappa) \in S_1} \sum_{(x_{\nu_1}, y_{\nu_1}, z_{\nu_1}) \in S_2} \sum_{(x_{\nu_2}, y_{\nu_2}, z_{\nu_2}) \in S_2} x_{\nu_1} x_\kappa \cdot z_\kappa^2$$

(We used $\nu_1$ and $\nu_2$ to differentiate the two sums that both used index $\nu$ above.) In this example we have an "empty" sum: We have to sum over indices $\nu_2$, but do not use any of the coordinates $x_{\nu_2}, y_{\nu_2}, z_{\nu_2}$ in this term (or equivalently, their exponents are all 0). This means

we can rewrite it as

$$\mathcal{D}(S_1, S_2) = \ldots + 4 \cdot |S_2| \cdot \sum_{(x_\kappa, y_\kappa, z_\kappa) \in S_1} \sum_{(x_{\nu_1}, y_{\nu_1}, z_{\nu_1}) \in S_2} x_{\nu_1} x_\kappa \cdot z_\kappa^2$$

We will do this for all empty sums, and then we collect those sums over monomials that sum over the same sets of points, i.e. over $m_1$ points in $S_1$, ..., $m_c$ points in $S_c$, so the monomials depend on $m_1 + \ldots + m_n$ vectors.

This way we get for any PPSD a representation as sum of expressions

$$\begin{aligned} &\mathcal{E}_{m_1,\ldots,m_c;e_1,\ldots,e_c}(S_1, \ldots, S_c) \\ &= |S_1|^{e_1} \cdot \ldots \cdot |S_c|^{e_c} \cdot \sum_{(\boldsymbol{r}_{1,1},\ldots,\boldsymbol{r}_{1,m_1}) \in S_1^{m_1}} \cdots \sum_{(\boldsymbol{r}_{c,1},\ldots,\boldsymbol{r}_{c,m_c}) \in S_c^{m_c}} P_{m_1,\ldots,m_c;e_1,\ldots,e_c}\big(\boldsymbol{r}_{1,1}, \ldots, \boldsymbol{r}_{c,m_c}\big) \quad (7) \end{aligned}$$

where $P_{m_1,\ldots,m_c;e_1,\ldots,e_c}\big(\boldsymbol{r}_{1,1}, \ldots, \boldsymbol{r}_{c,m_c}\big)$ is a polynomial which depends on the vector variables $\boldsymbol{r}_{1,1}, \ldots, \boldsymbol{r}_{c,m_c}$ and in each monomial each of these vectors appears, or equivalently: This polynomial vanishes whenever we substitute one of its input vectors with zero.

(This sum may include terms for $m_1 = \ldots = m_c = 0$, in which case we just have

$$\mathcal{E}_{0,\ldots,0;e_1,\ldots,e_c}(S_1, \ldots, S_c) = |S_1|^{e_1} \cdot \ldots \cdot |S_c|^{e_c} \cdot P_{0,\ldots,0;e_1,\ldots,e_c}$$

with constants $P_{0,\ldots,0;e_1,\ldots,e_c}$.)

Since the order of the $m_i$ vector variables $\boldsymbol{r}_{i,1}, \ldots, \boldsymbol{r}_{i,m_i}$ is arbitrary, we can replace the polynomials by their symmetrization, i.e. we can write this expression always with polynomials that are invariant under the product of symmetric groups $\Sigma_{m_1} \times \Sigma_{m_2} \times \ldots \times \Sigma_{m_c}$ in the sense that

$$\begin{aligned} &P_{m_1,\ldots,m_c;e_1,\ldots,e_c}\big(\boldsymbol{r}_{1,1}, \ldots, \boldsymbol{r}_{1,m_1},\ \ldots,\ \boldsymbol{r}_{c,1}, \ldots \boldsymbol{r}_{c,m_c}\big) \\ &= P_{m_1,\ldots,m_c;e_1,\ldots,e_c}\big(\boldsymbol{r}_{1,\sigma_1(1)}, \ldots, \boldsymbol{r}_{1,\sigma_1(m_1)},\ \ldots,\ \boldsymbol{r}_{c,\sigma_c(1)}, \ldots \boldsymbol{r}_{c,\sigma_c(m_c)}\big) \end{aligned}$$

for any permutations $\sigma_1 \in \Sigma_{m_1}, ...\sigma_c \in \Sigma_{m_c}$. (If the polynomial had the property that it vanished whenever we substitute one of the vector inputs with 0, the symmetrization will also have this property.)

We will call the resulting expression the "Normal Form". (The definite article is appropriate: In the next section we will prove its uniqueness, i.e. the contribution for each $(m_1, ..., m_c; e_1, ..., e_c)$ in a Normal Form can be reconstructed already from the values of a PPSD. This will be used in the proof of Theorem 4.)

If we separate out all monomials in the polynomials $P_{m_1,...,m_c;e_1,...,e_c}$, we can express each PPSD as a sum of expressions

$$\sum_{(x_{\nu_1}, y_{\nu_1}, z_{\nu_1}) \in S_{\gamma_1}} \cdots \sum_{(x_{\nu_k}, y_{\nu_k}, z_{\nu_k}) \in S_{\gamma_k}} x_{\nu_1}^{a_1} \cdot \ldots \cdot x_{\nu_k}^{a_k} \cdot y_{\nu_1}^{b_1} \cdot \ldots \cdot y_{\nu_k}^{b_k} \cdot z_{\nu_1}^{c_1} \cdot \ldots \cdot z_{\nu_k}^{c_k}$$

with $\gamma_1, ..., \gamma_k$ some sequence of colors, which can also be written as

$$\left( \sum_{(x_{\nu_1}, y_{\nu_1}, z_{\nu_1}) \in S_{\gamma_1}} x_{\nu_1}^{a_1} \cdot y_{\nu_1}^{b_1} \cdot z_{\nu_1}^{c_1} \right) \cdot \ldots \cdot \left( \sum_{(x_{\nu_k}, y_{\nu_k}, z_{\nu_k}) \in S_{\gamma_k}} x_{\nu_k}^{a_k} \cdot y_{\nu_k}^{b_k} \cdot z_{\nu_k}^{c_k} \right)$$

which shows that we can evaluate each PPSD as a polynomial in fundamental features (i.e. polynomial features with only one summation).

## H.2 PPSDs on multisets, uniqueness of Normal Form Decomposition

We can identify sets of points $S \subseteq \mathbb{R}^d$ with functions $\mu : \mathbb{R}^d \to \{0, 1\}$ given by

$$\mu(x) := \begin{cases} 1 & \text{if } x \in S \\ 0 & \text{else} \end{cases}$$

By a "multiset" we then mean functions $\mu : \mathbb{R}^d \to \{0, 1, 2, ...\}$ – we can interpret $\mu(s)$ as the multiplicity with which $s$ occurs in $S$. We are here only interested in finite multisets, i.e. there are only finitely many $x$ with $\mu(x) > 0$.

There is a natural extension of a PPSD $\mathcal{D}(S_1, ..., S_c)$ to multisets $S_1, ..., S_c$: If a point $\boldsymbol{r}$ in one of the sets has multiplicity $\mu(\boldsymbol{r}) > 1$, replace it by $m := \mu(\boldsymbol{r})$ points at locations $\boldsymbol{r} + 1\epsilon \cdot \boldsymbol{d}$, $\boldsymbol{r} + 2\epsilon \cdot \boldsymbol{d}$,..., $\boldsymbol{r} + m\epsilon \cdot \boldsymbol{d}$ for some vector $\boldsymbol{d}$ and $\epsilon > 0$. When we let $\epsilon \to 0$, this will let $\mathcal{D}(S_1, ..., S_c)$ converge to what we consider the value for multisets.

We can write down explicitly the value to which this converges: If the PPSD $\mathcal{D}$ is given in Normal Form, we have to interpret the $|S_i|$ in the sense of multisets, i.e. sum up all multiplicities, and we have to multiply the polynomials $P_{m_1,...,m_c;e_1,...,e_c}(\boldsymbol{r}_{1,1}, ..., \boldsymbol{r}_{c,m_c})$ with the additional factor $\mu(\boldsymbol{r}_{1,1}) \cdot ... \cdot \mu(\boldsymbol{r}_{c,m_c})$.

For a set or multiset $S$ given by multiplicities $\mu$ and an integer $a \geq 0$ we write $a\#S$ for the multiset with multiplicities $a \cdot \mu$.

Now we can consider for given PPSD $\mathcal{D}$ and given finite sets $S_1, ..., S_c \subset \mathbb{R}^d$ the values

$$f(a_1, ..., a_c; b_1, ..., b_c) := \mathcal{D}(S'_1, ..., S'_c)$$

with

$$S'_i := \big((a_i - 1) \cdot b_i \cdot |S_i|\big)\#\{0\} \cup \big(b_i\#S_i\big)$$

These new multisets have $|S'_i| = a_i \cdot b_i \cdot |S_i|$ elements and the original elements in $|S_i|$ are repeated $b_i$ times. For each $(m_1, ..., m_c; e_1, ..., e_c)$ occurring in the Normal Form, this gives the extra factor $(a_i b_i)^{e_i}$ from the change in $|S_i|$ and the extra factor $b_i^{m_i}$ from the larger number of terms in the summation (the we only have to consider the nonzero elements of $S'_i$, since the polynomials are constructed such that they are 0 if one of the entries is 0). So as a function of $(a_1, ..., a_c; b_1, ..., b_c)$ this is a polynomial, which is determined by $\mathcal{D}$ and the sets $S_1, ..., S_c$, and since a polynomial determines its coefficients, this means that also the decomposition of $\mathcal{D}(S_1, ..., S_c)$ into individual contributions of each order $(m_1, ..., m_c)$ is

determined by $\mathcal{D}$.

This proves the first part of:

**Lemma 9.**

1. *Any PPSD* $\mathcal{D}(S_1, ..., S_c)$ *has a* unique *decomposition of the Normal Form into contributions* $\mathcal{E}_{m_1,...,m_c;e_1,...,e_c}(S_1, ..., S_c)$.

2. *If* $B$ *is an integer larger than all of the* $m_i$ *and* $e_i$ *in any non–zero contribution* $\mathcal{E}_{m_1,...,m_c;e_1,...,e_c}(S_1, ..., S_c)$, *then the knowledge of all values* $\mathcal{D}(S'_1, ..., S'_c)$ *for* $|S'_i| \leq B^2|S_i|$ *determines the values of the contributions* $\mathcal{E}_{m_1,...,m_c;e_1,...,e_c}(S_1, ..., S_c)$.

*Proof.* The second claim is actually an explicit version of the first part (if there were two different decompositions, take $B$ large enough for both of them, then a contradiction would follow from the second claim). To prove the second claim, we will use an explicit version of the above argument "a polynomial determines its coefficients", based on the Vandermonde matrix: For $m+1$ different numbers $x_0, x_1, ..., x_m$, the Vandermonde matrix $V$ is the $(m+1) \times (m+1)$–matrix defined by the entries $x_i^j$ for $0 \leq i, j \leq m$. Multiplying $V$ with the coefficient vector $(c_0, ..., c_m)$ of a polynomial $p(T) := c_0 + c_1T + ... + c_mT^m$ gives the vector of values $p(x_0), ..., p(x_m)$. The determinant of $V$ is $\prod_{0 \leq i < j \leq m}(x_i - x_j)$, so $V$ is invertible. As a consequence we get the well–known fact that a polynomial of degree $m$ is determined by its values at $m$ different points. Iterating this construction $k$ times, we get that a polynomial $p(T_1, ..., T_k)$ of degrees $\leq m$ in each variable is determined by its $(m+1)^k$ values $p(t_1, ..., t_k)$ for $t_i$ in some set $\{x_0, ..., x_m\}$ of $(m+1)$ different values. Applying this for $k = 2c$ and $\{x_0, ..., x_m\} = \{1, 2, ..., m+1\}$ to the polynomial $f(a_1, ..., a_c; b_1, ..., b_c)$ above then gives the explicit claim 2. □

For the full uniqueness of the normal form, we also have to prove that the functions $\mathcal{D}(S_1, ..., S_c)$ on sets also determine uniquely the corresponding polynomials of vectors; this will be an application of the results of the next section (see H.3.3).

## H.3 Sum over non–degenerate simplices

In the following we will go back to only evaluating the PPSDs at ordinary sets (not multisets).

We will now consider two variants of the summation used in our PPSDs. In particular, our sums over $k$ points always involve all possible combinations of $k$ points, including using the same point several times. We can ask whether we would get a different class of functions if our summations would only sum over $k$–tuples of *different* points, i.e. we would sum over non–degenerate $k$–simplices. In a sense to be made precise, this gives the same class of functions, but the fact that it does is interesting and useful. We will use this for relating our set functions to permutation–invariant polynomials on vectors, and again later for a finiteness result.

To avoid excessively cumbersome notation, we first discuss the case of only one color, and then in the second subsection we detail the (rather straightforward) modifications needed for the general case.

### H.3.1 The case of one color

For one color, the PPSDs in their normal form (7) are sums of expressions

$$\mathcal{E}_{m,e}(S) := |S|^e \cdot \sum_{(\boldsymbol{r}_1,\dots,\boldsymbol{r}_m)\in S^m} P_{m,e}(\boldsymbol{r}_1,\dots,\boldsymbol{r}_m)$$

where $P_{m,e}$ is a polynomial of $m$ vector inputs.

For $P$ a polynomial of $m$ vector inputs, we will consider the sums

$$\mathcal{E}_P(S) := \sum_{(\boldsymbol{r}_1,\dots,\boldsymbol{r}_m)\in S^m} P(\boldsymbol{r}_1,\dots,\boldsymbol{r}_m) = \sum_{\alpha:[m]\to S} P(\alpha(1),\dots,\alpha(m))$$

where in the second formulation the sum is over all maps from $[m] := \{1,2,\dots,m\}$ to $S$.

We will now introduce the variants in which we restrict $\alpha$ to only injective or to only surjective maps. This means in the sum each point is only allowed to be used once ($\alpha$ injective) or

each point has to be used ($\alpha$ surjective):

$$\mathcal{E}_P^\circ(S) \;:=\; \sum_{\alpha:[m]\rightarrowtail S} P\Big(\alpha(1),...,\alpha(m)\Big)$$
$$\mathcal{E}_P^*(S) \;:=\; \sum_{\alpha:[m]\twoheadrightarrow S} P\Big(\alpha(1),...,\alpha(m)\Big)$$

To avoid having to exclude the special case $S = \varnothing$, define $\mathcal{E}_P(\varnothing) = \mathcal{E}_P^\circ(\varnothing) = \mathcal{E}_P^*(\varnothing) = 0$.

A map $\alpha : [m] \to S$ can only be injective for $|S| \geq m$, can only be surjective for $|S| \leq m$, and for $|S| = m$ it is injective if and only if it surjective, so we have

$$\mathcal{E}^\circ(S) = 0 \qquad \text{for } |S| < m, \tag{8}$$
$$\mathcal{E}^\circ(S) = \mathcal{E}^*(S) \qquad \text{for } |S| = m, \tag{9}$$
$$0 = \mathcal{E}^*(S) \qquad \text{for } |S| > m. \tag{10}$$

By binning all maps $\alpha : [m] \to S$ by their image, we get from the definition:

$$\mathcal{E}_P(S) = \sum_{T\subseteq S}\sum_{\alpha:[m]\twoheadrightarrow T} P\Big(\alpha(1),...,\alpha(m)\Big) = \sum_{T\subseteq S}\mathcal{E}_P^*(T) \tag{11}$$

This leads to the following conclusion:

**Lemma 10.** *Knowing $\mathcal{E}_P(T)$ for all $T \subseteq S$ is equivalent to knowing $\mathcal{E}_P^*(T)$ for all $T \subseteq S$, these lists of numbers are transformed into each other by a linear transformation with integer coefficients that only depends on $|S|$.*

*Proof.* This is obvious from (11) for the direction $\mathcal{E}^* \to \mathcal{E}$, but if we order the subsets $T \subseteq S$ by the number of their elements, (11) describes a linear transformation in triangular form with 1 on the diagonal, so it is also reversible with 1 on the diagonal. □

We can make analogous arguments for $\mathcal{E}^\circ$: We can bin all maps $\alpha : [m] \to S$ by the equivalence

relation $\sim$ on $[m]$ underlying $\alpha$ given by

$$i \sim j \;:\Leftrightarrow\; \alpha(i) = \alpha(j)$$

Let $Eq(m)$ be the set of all equivalence relations on $[m]$, and for $\sim\, \in Eq(m)$ let $|\sim|$ denote the number of equivalence classes in $\sim$.

Let $\sim\, \in Eq(m)$ be an equivalence relation with $k := |\sim|$ classes. Then there is a function $\beta : [m] \twoheadrightarrow [k]$ that is a bijection on $[m]/\sim$, ie. enumerates the $k$ equivalence classes. For example, we can take $\beta$ to be the enumeration in order of ascending first element.

`Enumeration of equivalence classes` $\beta$

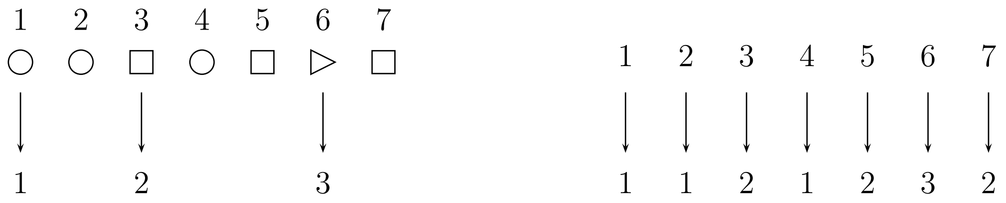


With such a $\beta$ (a fixed choice for each $\sim$), the equation $\alpha(i) = \gamma(\beta(i))$ defines a one–to–one relationship from the set of maps $\gamma : [k] \to S$ to the set of maps $\alpha : [m] \to S$ with the equivalence relation $\sim$.

For a polynomial $P$ of $m$ inputs we can define a polynomial of $k$ inputs as $P(\boldsymbol{r}_{\beta(1)}, ..., \boldsymbol{r}_{\beta(m)})$. Since we will sum this over $k$–tuples, we will normalize it to be symmetric under $\Sigma_k$, this does not change the sum over $k$–tuples, but keeps us in the space of symmetric polynomials. This leads us to the definition

$$P/\sim(\boldsymbol{r}_1, ..., \boldsymbol{r}_k) := \frac{1}{k!} \sum_{\sigma \in \Sigma_k} P(\boldsymbol{r}_{\sigma\beta(1)}, ..., \boldsymbol{r}_{\sigma\beta(m)})$$

(With this definition, we also get the same $P/\sim$ independent of our choice of $\beta$: The maps $\sigma\beta$ are just all bijections $[m]/\sim\, \to [k]$.)

With these notations, binning maps $\alpha : [m] \to S$ by their underlying equivalence relation

gives

$$\mathcal{E}_P(S) = \sum_{\sim\in Eq(m)} \sum_{\gamma:[|\sim|]\rightarrowtail S} P/\sim(\gamma(1),\dots,\gamma(k)) = \sum_{\sim\in Eq(m)} \mathcal{E}^{\circ}_{P/\sim}(S) \tag{12}$$

For $\mathcal{E}^*$ we considered the $T \subseteq S$ partially ordered by $\subseteq$ and with largest element the original set $S$; here we here have the $\sim\in Eq(m)$ partially ordered by refinement and with finest equivalence relation the equality, with $P/=$ giving the original polynomial $P$.

So in the same way as Lemma 10, we here get the conclusion:

**Lemma 11.** *Given a polynomial $P$ in $m$ vector variables, knowing $\mathcal{E}_{P/\sim}(S)$ for all $\sim\in Eq(m)$ is equivalent to knowing $\mathcal{E}^{\circ}_{P/\sim}(S)$ for all $\sim\in Eq(m)$, these (finite) lists of numbers are transformed into each other by a linear transformation with integer coefficients that only depends on $m$.*

As an example, for a polynomial $P$ of three variables we have:

$$\mathcal{E}_P(S) = \mathcal{E}^{\circ}_P(S) + \mathcal{E}^{\circ}_{P_{112}}(S) + \mathcal{E}^{\circ}_{P_{121}}(S) + \mathcal{E}^{\circ}_{P_{122}}(S) + \mathcal{E}^{\circ}_{P_{111}}(S)$$

where we write $P_{abc}$ for the $P/\sim$ with the $\sim$ corresponding to $\beta(1)=a$, $\beta(2)=b$, $\beta(3)=c$, so in particular $P_{112}(\boldsymbol{r}_1,\boldsymbol{r}_2) = P(\boldsymbol{r}_1,\boldsymbol{r}_1,\boldsymbol{r}_2)$, $P_{111}(\boldsymbol{r}) = P(\boldsymbol{r},\boldsymbol{r},\boldsymbol{r})$ and $P_{123} = P$.

We can now use that $\mathcal{E}^{\circ}_P(S) = 0$ for $|S| < m$:
In our example, for $|S| < 2$ we have $\mathcal{E}^{\circ}_P(S) = 0$, so

$$|S| < 3 \quad \Rightarrow \quad \mathcal{E}_P(S) = \mathcal{E}^{\circ}_{P_{112}}(S) + \mathcal{E}^{\circ}_{P_{121}}(S) + \mathcal{E}^{\circ}_{P_{122}}(S) + \mathcal{E}^{\circ}_{P_{111}}(S).$$

Expressing now $\mathcal{E}^{\circ}_Q$ again in terms of $\mathcal{E}_Q$, we get:

$$|S| < 3 \quad \Rightarrow \quad \mathcal{E}_P(S) = \mathcal{E}_{P_{112}}(S) + \mathcal{E}_{P_{121}}(S) + \mathcal{E}_{P_{122}}(S) - 2\cdot\mathcal{E}_{P_{111}}(S).$$

In general we get:

**Lemma 12.** *For $n \geq 1$ and any $\mathcal{E}_P(S)$ we get a linear combination of $\mathcal{E}_Q(S)$ with each $Q$ having at most $n$ arguments, such that both expressions are the same for all sets $S$ with $|S| \leq n$. If $\mathcal{E}_P$ can be written as a polynomial of fundamental features $f_1, ..., f_m$, then also the $\mathcal{E}_Q$ can be written as polynomials of $f_1, ..., f_m$.*

*Proof.* Use Lemma 11 to write $\mathcal{E}_P(S)$ as a sum of $\mathcal{E}^\circ_Q(S)$; in this sum we can discard any $Q$ with more than $n$ arguments by (8); then use Lemma 11 again to write the remaining $\mathcal{E}^\circ_Q$ in terms of $\mathcal{E}_R$. □

### H.3.2 The general case (several colors)

In general, we will have the polynomial $P$ depend on $m_1 + m_2 + ... + m_c$ vectors (corresponding to $m_1$ points of color 1, $m_2$ points of color 2 etc.) and it will be symmetric under the permutation group $\Sigma_{m_1} \times ... \times \Sigma_{m_c}$. We then define

$$\mathcal{E}_P(S_1, ..., S_c) := \sum_{\alpha_1:[m_1]\to S_1} \cdots \sum_{\alpha_c:[m_c]\to S_c} P\Big(\alpha_1(1), ..., \alpha_1(m_1);\ ...\ ;\ \alpha_c(1), ...\alpha_c(m_c)\Big)$$

and its variants

$$\begin{aligned}
\mathcal{E}^\circ_P(S_1, ..., S_c) &:= \sum_{\alpha_1:[m_1]\rightarrowtail S_1} \cdots \sum_{\alpha_c:[m_c]\rightarrowtail S_c} P\Big(\alpha_1(1), ..., \alpha_1(m_1);\ ...\ ;\ \alpha_c(1), ...\alpha_c(m_c)\Big) \\
\mathcal{E}^*_P(S_1, ..., S_c) &:= \sum_{\alpha_1:[m_1]\twoheadrightarrow S_1} \cdots \sum_{\alpha_c:[m_c]\twoheadrightarrow S_c} P\Big(\alpha_1(1), ..., \alpha_1(m_1);\ ...\ ;\ \alpha_c(1), ...\alpha_c(m_c)\Big)
\end{aligned}$$

We set all these values to zero if at least one of the sets $S_1, ..., S_c$ is empty. We have $\mathcal{E}^\circ_P(S_1, ..., S_c) = 0$ when there is a $|S_i| < m_i$, $\mathcal{E}^*_P(S_1, ..., S_c) = 0$ when there is a $|S_i| > m_i$, and $\mathcal{E}^\circ_P(S_1, ..., S_c) = \mathcal{E}^*_P(S_1, ..., S_c)$ when all $|S_i| = m_i$.

For $\mathcal{E}^*$ we bin the $c$–tuples $(\alpha_1, ..., \alpha_c)$ by the $c$–tuples of their images $(T_1, ..., T_c)$, these tuples are a partially ordered set with largest element $(S_1, ..., S_c)$ and we get the general form of Lemma 10:

**Lemma 13.** *Knowing $\mathcal{E}_P(T_1, ..., T_c)$ for all $T_i \subseteq S_i$ is equivalent to knowing $\mathcal{E}^*_P(T_1, ..., T_c)$ for all $T_i \subseteq S_i$, these lists of numbers are transformed into each other by a linear transformation with integer coefficients that only depends on $|S_1|, ..., |S_c|$.*

For $\mathcal{E}^\circ$ we consider the set $Eq(m_1, ..., m_c) = Eq(m_1) \times ... \times Eq(m_c)$ of $c$–tuples of equivalence relations on $[m_1]$, ..., $[m_c]$. Again this set is partially ordered by refinement, and the finest equivalence is the identity of each of $[m_1]$, ..., $[m_c]$. For $\sim = (\sim_1, ..., \sim_c) \in Eq(m_1, ..., m_c)$ write $|\sim| := (|\sim_1|, ..., |\sim_c|)$. If $|\sim| = (k_1, ..., k_c)$, the equivalence relations can be given by enumerations of equivalence classes $\beta_i : [m_i] \to [k_i]$ as above, and we can define as above

$$P/\sim(\boldsymbol{r}_{1,1}, ..., \boldsymbol{r}_{1,k_1}; ...; \boldsymbol{r}_{c,1}, ..., \boldsymbol{r}_{c,k_c}) :=$$
$$\frac{1}{k_1! \cdot ... \cdot k_c!} \sum_{\sigma \in \Sigma_{k_1}} ... \sum_{\sigma \in \Sigma_{k_c}} P(\boldsymbol{r}_{1,\sigma_1\beta_1(1)}, ..., \boldsymbol{r}_{1,\sigma_1\beta_1(m_1)}; ...; \boldsymbol{r}_{c,\sigma_c\beta_c(1)}, ..., \boldsymbol{r}_{c,\sigma_c\beta_c(m_c)})$$

With this definition we get the general version of Lemma 11:

**Lemma 14.** *Given a polynomial $P$ in $m_1 + ... + m_c$ vector variables, knowing $\mathcal{E}_{P/\sim}(S_1, ..., S_c)$ for all $\sim \in Eq(m_1, ..., m_c)$ is equivalent to knowing $\mathcal{E}^\circ_{P/\sim}(S_1, ..., S_c)$ for all $\sim \in Eq(m_1, ..., m_c)$, these (finite) lists of numbers are transformed into each other by a linear transformation with integer coefficients that only depends on $m$.*

This gives in the same way as for one color the general version of Lemma 12:

**Lemma 15.** *For $n_1, ..., n_c \geq 1$ and any $\mathcal{E}_P(S_1, ..., S_c)$ we get a linear combination of $\mathcal{E}_Q(S_1, ..., S_c)$ with each $Q$ having at most $n_1, ..., n_c$ arguments for vectors in $S_1, ..., S_c$, such that both expressions are the same for all sets $S_1, ..., S_c$ with $|S_1| \leq n_1, ..., |S_c| \leq n_c$.*

### H.3.3 Application: Uniqueness of normal form

**Lemma 16.**

1. *The Normal Form (given by the polynomials $P_{m_1,...,m_c;e_1,...,e_c}$) of a PPSD $\mathcal{D}$ is unique.*

2. *If the integer $B$ is larger than all $m_i$ and $e_i$ occurring in a non–zero contribution, then these polynomials are determined by the values of the $\mathcal{D}(S_1, ..., S_c)$ with $|S_1| = ... = |S_c| = B^3$.*

*Proof.* By the second part of Lemma 9, the knowledge of $\mathcal{D}(S_1, ..., S_c)$ with $|S_1| = ... = |S_c| = B^3$ determines the contributions $\mathcal{E}_{m_1,...,m_c;e_1,...,e_c}(S_1, ..., S_c)$ for $|S_1| = ... = |S_c| = B$. So it is enough to look at an individual contribution $\mathcal{E}_{m_1,...,m_c;e_1,...,e_c}$ of which we know the values for $|S_1| = ... = |S_c| = B$. First note that the $e_1, ..., e_c$ already give all information about the factor $|S_1|^{e_1} \cdot ... \cdot |S_c|^{e_c}$, so without limitation of generality we can restrict the proof to the case $e_1 = ... = e_c = 0$. Furthermore, by the way our polynomials $P_{m_1,...,m_c;0,...,0}$ are specified in the normal form, the value of $\mathcal{E}_{m_1,...,m_c;0,...,0}(T_1, ..., T_c)$ is not changed if we add additional points $P = (0, 0, ..., 0)$ at the origin to multisets $T_1, ..., T_c$. This means the knowledge of all values of $\mathcal{E}_{m_1,...,m_c;0,...,0}(S_1, ..., S_c)$ for $|S_1| = B, ..., |S_c| = B$ includes also the knowledge of $\mathcal{E}_{m_1,...,m_c;0,...,0}(T_1, ..., T_c)$ for $T_1 \subseteq S_1, ..., T_c \subseteq S_c$, so by Lemma 13 this also determines the values $\mathcal{E}^*_{m_1,...,m_c;0,...,0}(T_1, ..., T_c)$ for $|T_1| = m_1$,...,$|T_c| = m_c$ and thus the polynomial

$$
\begin{aligned}
& P_{m_1,...,m_c}(\boldsymbol{r}_{1,1}, ..., \boldsymbol{r}_{1,m_1}\, ; \, ... \, ; \boldsymbol{r}_{c,1}, ..., \boldsymbol{r}_{c,m_c}) \\
= \; & \frac{1}{m_1! \cdot ... \cdot m_c!} \sum_{\alpha_1:[m_1] \twoheadrightarrow T_1} \cdots \sum_{\alpha_c:[m_c] \twoheadrightarrow T_c} P\Big(\alpha_1(1), ..., \alpha_1(m_1); \; ... \; ; \; \alpha_c(1), ...\alpha_c(m_c)\Big) \\
= \; & \frac{1}{m_1! \cdot ... \cdot m_c!} \cdot \mathcal{E}^*_P(S_1, ..., S_c).
\end{aligned}
$$

is also determined. □

## H.4 Comparison of PPSDs with functions of configurations of fixed size

So far we have only looked at configuration features (PPSDs) that are defined for configurations of any size. One may ask how our notion of PPSDs differs from just giving different polynomials for point sets for different fixed numbers of points. Obviously for any PPSD

$\mathcal{D}(S_1, ..., S_c)$ and given numbers of points $m_1, ..., m_c$ of each color, we can write down the function

$$P(\boldsymbol{r}_{1,1}, ..., \boldsymbol{r}_{1,m_1}\,; \,...\, ;\, \boldsymbol{r}_{c,1}, ..., \boldsymbol{r}_{c,m_c}) := \mathcal{D}\big(\{\boldsymbol{r}_{1,1}, ..., \boldsymbol{r}_{1,m_1}\}, ..., \{\boldsymbol{r}_{c,1}, ..., \boldsymbol{r}_{c,m_c}\}\big)$$

and it will be a polynomial that is invariant under $\Sigma_{m_1} \times ... \times \Sigma_{m_c}$. We now show to what extent we can also go back from polynomials of a fixed number of arguments to PPSDs:

**Proposition 17.**

1. *For any given numbers of points $m_1, ..., m_c$ of each color, any polynomial function $P$ on configurations of $|S_1| = m_1, ..., |S_c| = m_c$ points of color $1, ..., c$ can be obtained by restricting a PPSD to these configurations. (We assume here that the given polynomial function $P$ in $m_1 + ... + m_c$ vector arguments is indeed a function on sets, i.e. is invariant with respect to permuting arguments corresponding to the same color.)*

2. *For any* finite set *of data $(m_1, ..., m_c, P)$ as in 1, there is a PPSD that gives these functions on the corresponding configurations.*

3. *Any PPSD is given by the values on point sets of a sufficiently large fixed size (the $B^3$ of Lemma 16).*

To rephrase this proposition: We can give separate functions on some types of configurations, and always can complete it to a general PPSD on all configurations. However, this can only be done for a finite set of configurations – from some size on (which we can make arbitrarily large), the function on larger configurations must follow a general pattern, which determines the values on all large configurations by the values on the configurations at some particular size.

The third part is just pointing to Lemma 16, we now give the proofs of first two parts.

*Proposition 17, 1.*

A polynomial function on configurations of $m_1, ..., m_c$ points of color $1, ..., c$ is given directly

by a polynomial $P(\boldsymbol{r}_{1,1},...,\boldsymbol{r}_{1,m_1}\,;\,...\,;\,\boldsymbol{r}_{c,1},...,\boldsymbol{r}_{c,m_c})$ that is invariant under $\Sigma_{m_1}\times...\times\Sigma_{m_c}$. For PPSDs we have to transform this into sums over points from the sets $S_1 := \{\boldsymbol{r}_{1,1},...,\boldsymbol{r}_{1,m_1}\}$, ..., $S_c := \{\boldsymbol{r}_{c,1},...,\boldsymbol{r}_{c,m_c}\}$. This is easy for the version $\mathcal{E}^\circ$:

$$
\begin{aligned}
&P(\boldsymbol{r}_{1,1},...,\boldsymbol{r}_{1,m_1}\,;\,...\,;\,\boldsymbol{r}_{c,1},...,\boldsymbol{r}_{c,m_c})\\
&= \frac{1}{m_1!\cdot...\cdot m_c!}\sum_{\alpha_1:[m_1]\rightarrowtail S_1}\cdots\sum_{\alpha_c:[m_c]\rightarrowtail S_c} P\Big(\alpha_1(1),...,\alpha_1(m_1);\ ...\ ;\ \alpha_c(1),...\alpha_c(m_c)\Big)\\
&= \frac{1}{m_1!\cdot...\cdot m_c!}\cdot\mathcal{E}^\circ_P(S_1,...,S_c).
\end{aligned}
$$

According to Lemma 14 we can translate this into a linear combination of $\mathcal{E}_Q(S_1,...,S_c)$ for polynomials $Q = P/\sim$, this is then a PPSD. □

*Proposition 17, 2.* For any finite list of different $c$–tupels of integers $(k_{i,1},...,k_{i,c})$ we can write down polynomials that are 0 for all of the (finitely many) given $c$–tupels except for the one with index $i$, e.g.

$$\prod_{j\neq i}\Big((k_1-k_{j,1})^2+...+(k_c-k_{j,c})^2\Big)$$

Let $Q_i(k_1,...,k_c)$ be this polynomial, divided by its value at $(k_{i,1},...,k_{i,c})$, so at the given $c$–tuples it has only values 0 with one exception at tuple $i$, where it is 1.

We get the numbers $|S_i|$ as the value of the PPSD $\sum_{\boldsymbol{r}\in S_i}1$. So the expressions $Q_i(|S_1|,...,|S_c|)$ are also PPSDs.

Now we can construct for all $c$–tuples $(|S_1|,...,|S_c|)$ the PPSDs from part 1, multiply them with with the corresponding $Q_i$, and sum up all these products. This gives a PPSD with the desired functions on each given $c$–tuple $(|S_1|,...,|S_c|)$. □

# I Proof of Theorem 1

## I.1 Fundamental features describe sets of points

Here we prove the first part of theorem 1, i.e. that the fundamental features uniquely describe point sets – this is not yet using the group $G$. (We can see this as giving coordinates to the infinite dimensional variety of finite point sets — polynomial point set descriptors are then just the polynomial functions in these coordinates.)

We have already seen in B.3 that it is enough to prove this in the case 1, i.e. $X = \mathbb{S}^2$ (and polynomial functions). Furthermore, for this first part of Theorem 1 the subsets of points of different color and their fundamental features are completely independent, so we can treat each color separately, and just assume we only have one color.

We will use the following easy lemma:

**Lemma 18.** *Let $S$ be a finite set of points in $\mathbb{R}^d$. Then there is a linear function $L : \mathbb{R}^d \to \mathbb{R}$ such that $L$ is injective on $S$.*

*Proof.* We prove this by induction on $d$. For $d = 1$ there is nothing to prove, so suppose now that $d > 1$ and we know the statement for $d - 1$.
If we have finitely many different points in $S \subset \mathbb{R}^d$, there are only finitely many directions for projections to a $d - 1$ – dimensional space under which two points are mapped to the same point, avoiding them gives a projection $f : \mathbb{R}^d \to \mathbb{R}^{d-1}$ that is injective on $S$. Using the induction hypothesis then gives a linear map $L : \mathbb{R}^{d-1} \to \mathbb{R}$ that is injective on $f(S)$, so together $s \mapsto L(f(s))$ is a linear function $\mathbb{R}^d \to \mathbb{R}$ which is injective on $S$. □

Now we can prove the first part of Theorem 1 in the polynomial cases 1, 2i, 3i; and as seen in B.3 this implies it also for the other cases 2ii, 3ii:

**Lemma 19.** *Let $S \subset \mathbb{R}^3$ be a set of at most $n$ elements. Then the fundamental features $\sum_{\boldsymbol{r} \in S} P(\boldsymbol{r})$ with polynomials $P : \mathbb{R}^3 \to \mathbb{R}$ of degree $\leq n$ uniquely characterize the set $S$.*

*Equivalently, the first $n+1$ moments*

$$\sum_{\boldsymbol{r}\in S} \boldsymbol{r}^{\otimes k} \qquad \textit{for } k = 0, 1, ..., n$$

*determine the set $S$.*

*Proof.* The two versions are equivalent because the entry with index $(e_1, ..., e_k)$ with $1 \le e_i \le 3$ in the moment tensor of order $k$ is just the fundamental feature $\sum_{(x,y,z)\in S} x^a\, y^b\, z^c$ with $a, b, c$ the number of indices $1, 2, 3$ in $(e_1, ..., e_k)$.

We can assume the sets $S$ have exactly $n$ elements, since the number of elements is already given by the 0-th moment.

This lemma is well known in dimension 1: A set $\{x_1, ..., x_n\}$ of $n$ numbers is characterized by the polynomial $(x - x_1) \cdot ... \cdot (x - x_n)$ or equivalently by its coefficients, which are given by the elementary symmetric functions

$$\begin{aligned}
\sigma_1 &:= x_1 + x_2 + ... + x_n \\
\sigma_2 &:= x_1 \cdot x_2 + x_1 \cdot x_3 + ... + x_{n-1} \cdot x_n \\
... & \\
\sigma_m &:= x_1 \cdot ... \cdot x_n,
\end{aligned}$$

and these in turn are given by the first $n$ power sums

$$\begin{aligned}
\tau_1 &:= x_1 + x_2 + ... + x_n \\
\tau_2 &:= x_1^2 + x_2^2 + ... + x_n^2 \\
... & \\
\tau_n &:= x_1^n + x_2^n + ... + x_n^n.
\end{aligned}$$

(A formula for computing the $\sigma_i$ from the $\tau_i$ and vice versa is the Girard-Newton formula, but we do not need the explicit formula).

To deduce the higher dimensional (here: 3-dimensional) case from this, assume we have two different sets $S \neq S'$ of at most $n$ points in $\mathbb{R}^3$ each; we have to show that there is a fundamental feature of degree $\leq n$ that distinguishes these two sets. According to the last lemma, we can find a linear map $L : \mathbb{R}^d \to \mathbb{R}$ that is injective on $S \cup S'$. This means $L(S)$ and $L(S')$ are two different subsets of $\mathbb{R}$, so by the 1-dimensional case, this means that that one of the power sums $\tau_k$ with $0 < k \leq n$ must be different, which means in turn that a fundamental feature

$$\sum_{\boldsymbol{r} \in S} L(\boldsymbol{r})^k \neq \sum_{\boldsymbol{r} \in S'} L(\boldsymbol{r})^k$$

of degree $k \leq n$ distinguishes $S$ and $S'$. □

## I.2 Completeness of invariant polynomial descriptors

We will now use this and the following proposition to prove the second part of theorem 1:

**Proposition 20.** *Let $V$ be an Euclidean vector space with $G$–representation $\rho : G \to O(V)$, and $\boldsymbol{v}, \boldsymbol{w}$ be two points of $V$ that are not related as $\boldsymbol{w} = \rho(g)\boldsymbol{v}$ for any $g \in G$. Then there is a $G$–invariant polynomial $P : V \to \mathbb{R}$ such that $P(\boldsymbol{v}) \neq P(\boldsymbol{w})$.*

*Proof.* We choose an orthonormal basis of $V$ to identify it with some $\mathbb{R}^d$. Since $G$ is compact, the orbits $\rho(G)\boldsymbol{v}$ and $\rho(G)\boldsymbol{w}$ are compact, so they are also closed. By assumption, they are disjoint. Since $V$ is a metric space, it is normal and Urysohn's lemma implies that there is a continuous function that is 0 on the one compact set and 1 on the other. So let $f : V \to \mathbb{R}$ be such a continuous function with $f(\rho(G)\boldsymbol{v}) = \{0\}$ and $f(\rho(G)\boldsymbol{w}) = \{1\}$.

Since the orbits $\rho(G)\boldsymbol{v}$ and $\rho(G)\boldsymbol{w}$ are compact, they are also bounded, so let $R > 0$ be such that both are contained in the closed hypercube $H := [-R, R]^d$. By the Stone–Weierstrass approximation theorem, there is then for any $\epsilon > 0$ also a polynomial function $p : V \to \mathbb{R}$ such

that $|f(\boldsymbol{x}) - p(\boldsymbol{x}) < \epsilon|$ for all $\boldsymbol{x} \in H$. Let $p$ be such a polynomial function for $\epsilon := 0.1$. Then

$$p(\rho(G)\boldsymbol{v}) \subseteq [-0.1, 0.1] \qquad \text{and} \qquad p(\rho(G)\boldsymbol{w}) \subseteq [0.9, 1.1]$$

Averaging over $G$ gives now a function

$$P(\boldsymbol{x}) := \int_{g \in G} p(\rho(g)\boldsymbol{x}) dg \tag{13}$$

for the Haar measure $dg$ on $G$ with total mass $\int_G 1 = 1$. Since for each $g \in G$ the function $\boldsymbol{x} \mapsto p(\rho(g)\boldsymbol{x})$ is a polynomial, the integral (13) can actually be expressed as a polynomial in $\boldsymbol{x}$ with coefficients that are integrals over $G$, so the function $P : V \to \mathbb{R}$ is actually a polynomial. By construction, $P$ is $G$–invariant, and still satisfies the inequalities

$$P(\rho(G)\boldsymbol{v}) \subseteq [-0.1, 0.1] \qquad \text{and} \qquad p(\rho(G)\boldsymbol{w}) \subseteq [0.9, 1.1]$$

so in particular we have $P(\boldsymbol{v}) \neq P(\boldsymbol{w})$. □

We will now apply this proposition not to our original 3–dimensional vector space $\mathbb{R}^3$, but to a vector space which is given by fundamental features. To prove the second part of Theorem 1, assume we have two configurations $\{(\boldsymbol{r}_i, \gamma_i)\}$ and $\{(\boldsymbol{r}'_i, \gamma'_i)\}$ of points in $X \subseteq \mathbb{R}^3$ with colors in $\mathcal{C}$, that are not equivalent under $G$, i.e. for every $g \in G$

$$\{(\boldsymbol{r}_i, \gamma_i)\} \neq \{(g\boldsymbol{r}'_i, \gamma'_i)\}.$$

We can first try to distinguish them by counting the number of points of each color, using the fundamental features $\sum_{\boldsymbol{r} \in S_\gamma} 1 = |S_\gamma|$ for $\gamma \in \mathcal{C}$. If we do not see a difference, we already know that the numbers $|S_\gamma|$ are the same for both configurations.
Now let $\mathcal{X}$ be the set of all configurations consisting of $k_1$ points of color 1, ... $k_c$ points of color $c$, and let $p, q \in \mathcal{X}$ be the two configurations that are not equivalent under $G$. We can

see $\mathcal{X} = (\mathbb{R}^{3k_1}/\Sigma_{k_1}) \times ... \times (\mathbb{R}^{3k_c}/\Sigma_{k_c})$ (with $\Sigma_k$ as the symmetric group permuting $k$ points) as a topological space, and each feature is a continuous map $\mathcal{X} \to \mathbb{R}$.

From the previous section we know that for every $g \in G$ there must be a fundamental feature $f_g : \mathcal{X} \to \mathbb{R}$ with $f_g(p) \neq f_g(gq)$. Since the features are continuous functions, there must also be a open neighborhood $U_g$ of $g$ in $G$ for which $f_g(p) \neq f_g(g'q)$ for all $g' \in U_g$. Since $G$ is compact, finitely many of these open sets $U_g$ must already cover $G$, i.e. there are finitely many fundamental features that, taken together, can distinguish $p$ from $g'q$ for all $g' \in G$.

Let $n$ be the maximal degree of a polynomial occurring in some of these finitely many fundamental features, and in case ii let also $J \subseteq I$ be the finite subset of all $j$ such that the radial basis function $g_j$ occurs in some of these finitely many fundamental features. Let $\mathcal{P}^{(n)}$ be the finite dimensional vector space of polynomials in $x, y, z$ of degree $\leq n$. In case i each $(\gamma, P) \in \mathcal{C} \times \mathcal{P}^{(n)}$ determines a fundamental feature $f_{(\gamma,P)}$ as

$$\sum_{\boldsymbol{r} \in S_\gamma} P(\boldsymbol{r})$$

and in case ii each $(\gamma, P, j) \in \mathcal{C} \times \mathcal{P}^{(n)} \times J$ determines a fundamental feature $f_{(\gamma,P,j)}$ as

$$\sum_{\boldsymbol{r} \in S_\gamma} P\Big(\frac{\boldsymbol{r}}{|\boldsymbol{r}|}\Big) g_j(|\boldsymbol{r}|).$$

The linear combinations of these fundamental features form a finite dimensional vector space $\mathcal{Y}$, it can be described in case i as

$$\mathcal{Y} := \mathbb{R}^{\mathcal{C}} \otimes \mathcal{P}^{(n)},$$

and in case ii as

$$\mathcal{Y} := \mathbb{R}^{\mathcal{C}} \otimes \mathcal{P}^{(n)} \otimes \mathbb{R}^J.$$

(If some $P_m(x, y, z)$ are a basis of $\mathcal{P}^{(n)}$, then the fundamental features described by $\gamma \otimes P_m \otimes j$ with $\gamma \in \mathcal{C}$ and $j \in J$ would be a basis of $\mathcal{Y}$ in case ii.)

We denote the evaluation of a fundamental feature $y \in \mathcal{Y}$ on a configuration $x \in \mathcal{X}$ as $\langle x, y\rangle$, it is a linear function in $y \in \mathcal{Y}$. As a function on $\mathcal{X}$ this is a vector valued PPSD with values in the finite dimensional vector space $\mathcal{Y}^*$: When followed by a basis vector $\gamma \otimes P_m \otimes j \in \mathcal{Y}$ (as a map $\mathcal{Y}^* \to \mathbb{R}$), we get the fundamental feature $f_{\gamma,P_m,j}$ on $\mathcal{X}$.

Furthermore, we have an action of $G$ on $\mathcal{Y}$ which acts trivially on $\mathcal{C}$ and $J$, but maps $P \mapsto gP$ with $gP(\boldsymbol{r}) := P(g^{-1}\boldsymbol{r})$. This action on $\mathcal{Y}$ satisfies

$$\langle gx, gy\rangle = \langle x, y\rangle.$$

We can use this to map a $x \in \mathcal{X}$ to the linear function on $\mathcal{Y}$

$$\iota : \mathcal{X} \to \mathcal{Y}^*, x \mapsto \big(y \mapsto \langle x, y\rangle\big).$$

On $\mathcal{Y}^*$ the group $G$ operates as $gL := \big(y \mapsto L(g^{-1}y)\big)$, this means the map $\iota : \mathcal{X} \to \mathcal{Y}^*$ is covariant:

$$\iota(gx) = (y \mapsto \langle gx, y\rangle) = (y \mapsto \langle x, g^{-1}y\rangle) = g\ \iota(x)$$

Since $G$ is compact, we can choose a $G$–invariant scalar product on $\mathcal{Y}^*$, with respect to this scalar product we have an orthogonal representation $\rho : G \to O(\mathcal{Y}^*)$. Now we can apply Proposition 20 to find a $G$–invariant polynomial in the fundamental features, i.e. a $G$–invariant PPSD, which distinguishes the two given configurations.

## I.3 Describing configurations by finitely many features

For the last part of Theorem 1 we will need a stronger version of this embedding of $\mathcal{X}$ to $\mathcal{Y}^*$ which not only maps the orbits of $p$ and $q$ to disjoint sets, but is injective on compact subsets of $\mathcal{X}$. This is no problem in the case i: Lemma 19 shows that in that case $\iota : \mathcal{X} \to \mathcal{Y}^*$ is already injective. So we only consider the case ii here.

For some fixed $R > 0$ we denote by $B_R$ the closed ball of radius $R$ around the origin in $\mathbb{R}^3$.

We will now give a strengthened version of the argument in B.3.

**Lemma 21.**

*[Assuming the case ii, with Condition $II_w(2k)$]*

*Let $k = k_1 + ... + k_c$, $R > 0$, and $J$ be the finite subset of $I$ from Condition $II'_w(2k)$. Let*

$$\mathcal{X} := \left(B_R^{k_1}/\Sigma_{k_1}\right) \times ... \times \left(B_R^{k_c}/\Sigma_{k_c}\right).$$

*and with $\mathcal{P}^{(2k)}$ the polynomials of degree $\leq 2k$ set*

$$\mathcal{Y} := \mathbb{R}^{\mathcal{C}} \otimes \mathcal{P}^{(2k)} \otimes \mathbb{R}^J.$$

*Then the map*

$$\iota : \mathcal{X} \to \mathcal{Y}^*, x \mapsto \left(y \mapsto \langle x, y \rangle\right).$$

*is injective, covariant, and given by fundamental features.*

*Proof.* We have already seen this is covariant and given by fundamental features. For the injectivity, assume we are given two configurations of $k$ points. Then at most $2k$ radii occur in them, so let's say all radii appear in the list $0 \leq t_1 < t_2 < ... < t_{2k}$. Then we can find linear combinations $f_a$ for $a = 1, 2, .., 2k$ of the constant 1 and radial basis functions $g_j$ with $j \in J$ that satisfy $f_a(t_b) = \delta_{a,b}$.

Then the fundamental features $\sum_{r \in S_\gamma} f_a(|\boldsymbol{r}|)$ count the number of points with radius $t_a$, so we can assume they are the same in both configurations. According to Lemma 19, the fundamental features on $\mathbb{S}^2$ given by polynomials of degree $\leq k$ are enough to determine the sets

$$\left\{ \frac{\boldsymbol{r}}{t_a} \;\middle|\; \boldsymbol{r} \in S_\gamma, |\boldsymbol{r}| = t_a \right\}$$

for any $t_a > 0$. Therefore, using

$$\sum_{\boldsymbol{r}\in S_\gamma, |\boldsymbol{r}|=t_a} P\left(\frac{\boldsymbol{r}}{|\boldsymbol{r}|}\right) = \sum_{\boldsymbol{r}\in S_\gamma} f_a(|\boldsymbol{r}|)\cdot P\left(\frac{\boldsymbol{r}}{|\boldsymbol{r}|}\right),$$

for $t_a > 0$ we can determine also the set of original points of radius $t_a > 0$ by these fundamental features, i.e. the map $\iota$ cannot map two different configurations to the same image in $\mathcal{Y}^*$. $\square$

## I.4 Completeness of *covariant* PPSDs

For $G$–invariant functions $f$ on point configurations we can evaluate $f$ on equivalence classes of $G$–equivalent configurations, and we formulated the completeness in part 2 of Theorem 1 as "there are enough invariant polynomial functions to distinguish any two non-equivalent configurations".

We cannot formulate such a completeness criterion directly for covariant functions with values in $V$ for a non-trivial representation $(\rho, V)$: We cannot evaluate such functions on equivalence classes of $G$–equivalent configurations, since they get different values for different equivalent configurations.

However, in the above proof we embedded a space of configurations $\mathcal{X}$ by an equivariant map into a vector space $\mathcal{Y}^*$ given by fundamental features, and used the Stone–Weierstrass approximation theorem to prove the separation of orbits by approximating a continuous function that was 1 on one orbit and 0 on the other orbit. This can be used more generally as an approximation property that can be formulated also for arbitrary representations $(\rho, V)$.

The approximation property in Theorem 1 is of the form "uniform approximation on compact subsets of configurations", so we need to specify what the topological space of all configurations is:

Given the numbers of points $k_1 = |S_1|, ..., k_c = |S_c|$ of each color, we have the topological space

of all configurations with these $(k_1, ..., k_c)$ given by

$$\mathcal{X}_{(k_1,...,k_c)} = (\mathbb{R}^{3k_1}/\Sigma_{k_1}) \times ... \times (\mathbb{R}^{3k_c}/\Sigma_{k_c})$$

(Strictly speaking, this does not incorporate that the $k_i$ points of color $i$ should be different. However, as we have seen before, we can extend our PPSDs in a canonical way from taking sets as input to taking multisets as input, so this does not matter.) We consider the space of all configurations to be the disjoint union of these spaces, so the $\mathcal{X}_{(k_1,...,k_c)}$ are the connected components of the space of all configurations. This also means that a compact subset can only contain points from finitely many of these $\mathcal{X}_{(k_1,...,k_c)}$. According to part 2 of Proposition 17 we can give arbitrary polynomial functions on each of finitely many connected components, and then find a PPSD that gives these functions as restrictions to their component. (The context of Proposition 17 was the polynomial function class [case i], but in this specific part we only used that the $|S_i|$ are PPSDs, so this is equally valid in case ii.) So if we are interested in approximation on a compact subset of configurations, we can assume without loss of generality that this subset is contained in one $\mathcal{X}_{(k_1,...,k_c)}$. Furthermore, since compact subsets of Euclidean vector spaces are bounded, there must be a maximal $|\boldsymbol{r}|$ of all points in these configurations, so with $B_R$ the closed ball of radius $R$ around the origin in $\mathbb{R}^3$, we can assume that the compact subset of configurations lies in

$$\mathcal{X} := \left(B_R^{k_1}/\Sigma_{k_1}\right) \times ... \times \left(B_R^{k_c}/\Sigma_{k_c}\right)$$

for some $R > 0$. Then the statement is that any continuous $G$–covariant function $f : \mathcal{X} \to V$ can be uniformly approximated by $G$–covariant polynomial functions $P_i : \mathcal{X} \to V$. (To be precise, these continuous / polynomial functions are continuous / polynomial functions on $B_R^{k_1+...+k_c}$ that are invariant under $\Sigma_{k_1} \times ... \times \Sigma_{k_c}$.) This can then be proven for arbitrary representations $(\rho_V, V)$ in a similar way as before :

- Use the results of the previous section I.3 (which assumed Condition $\mathrm{II}_w(2k)$ in case ii) to map $\mathcal{X}$ injectively by covariant fundamental features into a finite dimensional vector space $\mathcal{Y}^*$.

- Using Stone–Weierstrass:
  Let $X' := \iota(X)$ be the image in the vector space $\mathcal{Y}^*$, this is a compact subset of $\mathcal{Y}^*$. Then any continuous function on $X$ can be seen as a continuous function on $X'$, and polynomial function on $X' \subseteq \mathcal{Y}^*$ are PPSDs. So we can use the Stone–Weierstrass theorem to approximate uniformly any continuous functions on $X'$ by polynomials.

- Averaging over the compact group $G$:
  The group $G$ acts on $\mathcal{Y}^*$ by some representation $\rho$.
  As above, we can apply an averaging operator to project functions $X \to V$ or their equivalent $X' \to V$ to equivariant functions for the representations $(\mathcal{Y}^*, \rho)$ and $(V, \rho_V)$:
  We map a function $f : X' \to V$ to the average

$$F(x) := \int_{h \in G} \rho_V^{-1}(h) f(\rho(h)x) dh \tag{14}$$

  Then

$$\begin{aligned} F(\rho(g)x) &= \int_{h \in G} \rho_V^{-1}(h) f(\rho(hg)x) dh \\ &= \int_{h \in G} \rho_V(g) \rho_V^{-1}(hg) f(\rho(hg)x) dh \\ &= \rho_V(g) \int_{h \in G} \rho_V^{-1}(hg) f(\rho(hg)x) dh \\ &= \rho_V(g) F(x) \end{aligned}$$

- Averaging does not increase the supremum norm:
  For a compact group $G$ we have a scalar product / norm on $V$ that is invariant under $\rho_V(G)$. Since all norms give the same topology on $V$, we can use this norm to prove density in the supremum norm. Then the averaging operator (14) is an isometry for

the supremum norm, and applying this to a sequence of polynomials from the Stone–Weierstrass theorem that converge to a covariant continuous function, this then gives a sequence of covariant polynomials that converge to the continuous function.

This concludes the proof of part 3 of Theorem 1.

# J Proof of Theorem 2

## J.1 Context of Theorem 2

Theorem 2 bounds the number of features necessary to give a complete characterization of configurations of $k$ points up rotations in $G$.
There are different ways to define the number of features necessary for a "complete" description of objects that can be seen as points on a manifold (/orbifold/stratified manifold) $M$:

1. The dimension of $M$.

   This gives the number of features necessary to distinguish configurations locally, i.e. in a neighborhood of a particular chosen object. Often the dimension of quotients $M = N/G$ will be $dim(N) - dim(G)$ (e.g. if $G$ is a Lie group that acts smoothly, freely and properly on a manifold $N$). In fact, in our case we have $dim(M) = 3k - 3$ for $k > 2$.

2. The embedding dimension of $M$.

   This is the number of features necessary to characterize points in $M$ globally; it is usually larger than $dim(M)$: E.g. around any given point on the sphere $M := \mathbb{S}^2 \subset \mathbb{R}^3$, two of the three coordinates are sufficient, but each particular pair of coordinates will not be enough to identify points globally, and the Borsuk–Ulam theorem implies that even when allowing two arbitrary continuous features there will always be two different (in fact, even antipodal) points on the sphere on which the two functions give the same

pair of numbers.

3. For algebraic varieties $M$, the minimal number of generators of $\mathbb{R}[M]$.

   $\mathbb{R}[M]$ is the algebra of polynomial functions on $M$, and a set of such functions is said to be generators of the algebra $\mathbb{R}[M]$ if every function in $\mathbb{R}[M]$ can be expressed as a polynomial in the generators.

   This is a quite strong condition on the basis set, and this number is usually again larger than the second number. As a simple example, let $M$ be the curve in the plane $\mathbb{R}^2$ given by $y^2 = x^3$. Then the $y$–coordinate alone already determines the point $(x, y)$, since there is a unique cube root of $y^2$ in $\mathbb{R}$, but the $x$–coordinate is not a polynomial in $y$, so just $\{y\}$ is not a set of generators. The algebra $\mathbb{R}[x, y]/(y^2 - x^3)$ of polynomials on $M$ can also be given as $\mathbb{R}[t^2, t^3] \subset \mathbb{R}[t]$ with $t := y/x$, and we can see that no single polynomial $P(t) \in \mathbb{R}[t^2, t^3]$ generates the algebra $\mathbb{R}[t^2, t^3]$: Up to an additive constant (which does not change the subalgebra generated by $P$) it has to have order 2 or higher; if it has order 2, $t^3$ cannot be written as a polynomial in $P$, and if it has order $> 2$, $t^2$ cannot be written as a polynomial in $P$, so we need at least 2 generators for the algebra of polynomials, but only one feature to distinguish points.

We are here interested in the second number (embedding dimension), and will prove that it is $\le 6k - 5$. This proof will also use the fact that the dimension is $3k - 3$, and in the case of polynomial functions, we will also show and use that the third number is finite.

To formulate Theorem 2 precisely, we need to distinguish the different use cases (see Appendix B): We have $G = O(3)$ or $G = SO(3)$, and the points are in

1. Sphere: $X = \mathbb{S}^2$
2. Spherical shell: $X = \{\boldsymbol{r} \in \mathbb{R}^3 \,\big|\, r_0 \le |\boldsymbol{r}| \le r_1\}$
3. Full space: $X = \mathbb{R}^3$

and the functions are

i. polynomials, or more generally

ii. linear combinations of products of polynomials on $\mathbb{S}^2$ and a set of radial basis functions $g_i : \mathbb{R}_{\geq 0} \to \mathbb{R}$ for $i$ in some index set $I$.

Theorem 2 is valid for cases 1, 2i, 2ii, 3i, but not 3ii. For the case 2ii we also require that the $g_i$ are analytic.

The statement of Theorem 2 is:

We consider $G$–equivalence classes of colored point sets with at most $k_1, ..., k_c$ points of colors $1, ..., c$. For $k := k_1 + ... + k_c$, we can find $2k \cdot \dim(X) - 5$ invariants (of the given function class) that already distinguish all equivalence classes.

We can consider $k$–point configurations in $X$ as points in $X^k/\Sigma$ where $\Sigma \subseteq \Sigma_k$ is the subgroup of the symmetric group $\Sigma_k$ that only permutes points with the same color. Then we can identify $X^k/(\Sigma \times G)$ with the $G$–equivalence classes of configurations, if it was a manifold, its dimension would be $d := \dim(X^k) - \dim(G) = k \cdot \dim(X) - 3$, and Theorem 2 says we can characterize points in $X/(\Sigma \times G)$ by $2d + 1$ features.

Theorem 2 is reminiscent of the embedding theorem of Whitney (see e.g.,[7] Chapter 1.8): Any smooth real $d$-dimensional manifold can be smoothly embedded in $\mathbb{R}^{2d}$ for $d > 0$.

(The slightly weaker statement that it can be embedded in $\mathbb{R}^{2d+1}$ is easier to prove, we will follow the general idea of such a proof.)

There are three main differences:

- The quotient $X^k/(\Sigma \times G)$ is not a manifold (but an orbifold, see below).
- Instead of arbitrary smooth features we only allow polynomial features (in case i, or other specific analytic features in case ii).
- We will prove a slightly stronger version which bounds the factor by which distances can shrink in this embedding.

We will prove Theorem 2 in the more detailed form:

a. There are finitely many $G$–invariants that distinguish all $G$–equivalence classes of colored point sets with at most $k_1, ..., k_c$ points of colors $1, ..., c$.

b. Given a set of $N$ $G$–invariants that distinguish all $G$–equivalence classes of colored point sets with at most $k_1, ..., k_c$ points in $X$ of colors $1, ..., c$, and $k := k_1 + ... + k_c$, we can find $2k \cdot \dim(X) - 5$ linear combinations that already distinguish all equivalence classes.

c. These $2k \cdot \dim(X) - 5$ linear combinations can be found by random orthogonal projections: After $N - 2k \cdot \dim(X) + 5$ projections to 1-codimensional subspaces we arrive at $2k \cdot \dim(X) - 5$ linear combinations of the features, with probability 1 this will be successful in the sense that they also already distinguish all equivalence classes of configurations.

d. For each such successful sequence of random projections, the distance of the projected $2k \cdot \dim(X) - 5$-dimensional feature vectors is lower bound by the distance in the original $N$–dimensional space up to a factor, i.e. there is a constant $\epsilon > 0$ such that for all configurations $\{r_i, \gamma_i\}$, $\{r'_i, \gamma'_i\}$ the distances in the feature spaces satisfy

$$d_{proj}\left(\{r_i, \gamma_i\}, \{r'_i, \gamma'_i\}\right) \geq \epsilon \cdot d_{orig}\left(\{r_i, \gamma_i\}, \{r'_i, \gamma'_i\}\right)$$

e. We required in general that the radial basis functions allow approximating polynomials, but for this theorem for a fixed $k$ it is enough that for any $2k$ different radii $t_1, ..., t_{2k}$ there are $2k$ radial basis functions $g_1, ..., g_{2k}$ such that the $2k \times 2k$–matrix $g_i(t_j)$ is nonsingular.

With these additional specification of the projection procedure and the resulting inequality for the distances, this theorem reminds of Johnson–Lindenstrauss type lemmas. However,

in the original Johnson–Lindenstrauss lemma the point set is finite, and the embedding dimension increases with the number of points. For versions in which the point set is a manifold (e.g.,[8 9]), instead of the number of points, some geometric properties of the manifold (volume, curvature) influence the embedding dimension.
In contrast, here the dimension only depends on the dimensionality of the input (stratified) manifold. (Also, the Johnson–Lindenstrauss type lemmas try to achieve an almost isometric map, they succeed with some high probability, but not with probability 1. Here we are satisfied with some lower bound on the distances in the target space, and achieve success with probability 1.)

We now turn to the proof in the following subsections.
To simplify a bit, we will first fix the numbers $|S_1|, ..., |S_c|$ of points of colors $1, 2, ..., c$. In section J.8 we will then explain why we can also encode these numbers and characterize all configurations with *at most* $k_i$ points of color $i$ without using more features.

While PPSDs are defined for configurations of any size, in this theorem we are now looking at functions on configurations of a fixed number of points. For each fixed combination $|S_1|, ..., |S_c|$ of points of colors $1, 2, ..., c$ Proposition 17 says that PPSDs on these point sets are the same functions as the polynomials in points $\boldsymbol{x}_1, ..., \boldsymbol{x}_k \in \mathbb{R}^3$ that are invariant under permutations of points of of the same color, so in the proof we will rather use these concrete polynomials in $3k$ variables.

## J.2 Finiteness

We will first look at part a, the finiteness. The previous topological completeness theorem 1 showed that *all* invariant PPSDs are enough to distinguish any two configurations that are not equivalent under $G$, but this is an infinite set, and we need a finite subset to start the projection procedure.

**Proof in case i: Polynomials**

Obviously, a finite set of invariants that distinguishes all non-equivalent configurations in $X = \mathbb{R}^3$ also gives enough invariants for other cases $X \subset \mathbb{R}^3$, so we will only consider $X = \mathbb{R}^3$. In this case we can prove that the algebra of invariant polynomials

$$\mathbb{R}[x_1, ..., x_k, y_1, ..., y_k, z_1, ..., z_k]^{\Sigma \times G}$$

is finitely generated as an algebra over $\mathbb{R}$: The group is $\Sigma \times G$ is compact, hence reductive, so this is a special case of Hilbert's finiteness theorem on invariants. (See e.g.,[10] chapter 4.3. In this reference, the ground field is assumed to be algebraically closed, but this does not make a difference: Any invariant $f \in \mathbb{C}[x_1, ..., x_k, y_1, ..., y_k, z_1, ..., z_k]$ can be written as $f = g + i \cdot h$ with $g, h \in \mathbb{R}[x_1, ..., x_k, y_1, ..., y_k, z_1, ..., z_k]$, and the operation of $\Sigma \times G$ on $f$ is just given by its operation on $g$ and $h$.)
Since the values of these finitely many generators of the algebra already determine the values of all invariant polynomials, these are enough to distinguish any two non–equivalent configurations.

**Counterexample in case 3ii**

In the case ii we have more freedom to choose the radial basis functions, hence the algebraic proof of i does not work any more. In fact, we have to make up for the relaxed restrictions on the function class by adding new assumptions, as we will show in this counterexample:

Assume $X = \mathbb{R}^3$ and as in the counterexample in section C the basis functions are (the constant 1 and) $\sin\left(\frac{2a-1}{2^b} \cdot r\right)$, and $\cos\left(\frac{2a-1}{2^b} \cdot r\right) - 1$ for natural numbers $a, b = 1, 2, ....$ We have seen in section C that although these satisfy Conditions I and II, no finite subset of these functions will be able to uniquely characterize configurations of only 1 point on the $x$–axis), i.e. Theorem 3 does not hold in the case 3ii.

We also showed in section C that Condition II (analyticity) was needed to derive Condition $\text{II}_w(2k)$, smoothness would not suffice. So in the following we assume Conditions I and II (or for this finiteness part only, assuming Condition $\text{II}_w(2k)$ only also is enough).

**Proof in case 2ii: Compact $X$, Condition $\mathbf{II}_w(2k)$**

Let $X := \{\boldsymbol{r} \in \mathbb{R}^3 \,|\, r_0 \le |r| \le r_1\}$, and we want to prove this for configurations of $k := k_1 + ... + k_c$ points. Assume Conditions I and $\text{II}_w(2k)$ (or I and II, as we showed in Appendix C, Proposition 3 that this implies Condition $\text{II}_w(n)$ for all $n$). We use the same argument as in section I.3, this time for equivalence classes of configurations under $G$. Let $J$ be the finite subset of $I$ guaranteed by condition $\text{II}_w(2k)$ for $R = r_1$. Since we are given two configurations of $k$ points, at most $2k$ radii occur in them, so let's say all radii appear in the list $t_1 < t_2 < ... < t_{2k}$. Then we can find linear combinations $f_a$ of radial basis functions $g_j$ with $j \in J$ that satisfies $f_a(t_b) = \delta_{a,b}$. Therefore features using these $f_a$ and the constant function 1 on the sphere are enough to distinguish them if they do not have the same number of points on the same radii.

Furthermore, for any polynomial feature on $\mathbb{S}^2$ we can combine this feature with the radial basis function $f_a/t_a$ for $a \in \{1, 2, ..., 2k\}$ and thus use the finiteness of necessary features for $X = \mathbb{S}^2$ and colors $\mathcal{C} \times \{1, 2, ..., 2k\}$ to deduce the required finiteness condition for our case.

## J.3 General outline for bound on number of features

The proof of Theorem 2 will depend on the the variant that we are interested in, in particular whether $X$ is compact (i.e. the sphere $X = \mathbb{S}^2$ or the spherical shell $X = \{r \in \mathbb{R}^3 \,|\, r_0 \le |\boldsymbol{r}| \le r_1\}$) or not (i.e. $X = \mathbb{R}^3$), and whether we restrict the function space to polynomials or not. In the main part we have restricted our attention to the function space of polynomials, in this case we can use algebraic geometry to prove this, see section J.5 below. If we allow more general radial basis functions, we will need to use a different theory (subanalytic sets) and will also need more assumptions, in particular that $X$ is compact.

We will give the proof for $X$ compact (i.e. sphere or spherical shell) in section J.7, assuming the radial basis functions are analytic in the interval $[r_0, r_1]$ where they are used. For $X = \mathbb{R}^3$ and polynomial features we will give the proof in section J.5.

Let $\mathcal{X}$ be the set of $G$–equivalence classes of colored point sets $\subseteq X$ with $|S_1|, ..., |S_c|$ points of colors $1, 2, ..., c$. Let $\mathcal{F}$ be a vector space of functions on $\mathcal{X}$ that separate points.

We want to show: There are $2 \cdot dim(X) \cdot k - 5$ elements of $\mathcal{F}$ that already separate points. The outline of the proof is:

1. $\mathcal{X}$ is an orbifold (see below) of $dim(\mathcal{X}) = k \cdot dim(X) - 3$.

2. According to part a) there is a finite tuple of functions $f_1, ..., f_N \in \mathcal{F}$ that separate points of $\mathcal{X}$. This defines an embedding $f : \mathcal{X} \hookrightarrow \mathbb{R}^N$.

3. Show that the set $D \subseteq \mathbb{P}^{N-1}$ of directions in $\mathbb{R}^N$ given by two different points $f(\boldsymbol{x}_1), f(\boldsymbol{x}_2)$ has a closure $\overline{D}$ of dimension $\dim(\overline{D}) \leq 2 \cdot \dim(\mathcal{X})$.

4. If $N - 1 > 2 \cdot dim(\mathcal{X})$, then there is a linear map $\mathbb{R}^N \to \mathbb{R}^{N-1}$ that is injective on $\mathcal{X}$ (and in fact only decreases distances by a factor of at most $C$ for some constant $C > 0$).

Then the theorem follows because we can start with the embedding from 1, use 2 to decrease $n$ as long as $n > 2 \cdot dim(\mathcal{X}) + 1$ (replacing the functions $f_1, ..., f_n$ by $n - 1$ linear combinations of them), and end up with $n = 2 \cdot dim(\mathcal{X}) + 1$, which is $6k - 5$ for $X = \mathbb{R}^3$ and $4k - 5$ for $X = \mathbb{S}^2$. The problem is now to find appropriate notions of functions / sets / dimensions to make this outline precise.

## J.4 Dimension of orbifolds and stratified manifolds

We will not use much of the general theories of orbifolds and stratified manifolds, only enough to give a meaning to "$dim(\mathcal{X}) = k \cdot dim(X) - 3$" (that is compatible with the definitions of dimension in real algebraic geometry and subanalytic geometry).

We can consider the set $M := X^k$ as a manifold on which the compact group $K := \Sigma \times G$ operates. In general, the quotient $M/K$ of a manifold by a compact group gives an orbifold. For example, the 1-dimensional manifold $\mathbb{R}$ divided by the group $\{\pm 1\}$ gives a quotient space that can be identified with $\mathbb{R}_{\geq 0}$. This is no longer a manifold, as the point 0 has no

neighborhood that would be homeomorphic to an open interval.

These quotients (or orbifolds in general) can be considered as stratified manifolds, see e.g. chapter 4 of,[11] the definition of stratified manifolds is also explained in chapter I.1 in.[12] In particular, stratified manifolds have a decomposition as a finite disjoint union of manifolds (usually of different dimensions). In the above example, the point 0 would be a 0-dimensional stratum, and $\mathbb{R}_{>0}$ a 1-dimensional stratum. The dimension of a stratified manifold is the largest $d$ for which there is a non–empty component of dimension $d$.

The stratification of a quotient $M/K$ is given by orbit types: For $x \in M$ denote by $K_x$ the stabilizer

$$K_x := \{g \in K \,|\, gx = x\}.$$

For any two points $x, y = gx$ in the same orbit the stabilizers are conjugate: $K_y = gK_xg^{-1}$. The conjugacy class of $K_x$ is also called the orbit type of $x$. Now Theorem 4.3.7 in[11] shows that the orbit types define a stratification of $M$, and Corollary 4.3.11 gives that the quotients of the strata by $K$ define a stratification of $M/K$.

The orbit types have a natural partial order: For two subgroups $H_1, H_2$ of $K$ and their conjugacy classes $(H_1), (H_2)$ we say $(H_1) \le (H_2)$ if $H_2$ is conjugate to a subgroup of $H_1$. Now Theorem 4.3.2 in[11] shows that there is a unique largest orbit type and the points of this orbit type are open and dense in $M$.

In our case $K = \Sigma \times G$, and an element $\kappa = (\sigma, g) \in K$ operates on a configuration $x = (\boldsymbol{r}_1, ..., \boldsymbol{r}_k) \in X^k$ by permuting points (of same color) by $\sigma$, and applying the rotation $g$ to all points. If the distances from the origin $|\boldsymbol{r}_i|$ are all different, $(\sigma, g)x = x$ can only happen for $\sigma = e$ and $g\boldsymbol{r}_i = \boldsymbol{r}_i$ for all $i = 1, 2, ..., k$. This in turn means that for $g \ne Id$, all $\boldsymbol{r}_i$ must lie on the line that is mapped to itself by $g$. So if we choose points $\boldsymbol{r}_i$ that have all different radii and do not lie on a line, $K_x = \{(e, Id)\}$. Since we can always find such points for $k > 1$, the largest orbit type is given by the neutral element in $K$, and then $K$ operates freely on the largest stratum. So for $k > 1$ the largest stratum is a manifold of dimension $\dim(X^k) - \dim(G) = k \cdot \dim(X) - 3$. (And for $k = 1$ the orbifold $M/K$ is $\mathbb{R}_{\ge 0}$ of dimension 1.)

Our stratified manifolds can be given as semi–algebraic or sub–analytic subsets of some $\mathbb{R}^N$, and instead of talking about general stratified manifolds, in the following we will use these sets and the theory of semi–algebraic / sub–analytic sets instead.

We already proved that there are features $f_1, ..., f_N$ that give a map

$$X^k \to \mathcal{X} \subseteq \mathbb{R}^N$$

which is invariant under $\Sigma \times G$, and which distinguishes any two configurations in $X^k$ that are not equivalent under $\Sigma \times G$, so the image $\mathcal{X}$ of this map can be identified with the set $X^G/(\Sigma \times G)$ in a natural way.

We will now have to define a dimension for this image (which will be the same as above) and its secant set, and the closure of the secant set, and show that they behave as expected (which will be the case for algebraic and analytic functions, but would again be wrong for smooth functions in general).

## J.5 Algebraic case

When we restrict our functions to be polynomials on $X$, this becomes a problem of algebra, which can be solved by purely algebraic means. For example, we could apply Theorem 5.3 of.[13] However, we here use a more geometric formulation that will be easier to extend to the analytic case. Since we are looking at sets in $\mathbb{R}^N$ and real–valued functions, we have to use real algebraic geometry. A standard reference is e.g.,[14] which we will use for citing the propositions that we need.

An *algebraic subset* of $\mathbb{R}^N$ is the set of zeros of some set of polynomials in $\mathbb{R}[X_1, ..., X_N]$. (Def. 2.1.1., p. 23). For example, the sphere $\mathbb{S}^2 \subseteq \mathbb{R}^3$ is an algebraic subset.

A *semi–algebraic subset* of $\mathbb{R}^N$ is a set of points that can be defined by (finitely many) polynomial equations $P(X_1, ..., X_N) = 0$ and inequalities $P(X_1, ..., X_N) > 0$ combined with

Boolean operations. (Def. 2.1.4., p.24). For example, the spherical shell $\{\boldsymbol{r} \in \mathbb{R}^3 \,\big|\, r_0 \leq |\boldsymbol{r}| \leq r_1\}$ is given by the inequalities

$$r_0^2 \leq x^2 + y^2 + z^2 \quad \wedge \quad x^2 + y^2 + z^2 \leq r_1^2,$$

so it is a semi–algebraic (but no algebraic) subset of $\mathbb{R}^3$.

We can extend that definition to maps: A map $A \to B$ is said to be semi–algebraic if its graph $\subseteq A \times B$ is semi–algebraic. For example, rational functions are semi–algebraic: Let $A \subseteq \mathbb{R}^d$ be a semi–algebraic subset on which the polynomial $g(x_1, ..., x_d)$ is not zero. Then the map $A \to \mathbb{R}$ given by the rational function

$$q(x_1, ..., x_d) := \frac{f(x_1, ..., x_d)}{g(x_1, ..., x_d)}$$

is semi–algebraic, since its graph is

$$\{\, (x_1, ..., x_d, y) \mid y \cdot g(x_1, ..., x_d) = f(x_1, ..., x_d) \,\}.$$

A fundamental theorem of real algebraic geometry is that any projection of a semi–algebraic set is again semi–algebraic (Theorem 2.2.1), this can be formulated as a model theoretic statement: This theory has "quantifier elimination" — Given any formula $\Phi(X_1, ..., X_n)$ of first–order predicate logic using only polynomial equalities and / or inequalities, the set $\{\boldsymbol{x} \in \mathbb{R}^N \,\big|\, \Phi(\boldsymbol{x})\}$ is also semi–algebraic (Proposition 2.2.4).

In particular, since the distance squared is given by a polynomial expression and the closure of a set can be described as "the points for which for each distance $d$ there is a point of distance $\leq d$ in the set", we get that the closure of a semi–algebraic set is again a semi–algebraic set (Proposition 2.2.2). Similarly the image of a semi–algebraic set under a semi–algebraic map is again a semi–algebraic set (Proposition 2.2.7), and that gives us in particular that our model $\mathcal{X} \subseteq \mathbb{R}^N$ of $X^k/(\Sigma \times G)$ is a semi–algebraic set.

The dimension of a semi–algebraic set can be defined by using Theorem 2.3.6 of:[14] All semi–algebraic sets are disjoint unions of semi–algebraic sets (semi–algebraically) homeomorphic to the $d$–dimensional open hypercube $]0,1[^d$ for some $d$ (for $d$=0 this is defined as a point). The dimension of the set is then the highest $d$ that appears in such decomposition (see chapter 2.8 in[14]). This dimension has the expected properties:

- An open semi–algebraic subset of $\mathbb{R}^n$ has dimension $n$. (Prop. 2.8.4)
- For a semi–algebraic set $A$ and a semi–algebraic map $f$, $dim(A) \geq \dim(f(A))$. (Prop 2.8.8)
- For a semi–algebraic set $A$, the closure $\bar{A}$ has $\dim(\bar{A}) = \dim(A)$. (Prop.2.8.13)

From the first two properties we also can see that this notion of dimension coincides with the dimension of a stratified manifold as described in section J.4.

Points in the (real) projective space $\mathbb{P}^{N-1}$ are given by 1-dimensional subspaces (i.e. lines through the origin) in $\mathbb{R}^N$, they are denoted by $[x_1 : ... : x_N]$ for $(x_1, ...x_N) \neq \{(0, ..., 0)\}$ with the convention that

$$[x_1 : ... : x_N] = [\lambda \cdot x_1 : ... : \lambda \cdot x_N] \quad \text{for any } \lambda \neq 0$$

We can map this set bijectively to a semi–algebraic subset of matrices in $\mathbb{R}^{N\times N}$ by

$$[x_1 : ... : x_N] \;\mapsto\; \left(\frac{x_i \cdot x_j}{\sum_m x_m^2}\right)_{i,j} \tag{15}$$

This means we encode a line in $\mathbb{P}^{N-1}$ as the orthogonal projection to that line in $\mathbb{R}^{N\times N}$. The image is given by the set of those matrices that satisfy $A^T = A, A^2 = A, \mathrm{Tr}(A) = 1$, so it is even an algebraic set (see chapter 3.4 in[14]). By looking at the usual decomposition of $\mathbb{P}^{N-1}$ into affine sets $\mathbb{R}^m$ for $m = 0, 1, ..., N-1$ one can see that $\dim(\mathbb{P}^{N-1}) = N-1$.

Now we define our secant set: Let $\Delta \subset \mathcal{X} \times \mathcal{X}$ be the diagonal

$$\Delta := \{(x,x) \,|\, x \in \mathcal{X}\}$$

and consider the map

$$sec: \quad \mathcal{X} \times \mathcal{X} \setminus \Delta \quad \longrightarrow \quad \mathbb{P}^{N-1}$$

defined by

$$\Big((x_1, ..., x_N), (y_1, ..., y_N)\Big) \;\mapsto\; [x_1 - y_1 \;:\; ... \;:\; x_N - y_N]$$

Considering $\mathbb{P}^{N-1}$ as set of matrices given by the image of (15), this becomes a semi–algebraic map (recall the remark above about rational functions being semi–algebraic).

Using these properties of semi–algebraic sets and functions, and the definition of dimension of semi–algebraic sets, we now can make the statement 3 in the outline precise (for the algebraic case): Let $D$ be the image of the semi–algebraic function *sec* defined on the semi–algebraic set $\mathcal{X} \times \mathcal{X} \setminus \Delta$, and $\bar{D}$ its closure (which is again semi–algebraic), then its dimension must be be

$$\dim(\bar{D}) = \dim(D) \leq \dim(\mathcal{X} \times \mathcal{X} \setminus \Delta) = 2 \cdot \dim(\mathcal{X}).$$

Since $\dim(\mathbb{P}^{N-1}) = N - 1$, the condition $N - 1 > 2 \cdot \dim(\mathcal{X})$ in statement 4 implies that there is a direction in $\mathbb{P}^{N-1} \setminus \bar{D}$, and that in turn means that the orthogonal projection in this direction to the orthogonal complement of this direction is injective. Since $\bar{D}$ is closed, this furthermore means there must be a positive angle $\alpha$ between this direction and the closest point in $\bar{D}$, which then translates to the fact that the orthogonal projection to the orthogonal complement multiplies distances in $\mathcal{X}$ by a factor $> \sin(\alpha) > 0$.

## J.6 Smooth case (counterexample)

In the above proof, we used for the semi–algebraic set $A := \mathcal{X} \times \mathcal{X} \smallsetminus \Delta$ and the semi–algebraic (secant) map $f$ the general formula

$$\dim(\overline{f(A)}) \leq \dim(A).$$

This formula would not be valid for smooth maps $f$: Let $A$ be the open interval $]0,1[$, this is a 1-dimensional manifold. Let $\boldsymbol{x}_i \in ]0,1[^d$ be any sequence in some $d$–dimensional hypercube. Then we can construct a smooth function $f$ with $f(1/(i+1)) = \boldsymbol{x}_i$ for $i = 1,2,3,\ldots$. Since the set of rational numbers $\mathbb{Q}$ is countable, also the set of points of the hypercube in $\mathbb{Q}^d$ is a countable set, so we can define a $f : ]0,1[ \to \mathbb{R}^d$ that goes through all these points. Since they are dense in the hypercube, we have

$$\dim(\overline{f(A)}) = d > 1 = \dim(A).$$

So the above arguments would not be valid for smooth functions. However, we will see that they work for analytic functions.

## J.7 Analytic case

In the analytic case, we will follow mostly the same arguments as in the semi–algebraic case, but use the theory of subanalytic geometry. While the properties of subanalytic sets are much more difficult to prove, using them for our purpose works almost the same as in the semi–algebraic case. A standard reference for sub–analytic geometry is e.g.,[15] see also chapter I.1 in[12] and the notes.[16]

**Definition 3** (semi–analytic subset).
*(,[12] p.43) A semi–analytic subset $A$ of $\mathbb{R}^N$ is a subset which can be covered by open sets $U \subseteq \mathbb{R}^N$ such that each $U \cap A$ is a union of connected components of sets of the form $g^{-1}(0) - h^{-1}(0)$,*

*where $g$ and $h$ belong to some finite collection of real valued analytic functions in $U$.*

Examples:

- $A = \mathbb{R}^N$ is a semi–analytic subset:

  Take $g(\boldsymbol{x}) \coloneqq 0, h(\boldsymbol{x}) \coloneqq 1$.

- $\{\boldsymbol{x} \in \mathbb{R}^N \,|\, g(\boldsymbol{x}) = 0\}$ and $\{\boldsymbol{x} \in \mathbb{R}^N \,|\, h(\boldsymbol{x}) \neq 0\}$ are semi–analytic subsets:

  Take $h(\boldsymbol{x}) \coloneqq 1$ or $g(\boldsymbol{x}) \coloneqq 0$ respectively.

- $\{\boldsymbol{x} \in \mathbb{R}^N \,|\, h(\boldsymbol{x}) > 0\}$ is a semi–analytic subset: It is a union of connected components of $\{\boldsymbol{x} \in \mathbb{R}^N \,|\, h(\boldsymbol{x}) \neq 0\}$

- The union and intersection of semi–analytic subsets are semi–analytic:

  Union by definition, for intersection use

$$\begin{aligned} g_1 = 0 \wedge g_2 = 0 \quad &\Leftrightarrow \quad g_1^2 + g_2^2 = 0 \\ h_1 \neq 0 \wedge h_2 \neq 0 \quad &\Leftrightarrow \quad h_1 \cdot h_2 \neq 0. \end{aligned}$$

- The product of semi–analytic subsets are semi–analytic subsets of the product of their manifolds.

  (Use the same formulas as for intersection.)

It follows that semi–algebraic sets are also semi–analytic; in particular, the sets $X = \{\boldsymbol{r} \in \mathbb{R}^3 \,\big|\, r_0 \leq |\boldsymbol{r}| \leq r_1\} \subset \mathbb{R}^3$ and $X^k \subset \mathbb{R}^{3k}$ are semi–analytic.

We want to derive some properties about the image of $X^k$, but the image of semi–analytic sets under analytic maps is not guaranteed to be semi–analytic, therefore we need a more general definition:

**Definition 4** (subanalytic subset / map)**.**

*(,[12] p.43) A subanalytic subset $B$ of $\mathbb{R}^N$ is a subset which can be covered by open sets $V \subseteq \mathbb{R}^N$*

*such that $V \cap B$ is a union of sets, each of which is a connected component of $f(G) - f(H)$, where $G$ and $H$ belong to some finite family $\mathcal{G}$ of semi–analytic subsets of $\mathbb{R}^{N'}$, and where $f : \mathbb{R}^{N'} \to \mathbb{R}^N$ is an analytic mapping such that the restriction of $f$ to the closure of $\cup\mathcal{G}$ is proper. A subanalytic map between two subanalytic sets is one whose graph is subanalytic.*

In particular, if $f : \mathbb{R}^{N'} \to \mathbb{R}^N$ is a proper analytic map, and $G \subseteq \mathbb{R}^{N'}$ is semi–analytic, then $f(G)$ is subanalytic in $\mathbb{R}^N$.

Note that the word "proper" here is needed, even when going to subanalytic sets it is not in general true that the image of subanalytic sets under analytic maps is again subanalytic. This is another reason why we need the restriction to compact $X$ in the analytic case.

To mimic the arguments of the semi–algebraic case, we will also use the following properties: For subanalytic sets $A, B$, also $A \times B$, $A \cap B$, $A \setminus B$, and the closure $\bar{A}$ are subanalytic. (,[15] property I.2.1.1, p.41) Also subanalytic sets have a stratification (,[15] Lemma I.2.2, p. 44) that allows to assign a dimension to subanalytic sets, and this dimension again has the property $\dim(\bar{A}) = \dim(A)$ (property I.2.1.2, p.41), and $\dim(f(A)) \leq \dim(A)$ for bounded subanalytic sets $A$ and subanalytic maps $f$ (,[16] chapter 2.3). This gives for bounded subanalytic $A$ and subanalytic $f$ the formula

$$\dim(\overline{f(A)}) \leq \dim(A)$$

and allows transferring the proof of the semi–algebraic case also to the subanalytic case.

## J.8 Variable number of points $\leq k$

Special PPSDs are

$$|S_1| = \sum_{\boldsymbol{r} \in S_1} 1 \ , \quad \dots \ , \quad |S_c| = \sum_{\boldsymbol{r} \in S_c} 1$$

There are only finitely many value combinations that these functions can take for configurations of $\leq k$ points. (In fact, their number is $\binom{k+c}{c}$, although the concrete number is not important for the following arguments).

To treat configurations of less than $k$ points together with configurations of $k$ points, let us adopt the convention that the points are enumerated by first writing down the $|S_1|$ points of color 1, then the $|S_2|$ points of color 2, ..., then the $|S_c|$ points of color $S_c$, and then add $k - |S_1| - ... - |S_c|$ points at the origin $(0,0,0)$.

Since $X$ is compact, each feature $f$ has a bounded image. So we can encode the finite amount of information in the $|S_1|, ..., |S_c|$ together with $f$ in one feature

$$f + C \cdot |S_1| + C^2 \cdot |S_2| + ... + C^c \cdot |S_c|$$

for $C$ large enough.

# K Proof of Theorem 3

**Part 1:** "All scalar PPSDs are some linear combination of fields in this schema."

First row: In case i, this is the well known statement that harmonic functions are linear combinations of spherical harmonics, and that polynomials of degree $n$ are $|\boldsymbol{r}|^2\cdot$ polynomials of degree $n-2 \oplus$ harmonic polynomials of degree $n$, see e.g.,[17] chapter 7, Prop. 2.7.

In case ii, this follows from the definitions and the statement for $X = \mathbb{S}^2$.

Following rows: By definition, the PPSDs of order $d$ are polynomials of order $d$ in the fundamental features, so as a vector space they are spanned by the products of PPSDs of order $d-1$ and PPSDs of order 1.

If $f, g$ are scalar components of the vector values functions $F, G$, then the product $f \cdot g$ appears as scalar component in $F \otimes G$.

**Part 2:** "Any $SO(3)$–*covariant* PPSD with values in $\mathcal{H}^{(l)}$ is a linear combination of vectors in the $l$–th column."

In column $l$ we give the vectors of $\mathcal{H}^{(l)}$ as $2l+1$ entries in the standard basis $\boldsymbol{e}_{-l}, ..., \boldsymbol{e}_l$ of $\mathcal{H}^{(l)}$ such that the entries correspond to the standard real spherical harmonics. Each such vector

of PPSDs corresponds to a covariant function from the configurations to $\mathcal{H}$, or equivalently, a covariant map from $\mathcal{H}$ to the vector space $\mathcal{F}$ of PPSDs, so all functions occurring in any field in the $l$–th column is in the isotypic component of $\mathcal{H}^{(l)}$ in $\mathcal{F}$.
It remains to show that these functions only fit together in one way, i.e. that any given collection on $2l+1$ PPSDs, of which each individually can be written as linear combination of functions occurring in fields of the $l$–th column, can also as a whole vector be written as linear combination of vectors.

The vector space and $G$–representation of all functions in the $l$–th column and row $d$ is usually infinite dimensional, but we can write it as a union of finite dimensional pieces: If we limit ourselves to fundamental features of $l \leq l_{max}$ and a finite subspace $\mathcal{F}_{fin} \subseteq \mathbb{R}+\mathcal{R}$ of radial functions, then we have only finitely many fundamental features and hence also only finitely many products of degree $\leq d$. Therefore in the following we can use the theory of finite dimensional representations even when talking about infinite dimensional representations.

Consider the $G$–representation on the vector space $\mathcal{F}_{l,d}$ which is the isotypic component of $\mathcal{H}^{(l)}$ of all PPSDs of degree $d$ (i.e. occurring in the $d$–th row). We can write it (if necessary, restrict to finite dimensional subspaces as explained above) as $\mathcal{H}^{(l)} \oplus ... \oplus \mathcal{H}^{(l)}$, see G.4. Now any $G$–equivariant function on configurations gives a covariant map $\mathcal{H}^{(l)} \to \mathcal{H}^{(l)} \oplus ... \oplus \mathcal{H}^{(l)}$, so by the Lemma of Schur it must have the form $\boldsymbol{v} \mapsto \alpha_1 \boldsymbol{v} \oplus ... \oplus \alpha_m \boldsymbol{v}$. Given our standard base $\boldsymbol{e}_{-l}, ..., \boldsymbol{e}_l$ this means that the $m \cdot (2l+1)$–dim vector space $\mathcal{H}^{(l)} \oplus ... \oplus \mathcal{H}^{(l)}$ can be written as the direct sum of $2l+1$ pieces $\boldsymbol{e}_i \cdot \mathbb{R}$ (only as vector space, not as representation), and hence any function in one of the $2l+1$ pieces can only be written as linear combination of functions in the same piece. Furthermore, since also the our given new function must be of the form $\boldsymbol{v} \mapsto \alpha_1 \boldsymbol{v} \oplus ... \oplus \alpha_m \boldsymbol{v}$, the linear combination must be the same for all components, i.e. any covariant PPSD with values in $\mathcal{H}^{(l)}$ must be a linear combination of the covariant PPSDs with values in $\mathcal{H}^{(l)}$ that occur in our schema.

**Part 3:** "Any $O(3)$–*covariant* PPSD with values in $\mathcal{H}^{(l)}$ is a linear combination of vectors

in the $l$–th column of the appropriate parity."

As stated in B.1, $O(3)$ is the direct product of $SO(3)$ and $\{Id, -Id\}$. The irreducible representations of $O(3)$ are just the irreducible representations $\mathcal{H}^{(l)}$ of $SO(3)$ together with the information whether $\rho(-Id)$ acts by multiplication by +1 (even representation) or −1 (odd representation), we denote the corresponding $O(3)$–representations as $\mathcal{H}^{(l,+)}$ or $\mathcal{H}^{(l,-)}$. So to be precise, we should specify whether we want a $O(3)$–covariant PPSD with values in $\mathcal{H}^{(l,+)}$ or $\mathcal{H}^{(l,-)}$.

On points and configurations we have the canonical operation of $O(3)$, and also for functions on $\mathbb{R}^3$ or for PPSDs (i.e. functions on configurations) we get a canonical operation of $O(3)$. Given two irreducible $O(3)$–representations $\mathcal{H}^{(l_1,p_1)}$ and $\mathcal{H}^{(l_2,p_2)}$, the Clebsch–Gordon decomposition writes their tensor product as a direct sum

$$\mathcal{H}^{(l_1,p_1)} \otimes \mathcal{H}^{(l_2,p_2)} \simeq \mathcal{H}^{(|l_1-l_2|,p)} \oplus \ldots \oplus \mathcal{H}^{(l_1+l_2,p)} \quad \text{with } p = p_1 \cdot p_2.$$

In our schema all PPSDs have either the property that they are invariant under $(x, y, z) \mapsto (-x, -y, -z)$ (i.e. are even) or reverse the sign (i.e. are odd): The fundamental features given by $\sum_{\boldsymbol{r} \in S_\gamma} Y_l(\boldsymbol{r})$ in the first row are even / odd when the degree $l$ is even / odd, and then the Clebsch–Gordon decomposition gives again only features that are either even or odd. For example, the fundamental features given by $Y_1$ are odd, i.e. lie in a $\mathcal{H}^{(1,-)}$, and taking the Clebsch–Gordon product of two such features gives results in $\mathcal{H}^{(0,+)}, \mathcal{H}^{(1,+)}$ and $\mathcal{H}^{(2,+)}$.

Since all entries in our schema are already sorted into even and odd, the claim of part 3 follows from part 2.

# L Proof of Theorem 4

*Proof of parts 1, 2.*

Let $G = SO(3)$ or $O(3)$. Lemma 16 (uniqueness of the normal form) also describes the oper-

ation of $G$ on PPSDs $\mathcal{D}$ in terms of its operation on polynomials: Let $\mathcal{D}$ be written in normal form as a sum of terms $\mathcal{E}_{m_1,\ldots,m_c;e_1,\ldots,e_c}$ and which contain the polynomials $P_{m_1,\ldots,m_c;e_1,\ldots,e_c}$ as in (7). Applying a $g \in G$ to $\mathcal{D}$ can be done by applying $g$ to the polynomials $P_{m_1,\ldots,m_c;e_1,\ldots,e_c}$, and this gives a representation of $g\mathcal{D}$ which is again in Normal Form. Since the Normal Form is unique, this gives the unique way that $g$ operates on PPSDs given in Normal Form. In particular, if a PPSD is invariant under $G$, the polynomials $P_{m_1,\ldots,m_c;e_1,\ldots,e_c}$ must be invariant under $G$.

We now can apply the First Fundamental Theorem of Invariant Theory for the group $G$, see e.g.[18], section 11.2.1, p. 390: This polynomial $P_{m_1,\ldots,m_c;e_1,\ldots,e_c}$ in the vectors $\boldsymbol{r}^{(1)}, \boldsymbol{r}^{(2)}, \ldots, \boldsymbol{r}^{(m)} \in \mathbb{R}^3$ (with $m = m_1 + \ldots + m_c$) can be written as a polynomial in expressions of the form $\langle \boldsymbol{r}^{(i)}, \boldsymbol{r}^{(j)} \rangle$ for $G = O(3)$, and additionally expressions of the form $\det(\boldsymbol{r}^{(i)}, \boldsymbol{r}^{(j)}, \boldsymbol{r}^{(k)})$ for $G = SO(3)$. To prove Theorem 4, it is then enough to show that all parts corresponding to products of scalar products (and determinants) can be written as a contraction of a tensor product of moment tensors (and $\epsilon_{ijk}$).

We rewrite such products in tensor notation, but we don't use the Einstein summation convention which may obscure the change of summation order that will be used here. For a vector $\boldsymbol{v} \in \mathbb{R}^3$ we write $t_i(\boldsymbol{v})$ for the $i$–th component of $\boldsymbol{v}$, and more general we write $t_i(\boldsymbol{v})$, $t_{ij}(\boldsymbol{v})$, $t_{ijk}(\boldsymbol{v})$etc. for the tensors $v$, $v^{\otimes 2}$, $v^{\otimes 3}$ etc., i.e. $t_{ij}(\boldsymbol{v}) \coloneqq t_i(\boldsymbol{v}) \cdot t_j(\boldsymbol{v})$ etc. For a variable $\boldsymbol{r}$ summing over the points in $S_\gamma$ of some color $\gamma$ we then get the moment tensors as

$$T_{i_1,\ldots,i_k}(\gamma) \coloneqq \sum_{\boldsymbol{r} \in S_\gamma} t_{i_1,\ldots,i_k}(\boldsymbol{r})$$

This gives now the following recipe to rewrite a sum over points of products of scalar products and determinants as a contraction of a products of moment tensors and (optionally) Levi–Civita symbols:

- Rewrite scalar products $\langle \boldsymbol{r}^{(a)}, \boldsymbol{r}^{(b)} \rangle$ as $\sum_i t_i(r^{(a)}) t_i(r^{(b)})$ (using new indices for every new factor).

- Rewrite determinants $\det(\boldsymbol{r}^{(a)}, \boldsymbol{r}^{(b)}, \boldsymbol{r}^{(c)})$ as $\sum_{i,j,k} \epsilon_{ijk} t_i(r^{(a)}) t_j(r^{(b)}) t_k(r^{(c)})$ (using new indices for every new factor).

- Move the summations over coordinate indices $\{1, 2, 3\}$ to the left of the the summations over the points.

- Replace the product of $k$ vector components $t_{i_j}(\boldsymbol{r})$ involving the same vector variable $\boldsymbol{r}$ by one tensor expression $t_{i_1,\ldots,i_k}(\boldsymbol{r})$.

- Replace the sum over one variable $\sum_{\boldsymbol{r} \in S_\gamma}$ and the corresponding expression $t_{i,\ldots}(\boldsymbol{r})$ by the moment tensor $T_{i,\ldots}(\gamma)$

After these transformations, we have the rewritten the PPSD $\mathcal{D}$ as a linear combination of contractions of moment tensors and Levi–Civita symbols, as required by Theorem 4.

We illustrate this recipe in a generic example:

$$
\begin{aligned}
&\sum_{\boldsymbol{r}^{(1)} \in S_1} \sum_{\boldsymbol{r}^{(2)} \in S_1} \sum_{\boldsymbol{r}^{(3)} \in S_2} \langle \boldsymbol{r}^{(1)}, \boldsymbol{r}^{(1)} \rangle \cdot \langle \boldsymbol{r}^{(1)}, \boldsymbol{r}^{(2)} \rangle \cdot \det(\boldsymbol{r}^{(1)}, \boldsymbol{r}^{(2)}, \boldsymbol{r}^{(3)}) \\
&= \sum_{\boldsymbol{r}^{(1)} \in S_1} \sum_{\boldsymbol{r}^{(2)} \in S_1} \sum_{\boldsymbol{r}^{(3)} \in S_2} \left( \sum_{i=1}^{3} t_i(\boldsymbol{r}^{(1)}) t_i(\boldsymbol{r}^{(1)}) \right) \cdot \left( \sum_{j=1}^{3} t_j(\boldsymbol{r}^{(1)}) t_j(\boldsymbol{r}^{(2)}) \right) \\
&\qquad \cdot \left( \sum_{k,l,m=1}^{3} \epsilon_{klm} t_k(\boldsymbol{r}^{(1)}) t_l(\boldsymbol{r}^{(2)}) t_m(\boldsymbol{r}^{(3)}) \right) \\
&= \sum_{\boldsymbol{r}^{(1)} \in S_1} \sum_{\boldsymbol{r}^{(2)} \in S_1} \sum_{\boldsymbol{r}^{(3)} \in S_2} \sum_{i,j,k,l,m} t_{iijk}(\boldsymbol{r}^{(1)}) t_{jl}(\boldsymbol{r}^{(2)}) t_m(\boldsymbol{r}^{(3)}) \epsilon_{klm} \\
&= \sum_{i,j,k,l,m} \sum_{\boldsymbol{r}^{(1)} \in S_1} \sum_{\boldsymbol{r}^{(2)} \in S_1} \sum_{\boldsymbol{r}^{(3)} \in S_2} t_{iijk}(\boldsymbol{r}^{(1)}) t_{jl}(\boldsymbol{r}^{(2)}) t_m(\boldsymbol{r}^{(3)}) \epsilon_{klm} \\
&= \sum_{i,j,k,l,m} \left( \sum_{\boldsymbol{r}^{(1)} \in S_1} t_{iijk}(\boldsymbol{r}^{(1)}) \right) \cdot \left( \sum_{\boldsymbol{r}^{(2)} \in S_1} t_{jl}(\boldsymbol{r}^{(2)}) \right) \cdot \left( \sum_{\boldsymbol{r}^{(3)} \in S_2} t_m(\boldsymbol{r}^{(3)}) \right) \\
&= \sum_{i,j,k,l,m} T_{iijk}(\gamma_1) T_{jl}(\gamma_1) T_m(\gamma_2) \epsilon_{klm}
\end{aligned}
$$

To show that one Levi–Civita symbol is enough, we compute explicitly the product of two

such symbols:

$$\begin{aligned}
\sum_{i,j,k=1}^{3} \epsilon_{ijk} \cdot \epsilon_{ijk} &= 6 \\
\sum_{i,j=1}^{3} \epsilon_{ijk} \cdot \epsilon_{ijl} &= 6 \cdot \delta_{lk} \\
\sum_{i=1}^{3} \epsilon_{ijk} \cdot \epsilon_{ilm} &= 3 \cdot \delta_{jl}\delta_{km} - 3 \cdot \delta_{jm}\delta_{kl} \\
\epsilon_{ijk} \cdot \epsilon_{lmn} &= \delta_{il}\delta_{jm}\delta_{kn} + \delta_{im}\delta_{jn}\delta_{kl} + \delta_{in}\delta_{jl}\delta_{km} \\
&\quad -\delta_{il}\delta_{jn}\delta_{km} - \delta_{in}\delta_{jm}\delta_{kl} - \delta_{im}\delta_{jl}\delta_{kn}
\end{aligned}$$

We see that in each case, the result can be expressed by Dirac deltas. Any unpaired index in these $\epsilon$–products, i.e. an index $i$ occurring on a $\delta_{ij}$, has to also occur in some other tensor. In that case $i$ can be replaced by $j$ in the other tensor while deleting the $\delta_{ij}$ and the summation over $i$. So in a contraction of a tensor product to a scalar, any $\delta_{ij}$ can be eliminated, and hence any tensor product with at least two Levi–Civita symbols can be written as a linear combination of contractions of tensor products with 2 less Levi–Civita symbols. Repeating this procedure arrives at tensor products / contractions with at most one Levi–Civita symbol in each product. □

*Proof of part 3.*

Assume we have two different sets of colored points with at most $n$ points that cannot be distinguished by $O(3)$–invariant PPSDs, but are not related by a rotation from $O(3)$. Since the moment tensors of order 0 give the numbers $|S_1| = n_1, ..., |S_c| = n_c$, we can assume that these numbers are the same for both sets. We set $n = n_1 + ... + n_c$.

For the first option, Lemma 19 says that tensor moments of orders $\leq n$ are sufficient to distinguish between point configurations, and then Proposition 20 showed that any two configurations that are not $O(3)$–equivalent can be distinguished by $O(3)$–invariant polynomials on the vector space of these moments.

For the second option, we can use Lemma 15, which gives us for any invariant that distinguishes two given point configurations of $\leq n$ points another invariant that has at most $n$ arguments and gives the same values on our configurations of $n$ points, i.e. will also distinguish them.

It is tempting to combine both, by applying Lemma 15 to the invariant that only uses the first $n$ moment tensors. However, Lemma 15 takes expressions $\mathcal{E}_P$ and writes them as linear combinations of $\mathcal{E}_Q$ with $Q = P/\sim$ having less arguments, but this will increase the degree in the arguments, so we loose the degree bound from Lemma 19.

So for the third option, we will use instead a general result from the invariant theory of permutation groups, see[19], Theorem 3.4.2 / Corollary 3.4.3 (Göbel's Theorem):

Let $G$ be a finite group, $X$ a finite set (considered as "symbols" or "variables") on which $G$ operates by permutations. Then the ring $\mathbb{R}[X]^G$ is generated as an algebra by polynomials in $X$ of degree at most $\max\left\{|X|, \binom{|X|}{2}\right\}$.

Since the PPSDs restricted to point sets $|S_1| = n_1, ..., |S_c| = n_c$ can be given by polynomials in $n := n_1 + ... + n_c$ variables that are invariant under $\Sigma_{n_1} \times ... \times \Sigma_{n_c}$ (part 1 of Proposition 17), we can argue directly with these polynomials.

By the FFT of invariant theory of $O(3)$ (or by direct geometric arguments), the list of $n(n+1)/2$ scalar products $s_{ij} := \langle \boldsymbol{r}_i, \boldsymbol{r}_j \rangle$ determines the list of $\boldsymbol{r}_i$ up to $O(3)$. Since we assume we have two $O(3)$–inequivalent point sets, we can distinguish them by their lists $s_{ij}$. On these lists of $n(n+1)/2$ numbers the finite group $\Sigma_{n_1} \times ... \times \Sigma_{n_c}$ operates by some permutations of the $s_{ij}$ (which we get by permuting the $i$'s and $j$'s), and the $s_{ij}$–lists from our two configurations must have different $\Sigma_{n_1} \times ... \times \Sigma_{n_c}$–orbits. Again by Proposition 20 this means that there must by a $\Sigma_{n_1} \times ... \times \Sigma_{n_c}$–invariant polynomial in the variables $s_{ij}$, $1 \leq i \leq j \leq n$ on these lists that distinguishes these two orbits. Now we can apply Göbel's Theorem mentioned above, and get that such a polynomial must exist even for degree at

most $\max\left\{|X|, \binom{|X|}{2}\right\}$ with $|X| = n(n+1)/2$ for $n > 1$, which gives the upper bound

$$\frac{n(n+1)}{2} \cdot \frac{n(n+1)-2}{2} \Big/ 2 \quad < \quad \frac{n^2(n+1)^2}{8}$$

for the degree in the $s_{ij}$. Since $s_{ij} = \langle \boldsymbol{r}_i, \boldsymbol{r}_j \rangle$ is of degree 2 in the $\boldsymbol{r}_i$, we get the upper bound from Theorem 4. □

This statement / proof can also be easily adapted to the case $G = SO(3)$: In that case, we can add expressions $d_{ijk} := \det(\boldsymbol{r}_i, \boldsymbol{r}_j, \boldsymbol{r}_k)$ to the set $X$ of variables and then follow the same argument.

# M Matrix moments examples

## M.1 $3 \times 3$ matrices

The simplest example of matrix moments occurs for $a = b = 1$, i.e. $3 \times 3$ matrices: From

$$\mathcal{H}^{(1)} \otimes \mathcal{H}^{(1)} \simeq \mathcal{H}^{(0)} \oplus \mathcal{H}^{(1)} \oplus \mathcal{H}^{(2)}$$

we see that the 9–dimensional space of $3 \times 3$–matrices decomposes into irreducible subrepresentations of dimensions 1,3,5, these are given by

- Multiples of the identity matrix,
- antisymmetric matrices,
- symmetric matrices of trace 0.

The corresponding moment matrices $M_l := M_{1,1,l}$ are

$$M_0 = \begin{pmatrix} 1 & 0 & 0 \\ 0 & 1 & 0 \\ 0 & 0 & 1 \end{pmatrix}$$

and

$$M_1 = \begin{pmatrix} 0 & -z & y \\ z & 0 & -x \\ -y & x & 0 \end{pmatrix}$$

and

$$M_2 = \begin{pmatrix} \frac{2x^2-y^2-z^2}{3} & xy & xz \\ xy & \frac{2y^2-x^2-z^2}{3} & yz \\ xz & yz & \frac{2z^2-x^2-y^2}{3} \end{pmatrix}$$

We observe that $M_1^2 = M_2 - \frac{2}{3}r^2 Id$, which corresponds to the fact that $M_1 \cdot M_1$ encodes both the scalar product $r^2 = \boldsymbol{r}^T \cdot \boldsymbol{r}$ of

$$\boldsymbol{r} := \begin{pmatrix} x \\ y \\ z \end{pmatrix}$$

with itself and the outer product $\boldsymbol{r} \cdot \boldsymbol{r}^T$, while the vector product $\boldsymbol{r} \times \boldsymbol{r} = 0$. Readers may want to convince themselves that the matrix product $M_1 \cdot M_1'$ corresponding to two different vectors $\boldsymbol{r}, \boldsymbol{r'}$ indeed encodes scalar product, vector product, and outer product of $\boldsymbol{r}$ and $\boldsymbol{r'}$. (To get the $\mathcal{H}^{(2)}$–component of the product, we have to subtract from the outer product the appropriate multiple of the identity to get a traceless symmetric matrix.)

## M.2 $5 \times 5$ matrices

The $5 \times 5$ moment matrices $M_l := M_{2,2,l}$ with $0 \leq l \leq 2$ are

$$M_0 = \begin{pmatrix} 1 & 0 & 0 & 0 & 0 \\ 0 & 1 & 0 & 0 & 0 \\ 0 & 0 & 1 & 0 & 0 \\ 0 & 0 & 0 & 1 & 0 \\ 0 & 0 & 0 & 0 & 1 \end{pmatrix}, \qquad M_1 = \begin{pmatrix} 0 & 2x & z & -y & 0 \\ -2x & 0 & y & z & 0 \\ -z & -y & 0 & x & -\sqrt{3}y \\ y & -z & -x & 0 & \sqrt{3}z \\ 0 & 0 & \sqrt{3}y & -\sqrt{3}z & 0 \end{pmatrix}$$

$$M_2 = \begin{pmatrix} -2x^2+y^2+z^2 & 0 & 3xy & 3xz & -2\sqrt{3}yz \\ 0 & -2x^2+y^2+z^2 & -3xz & 3xy & \sqrt{3}\left(z^2-y^2\right) \\ 3xy & -3xz & x^2-2y^2+z^2 & 3yz & \sqrt{3}xz \\ 3xz & 3xy & 3yz & x^2+y^2-2z^2 & \sqrt{3}xy \\ -2\sqrt{3}yz & \sqrt{3}\left(z^2-y^2\right) & \sqrt{3}xz & \sqrt{3}xy & 2x^2-y^2-z^2 \end{pmatrix} \tag{16}$$

The matrix $M_2$ can also be given as

$$M_2 = M_1^2 + 2r^2 \cdot Id \qquad \text{with} \quad r^2 := x^2+y^2+z^2$$

The $M_i$ are antisymmetric for odd $i$, we write them as $D_i - D_i^T$ with upper triangular matrices $D_i$.

For even $i$, the $M_i$ are symmetric, we write them as $D_i + diag(d_i) + D_i^T$ with upper triangular matrices $D_i$ and diagonal matrices with entries $d_i$.

Then $D_2, d_2$ are given as

$$\begin{pmatrix} 0 & 0 & 3xy & 3xz & -2\sqrt{3}yz \\ 0 & 0 & -3xz & 3xy & \sqrt{3}\left(z^2-y^2\right) \\ 0 & 0 & 0 & 3yz & \sqrt{3}xz \\ 0 & 0 & 0 & 0 & \sqrt{3}xy \\ 0 & 0 & 0 & 0 & 0 \end{pmatrix}, \quad \begin{pmatrix} r^2-3x^2 \\ r^2-3x^2 \\ r^2-3y^2 \\ r^2-3z^2 \\ 3x^2-r^2 \end{pmatrix}$$

The matrices $D_3, D_4$ are

$$D_3 = \begin{pmatrix} 0 & 3r^2x-5x^3 & 10z^3-6r^2z & 6x^2y-4y^3+6yz^2 & 5\sqrt{3}\left(xz^2-xy^2\right) \\ 0 & 0 & -6x^2y-y^3+9yz^2 & -6x^2z+9y^2z-z^3 & 10\sqrt{3}xyz \\ 0 & 0 & 0 & 10x^3-6r^2x & \sqrt{3}\left(r^2y-5x^2y\right) \\ 0 & 0 & 0 & 0 & -\sqrt{3}\left(r^2z-5x^2z\right) \\ 0 & 0 & 0 & 0 & 0 \end{pmatrix}$$

and

$$D_4 = \begin{pmatrix} 0 & 70yz\left(y^2-z^2\right) & -20xy\left(r^2-7z^2\right) & -20xz\left(r^2-7y^2\right) & -10\sqrt{3}yz\left(r^2-7x^2\right) \\ 0 & 0 & 10xz\left(2x^2+9y^2-5z^2\right) & -10xy\left(2x^2-5y^2+9z^2\right) & 5\sqrt{3}\left(6x^2\left(y^2-z^2\right)-y^4+z^4\right) \\ 0 & 0 & 0 & -20yz\left(r^2-7x^2\right) & 10\sqrt{3}xz\left(7x^2-3r^2\right) \\ 0 & 0 & 0 & 0 & 10\sqrt{3}xy\left(7x^2-3r^2\right) \\ 0 & 0 & 0 & 0 & 0 \end{pmatrix}$$

and the diagonal entries of $M_4$ are

$$d_4 = \begin{pmatrix} 4x^4-12x^2y^2-12x^2z^2-16y^4+108y^2z^2-16z^4 \\ 4x^4-12x^2y^2-12x^2z^2+19y^4-102y^2z^2+19z^4 \\ -16x^4-12x^2y^2+108x^2z^2+4y^4-12y^2z^2-16z^4 \\ -16x^4+108x^2y^2-12x^2z^2-16y^4-12y^2z^2+4z^4 \\ 24x^4-72x^2y^2-72x^2z^2+9y^4+18y^2z^2+9z^4 \end{pmatrix}$$

# N Proof of Theorem 5

## N.1 The representation on matrices

The Clebsch–Gordan relation gives the isomorphism

$$\mathcal{H}^{(|a-b|)} \oplus \mathcal{H}^{(|a-b|+1)} \oplus \ldots \oplus \mathcal{H}^{(a+b)} \simeq \mathcal{H}^{(a)} \otimes \mathcal{H}^{(b)}, \tag{17}$$

and since $\mathcal{H}^{(a)}$ is also isomorphic to its dual, this can be identified with linear maps ${\mathcal{H}^{(a)}}^* \otimes \mathcal{H}^{(b)} = Lin(\mathcal{H}^{(a)}, \mathcal{H}^{(b)})$ (see section G.5). If the elements of $\mathcal{H}^{(a)}$ and $\mathcal{H}^{(b)}$ are expressed numerically as $(2a+1)$–dimensional vectors $\boldsymbol{w}$ and $(2b+1)$–dimensional vectors $\boldsymbol{v}$, this identifies $\boldsymbol{v} \otimes \boldsymbol{w}$ with the $(2b+1) \times (2a+1)$ matrix $\boldsymbol{v} \cdot \boldsymbol{w}^T$.

In fact, 17 is also valid for $a, b \in \frac{1}{2} + \mathbb{N}$ as representations of the double cover of $SO(3)$, which can be identified with $SU(2)$. While a "rotation by $2\pi$" in the double cover of $SO(3)$ operates as $-1$ on $\mathcal{H}^{(a)}$ and $\mathcal{H}^{(b)}$, it then operates as identity on $\mathcal{H}^{(a)} \otimes \mathcal{H}^{(b)}$, so $\mathcal{H}^{(a)} \otimes \mathcal{H}^{(b)}$ is again a representation of $SO(3)$, which we can interpret as $(2a+1)\times(2b+1)$–matrices, which are now matrices with *even* side lengths. However, in practice this may be less attractive since it would require computations with complex numbers, so we are not exploring this further in this paper.

Note that the "matrix of matrices" computations are just using reducible representations $V, W$ to form matrices $Lin(V, W)$: The reducible representations are written as direct sums

$$V = \bigoplus_a \mathcal{H}^{(a)}, \qquad W = \bigoplus_b \mathcal{H}^{(b)}$$

and this gives the partition of the total side length of the big matrices into the side lengths $(2a+1)$ or $(2b+1)$ of the smaller matrices.

We can also generalize this from matrices to tensors of arbitrary order: Repeated application of the Clebsch–Gordan isomorphism gives a decomposition into irreducible representations

of any tensor products (of irreducible representations, or of arbitrary representations that come with a decomposition into irreducible ones).

We will see in the next section that any bilinear product between representations can be built up from Clebsch–Gordan representations, so this applies in particular to matrix products and more generally to tensor contractions. So we could also generalize the matrix based features of appendix E to tensor based features.

## N.2 Using Schur's lemma

As explained in Section G.3, Schur's lemma says that any morphism between irreducible representations is 0 if the representations are not isomorphic, and the only morphisms from an irreducible representation to itself are scalars for odd dimensional representation over $\mathbb{R}$. (For our $G$, all irreducible representations are odd dimensional.)

Let $\mathcal{H}^{(a)} \subseteq U$ and $\mathcal{H}^{(b)} \subseteq V$ be some irreducible components of $G$–representations on $U$ and $V$, let $\circ : U \times V \to W$ be a bilinear covariant map, and $W \to \mathcal{H}^{(c)}$ be the orthogonal projection to an irreducible component $\mathcal{H}^{(c)}$ of $W$. Then we can restrict this bilinear map to the subspaces $\mathcal{H}^{(a)} \subseteq U$ and $\mathcal{H}^{(b)} \subseteq V$ and get a covariant map

$$\mathcal{H}^{(a)} \otimes \mathcal{H}^{(b)} \hookrightarrow U \otimes V \xrightarrow{\circ} W \to \mathcal{H}^{(c)}. \tag{18}$$

Since

$$\mathcal{H}^{(a)} \otimes \mathcal{H}^{(b)} \simeq \mathcal{H}^{(|a-b|)} \oplus \mathcal{H}^{(|a-b|+1)} \oplus \ldots \oplus \mathcal{H}^{(a+b)},$$

formula (4) says that $Hom_G(\mathcal{H}^{(a)} \otimes \mathcal{H}^{(b)}, \mathcal{H}^{(c)})$ is one dimensional if $|a-b| \leq c \leq a+b$ and zero dimensional else. Since the Clebsch–Gordan map is nonzero, the map (18) can only be non–zero if $|a-b| \leq c \leq a+b$ and then it must be a composition of the Clebsch–Gordan product and the multiplication by a scalar $\lambda$:

$$\mathcal{H}^{(a)} \otimes \mathcal{H}^{(b)} \xrightarrow{\text{Clebsch–Gordan}} \mathcal{H}^{(c)} \xrightarrow{\cdot\lambda} \mathcal{H}^{(c)}. \tag{19}$$

Repeating this argument for all other $\mathcal{H}^{(a)}, \mathcal{H}^{(b)}, \mathcal{H}^{(c)}$ in a decomposition of $U, V, W$ into irreducible representations, we see that any bilinear covariant map $U \times V \to W$ must be a linear combination of Clebsch–Gordan maps applied to irreducible components of $U$, $V$, and $W$.

In particular, this applies to the matrix product (or, more generally, also any contraction of two tensors).

Since bilinear covariant maps are linear combinations of Clebsch–Gordan operations, we can reasonably hope that we may also be able to go the other way round and recover the Clebsch–Gordan operations from enough bilinear covariant maps.

To show that the resulting features satisfy an algebraic completeness theorem, we have to show that every sequence of Clebsch–Gordan operations really appears with a non–zero coefficient in our procedure using a particular bilinear covariant operation.

In the following section, we will prove that for products of two matrices, the $\lambda$ in (19) is always nonzero, and then we will use that in the following section to show that we can indeed write every sequence of Clebsch–Gordon operations as a sequence of matrix multiplications (up to a non–zero factor).

## N.3 Using complex spherical harmonics

We introduced the operation of $G$ on the real vector spaces $\mathcal{H}^{(l)}$, which assigns to each $g \in G$ a $(2l+1) \times (2l+1)$–matrix $\rho(g)$ with real entries. But we can as well interpret the $\rho(g)$ as matrices of complex numbers, i.e. as endomorphisms of a complex vector space $\mathcal{H}^{(l)} \otimes_{\mathbb{R}} \mathbb{C}$. The real spherical harmonic functions are a basis of $\mathcal{H}^{(l)}$ both as $\mathbb{R}$–vector space and of $\mathcal{H}^{(l)} \otimes_{\mathbb{R}} \mathbb{C}$ as $\mathbb{C}$–vector space. Another basis of $\mathcal{H}^{(l)} \otimes_{\mathbb{R}} \mathbb{C}$ is given by the complex spherical harmonic functions. While for numerical computations the real vector space $\mathcal{H}^{(l)}$ is usually preferable, the representation on the complex vector space $\mathcal{H}^{(l)} \otimes_{\mathbb{R}} \mathbb{C}$ is easier to use for theoretical purposes.

In this section we will use the complex version, which we simply denote again by $\mathcal{H}^{(l)}$ in the

rest of this section.

We use the basis of the Lie algebra $\mathfrak{so}(3)$:

$$l_x = \begin{pmatrix} 0 & 0 & 0 \\ 0 & 0 & -1 \\ 0 & 1 & 0 \end{pmatrix} \qquad l_y = \begin{pmatrix} 0 & 0 & 1 \\ 0 & 0 & 0 \\ -1 & 0 & 0 \end{pmatrix} \qquad l_z = \begin{pmatrix} 0 & -1 & 0 \\ 1 & 0 & 0 \\ 0 & 0 & 0 \end{pmatrix} \tag{20}$$

and $L_x := i \cdot l_x, L_y := i \cdot l_y, L_z := i \cdot l_z$. The complex spherical harmonics are eigenvectors of $L_z$ with eigenvalues $-l, ..., l-1, l$.

We will use the operation of $L_z$ on the complex version of $\mathcal{H}^{(l)}$, but once we know that matrix multiplication is related to the Clebsch–Gordan operation by a nonzero factor, we are free to go back to computing in the real vector space spanned by the real spherical harmonics.

On $Lin(\mathcal{H}^{(l_1)}, \mathcal{H}^{(l_2)}) \times Lin(\mathcal{H}^{(l_2)}, \mathcal{H}^{(l_3)})$ we have the concatenation of linear maps (i.e. matrix product), in terms of $\mathcal{H}^{(l_2)} \otimes \mathcal{H}^{(l_1)^*}$ and $\mathcal{H}^{(l_3)} \otimes \mathcal{H}^{(l_2)^*}$ this product is given by

$$(\boldsymbol{v}_3 \otimes \boldsymbol{v}_2^*) \cdot (\boldsymbol{w}_2 \otimes \boldsymbol{w}_1^*) = \langle \boldsymbol{v}_2^*, \boldsymbol{w}_2 \rangle_{alg} \cdot (\boldsymbol{v}_3 \otimes \boldsymbol{w}_1^*)$$

We now choose a (complex) orthonormal basis $\boldsymbol{b}_{-l}, ..., \boldsymbol{b}_{l-1}, \boldsymbol{b}_l$ of $\mathcal{H}^{(l)}$ consisting of eigenvectors of $L_z$, e.g. the complex spherical harmonics. This gives the dual basis $\boldsymbol{b}_m^*$ of $\mathcal{H}^{(l)^*}$ with

$$\langle \boldsymbol{b}_i^*, \boldsymbol{b}_j \rangle_{alg} = \delta_{ij}$$

and a basis $\boldsymbol{b}_{m_1} \otimes \boldsymbol{b}_{m_2}^*$ of $\mathcal{H}^{(l_1)} \otimes \mathcal{H}^{(l_2)^*}$ where $-l_1 \le m_1 \le l1$ and $-l_2 \le m_2 \le l_2$.

The rotation $R_z(\alpha)$ around the $z$–axis with angle $\alpha$ can be given on $\mathcal{H}^{(l)}$ and $\mathcal{H}^{(l)^*}$ by its action on the basis

$$\rho(R_z(\alpha))\boldsymbol{b}_m = e^{i \cdot m \cdot \alpha} \boldsymbol{b}_m, \qquad \rho^*(R_z(\alpha))\boldsymbol{b}_m = e^{-i \cdot m \cdot \alpha} \boldsymbol{b}_m$$

and hence on $\mathcal{H}^{(l_2)} \otimes \mathcal{H}^{(l_1)^*}$ as

$$(\rho_2 \otimes \rho_1^*)(R_z(\alpha))(\boldsymbol{b}_{m_2} \otimes \boldsymbol{b}_{m_1}^*) = e^{i\cdot(m_2-m_1)\cdot\alpha}(\boldsymbol{b}_{m_2} \otimes \boldsymbol{b}_{m_1}^*)$$

For an eigenvector of $L_z$ we call the eigenvalue the *weight* of the eigenvector. So the weight of $b_m$ is $m$, the weight of $b_m^*$ is $-m$, and the weight of $(\boldsymbol{b}_{m_2} \otimes \boldsymbol{b}_{m_1}^*)$ is $m_2 - m_1$.

The matrix multiplication is given on the basis vectors as

$$\begin{aligned}(\boldsymbol{b}_{m_3} \otimes \boldsymbol{b}_{m_2'}^*) \cdot (\boldsymbol{b}_{m_2} \otimes \boldsymbol{b}_{m_1}^*) &= \langle \boldsymbol{b}_{m_2'}^*, \boldsymbol{b}_{m_2} \rangle_{alg} (\boldsymbol{b}_{m_3} \otimes \boldsymbol{b}_{m_1}^*) \\ &= \begin{cases} \boldsymbol{b}_{m_3} \otimes \boldsymbol{b}_{m_1}^* & \text{if } m_2' = m_2 \\ 0 & \text{else} \end{cases}\end{aligned} \tag{21}$$

In particular, the weights add up when we multiply these basis vectors of $Lin(\mathcal{H}^{(l)}, \mathcal{H}^{(l')})$. As matrices with respect to the bases $\boldsymbol{b}_{-l}, ..., \boldsymbol{b}_l$ of $\mathcal{H}^{(l)}$ and $\boldsymbol{b}_{-l'}, ..., \boldsymbol{b}_{l'}$ of $\mathcal{H}^{(l')}$this $\boldsymbol{b}_m \otimes \boldsymbol{b}_{m'}^*$ is the matrix that has a 1 at row $m$ and column $m'$ and is 0 elsewhere, its weight is how far above the diagonal the 1 entry is.
In the following, we will use the abbreviation

$$\boldsymbol{b}_{m,m'} := \boldsymbol{b}_m \otimes \boldsymbol{b}_{m'}^*.$$

for these basis elements of $Lin(\mathcal{H}^{(l)}, \mathcal{H}^{(l')})$.

The irreducible representations $\mathcal{H}^{(a)}$ inside $\mathcal{H}^{(l_2)} \otimes \mathcal{H}^{(l_1)^*}$ have a basis of $L_z$ eigenvectors of weights $-a, ..., a$ and in particular have a highest weight vector $\boldsymbol{v}_a$ (unique up to scalars) which is characterized by $L_+ \boldsymbol{v}_a = 0$ for $L_+ = L_x + iL_y$. Since it is of weight $a$, it must be a linear combination of $\boldsymbol{b}_{m,m'}$ with $m - m' = a$. The action of an element $L \in \mathfrak{so}(3)$ on $\boldsymbol{b}_{m,m'} \in \mathcal{H}^{(l_2)} \otimes \mathcal{H}^{(l_1)^*}$ is given by

$$(\rho_2 \otimes \rho_1^*)(L)(\boldsymbol{b}_m \otimes \boldsymbol{b}_{m'}^*) = (\rho_2(L)\boldsymbol{b}_m) \otimes \boldsymbol{b}_{m'}^* + \boldsymbol{b}_m \otimes (\rho_1^*(L)\boldsymbol{b}_{m'}^*)$$

so in particular

$$\begin{aligned}(\rho_2 \otimes \rho_1^*)(L_+)(\boldsymbol{b}_{m,m'}) &= (\rho_2(L_+)\boldsymbol{b}_m) \otimes \boldsymbol{b}^*_{m'} + \boldsymbol{b}_m \otimes (\rho_1^*(L_+)\boldsymbol{b}^*_{m'}) \\ &= c_1 \cdot \boldsymbol{b}_{m+1,m'} + c_2 \cdot \boldsymbol{b}_{m,m'+1}\end{aligned}$$

for some nonzero constants $c_1, c_2 \in \mathbb{C}$.

As a consequence, the highest weight vector $\boldsymbol{v}_a$ of the copy of $\mathcal{H}^{(a)}$ in $\mathcal{H}^{(l_2)} \otimes \mathcal{H}^{(l_1)^*}$ must be a linear combination of $\boldsymbol{b}_p \otimes \boldsymbol{b}_q$ with $p - q = a$ in which every term has a nonzero coefficient, since at every position $p', q'$ with $p' - q' = a + 1$ the contributions of $p' - 1, q'$ and $p', q' - 1$ have to cancel.

So write for any $a = 0, 1, 2, ..., 2l$ the highest weight vector $\boldsymbol{v}_a$ as

$$\boldsymbol{v}_a = c_{a,l} \cdot \boldsymbol{b}_{l,l-a} + c_{a,l-1} \cdot \boldsymbol{b}_{l-1,l-a-1} + ... + c_{a,a-l} \cdot \boldsymbol{b}_{a-l,-l}$$

with all $c_{a,j} \in \mathbb{C}$ nonzero.

Then for $0 \le a, b, a + b \le 2l$ the matrix product of highest weight vectors of the copies of $\mathcal{H}^{(a)}$ and $\mathcal{H}^{(b)}$ in $\mathcal{H}^{(l_2)} \otimes \mathcal{H}^{(l_1)^*}$ must have the form

$$\boldsymbol{v}_a \cdot \boldsymbol{v}_b = d_{a+b,l} \cdot \boldsymbol{b}_{l,l-a-b} + d_{a+b,l-1} \cdot \boldsymbol{b}_{l-1,l-a-b-1} + ... + d_{a+b,a+b-l} \cdot \boldsymbol{b}_{a+b-l,-l}$$

and from (21) we get

$$\begin{aligned}d_{a+b,l} &= c_{a,l} \cdot c_{b,l} \\ &\dots \\ d_{a+b,a+b-l} &= c_{a,a-l} \cdot c_{b,b-l}\end{aligned}$$

so also all resulting coefficients of the matrix product are nonzero, so in particular the product cannot be 0.

## N.4 Proof of Theorem 5

The previous computation was the main argument needed to prove Theorem 5: We now know that a matrix multiplication computes all Clebsch–Gordan operations (up to non–zero scalar factors). This would be enough to use matrix multiplication for Clebsch–Gordan operations if we always used one matrix for one input, and would extract one output from the result. It remains to show that starting from the matrix moments

$$M_{a,b,l}(\gamma) := \iota_{a,b,l} \sum_{\boldsymbol{r} \in S_\gamma} Y_l(\boldsymbol{r}) \tag{22}$$

and computing matrix products of several factors (without extracting the irreducible components after each matrix multiplication) gives enough covariants to span the vector space of all covariant functions with values in $\mathcal{H}^{(l)}$ for all $l$.

However, for this we just need to start with a vector and keep multiplying matrices from the left, the result is again a vector, so there is nothing to extract. To make sure this works, we write it down in detail:

We prove this by induction on the number $d$. For $d = 1$ there is nothing to prove: The $M_{0,l,l}(\gamma)$ are the fundamental features. So assume we have $d > 1$ and we already get all concatenation of Clebsch–Gordan operations for $d - 1$.
Assuming the previous step of the Clebsch–Gordan operations resulted in a vector in $\mathcal{H}^{(a)}$, and now we need to simulate the operation $\mathcal{H}^{(l)} \otimes \mathcal{H}^{(a)} \to \mathcal{H}^{(b)}$ for some $|a - l| \leq b \leq a + l$. These inequalities are equivalent to $|a - b| \leq l \leq a + b$ (both are ways to express that $a, b, l$ can appear as sides of a triangle), so we can use $M_{a,b,l}$ and multiply this matrix with the previous result vector in $\mathcal{H}^{(a)}$ to obtain the result vector in $\mathcal{H}^{(b)}$.

As in Theorem 3, all results are either even or odd covariants, so to get $O(3)$–covariants, we only need to include those with the right parity in the linear combination.

# O JAX implementation of matrix multiplication

We implemented the Clebsch–Gordan operation

$$(\mathcal{H}^{(0)} \oplus ... \oplus \mathcal{H}^{(L)}) \otimes (\mathcal{H}^{(0)} \oplus ... \oplus \mathcal{H}^{(L)}) \to \mathcal{H}^{(0)} \oplus ... \oplus \mathcal{H}^{(L)}$$

in the obvious way as a general product in an $(2L+1)^2$–dimensional $\mathbb{R}$–algebra with the Clebsch–Gordan coefficients as multiplication table, and by changing the multiplication table to the one corresponding table for the $(2L+1)^2$–dimensional algebra of $(2L+1) \times (2L+1)$–matrices gives an alternative implementation of matrix multiplication. It turned out that this more complicated way of formulating matrix multiplication is actually significantly faster for small $L$ on TPUs and GPUs! Of course, once $L$ is large enough, the benefit of the hardware support kicks in.

An example computation is:

**Computation A** (Multiplication in arbitrary algebra of dimension 9):

```
Inputs: jnp.arrays of size
input1:  [20480,9,8]
input2:  [20480,9,8]
multTable: [9,9,9,8]
Compute: jnp.einsum('clf,cmf,lmnf->cnf',
                    input1, input2, multTable)
Output:  [20480,9,8]
```

**Computation B** (Multiplication for the special case of $3 \times 3$–matrices):

```
Inputs: jnp.arrays of size
input1:  [20480,3,3,8]
input2:  [20480,3,3,8]
```

```
Compute: jnp.einsum('clmf,cmnf->clnf',
                    input1, input2)
Output:  [20480,9,8]
```

The first two inputs are the same (except treating $3 \times 3$ as 9), computation B is a matrix multiplication of $3 \times 3$ matrices. Computation A does something more general, which needs an extra input of coefficients (for a particular choice of 0's and 1's you get back matrix multiplication). The time used for these operations measured on different platforms was:

Table 2: Time in ms used for computations A / B

| | CPU | TPU | P100 |
|---|---|---|---|
| A | 15 | 0.4 | 0.8 |
| B | 7 | 4.6 | 1.5 |

As expected, the matrix multiplication is faster on CPU, as it does not need to multiply with the entries of the multiplication table. However, on accelerators the more general / complicated computation was faster! (Using `jax.lax.batch_matmul` for computation B gave the same result.) On TPUs, this effect persisted up to $L = 4$, i.e. $9 \times 9$–matrices, for $L \geq 5$ and above the special case matrix multiplication was faster.

# P Details for experiments

## P.1 Distinguishing configurations

We first want to demonstrate in concrete examples that the invariants of Theorem 5 indeed can distinguish configurations in challenging pairs that cannot be distinguished by invariants of low body orders, see Figure 1, part D.

The examples are point sets on the unit sphere $\mathbb{S}^2$. The 2–body invariants would only measure the distance from the center, so in this case the only such invariant is the number of points. In the cases of Figure 1, part D, this would only distinguish between the 3 pairs of 2,

4, 7 points, but not the two configurations of each pair. The corresponding matrix invariant would be $\sum_{\boldsymbol{r}\in S} M_{0,0,0} = \sum_{\boldsymbol{r}\in S} 1 = |S|$.

The first pair can be distinguished by a 3–body invariant, corresponding to a product of two fundamental features: The fundamental feature is just $\boldsymbol{r} \mapsto r$, and the (only) invariant polynomial (up to scalar multiples) in the fundamental features is

$$\left|\sum_{\boldsymbol{r}} \boldsymbol{r}\right|^2 = \left(\sum x\right)^2 + \left(\sum y\right)^2 + \left(\sum z\right)^2$$

For the configuration 1a the $\sum_{\boldsymbol{r}} \boldsymbol{r}$ is zero, but for the configuration 1b the sum $\sum_{\boldsymbol{r}} \boldsymbol{r}$ gives $(1,1,0)^T$, which has square norm 2.
To express this as matrix product, we write the $L = 1$ fundamental feature as $3 \times 1$ and $1 \times 3$–matrices, i.e. use a=1, b=0 and a=0, b=1 to obtain

$$M_{1,0,1} \cdot M_{0,1,1} = (1,1,0) \cdot \begin{pmatrix} 1 \\ 1 \\ 0 \end{pmatrix} = 2.$$

For the second pair (which needs 4–body invariants), we need a product of 3 matrices, and we use

$$M_{1,0,1} \cdot M_{1,1,2} \cdot M_{0,1,1}$$

where $M_{1,1,2}$ writes the $L = 2$ fundamental feature as a symmetric, traceless $3 \times 3$–matrix.

For the third pair, we define an invariant as a product of 4 matrices which give a linear map

$$\mathbb{R} \xrightarrow{M_{0,2,2}} \mathbb{R}^5 \xrightarrow{M_{2,1,1}} \mathbb{R}^3 \xrightarrow{M_{1,1,2}} \mathbb{R}^3 \xrightarrow{M_{1,0,1}} \mathbb{R}$$

which is then of course again interpreted as a scalar.

## P.2 Synthetic data experiment

For the synthetic experiment we created an invariant PPSD on point sets on $\mathbb{S}^2$ colored with 5 colors. Using the moment tensors

$$T_{i_1 i_2 \dots i_k}(\gamma) \coloneqq \sum_{\boldsymbol{r} \in S_\gamma} \boldsymbol{r}^{\otimes k}$$

it can be expressed as a tensor contraction

$$T_{abcdefghij}(\gamma_1) T_{akn}(\gamma_2) T_{bckl}(\gamma_3) T_{deflm}(\gamma_4) T_{ghijmn}(\gamma_5)$$

(using Einstein summation convention, i.e. summation over all indices is implied, but we write all indices as lower indices). Since tensor contractions are $O(3)$–covariant, this gives an $O(3)$–invariant function on point configurations colored with 5 colors. Each of the five tensors can be computed as a fundamental feature, so the degree of this invariant is 5 (i.e. its body order 6). The first tensor is of order 10, so it contains irreducible representations up to $L = 10$.

We generated 8192 training configurations, and 4096 test configurations of 20 points uniformly randomly sampled on the $\mathbb{S}^2$, with 4 points assigned to each of the 5 colors.

For the **Clebsch–Gordan** nets we use maximal degree $L = 10$ and 25 channels. First we use the method `e3x.so3.irreps.spherical_harmonics` to compute the spherical harmonics up to $L = 10$ and then combine them linearly (with learnable weights) to 25 combinations for each of 5 "factors". Then we use the the method `e3x.so3.clebsch_gordan` from E3x (see[6]), to "multiply" these layers $1 - 4$. This means in particular that the data in each layer are 25 vectors in

$$\mathcal{H}^{(0)} \oplus \dots \oplus \mathcal{H}^{(10)}$$

and each Clebsch–Gordan operation takes $11^3 \cdot 25 = 33275$ learnable parameters.

For the last "multiplication" we use a scalar product (a performance optimization, since we

would only use the $\mathcal{H}^{(0)}$–part of the Clebsch–Gordan product), this then only uses $11 \cdot 25 \cdot 25 = 6875$ learnable parameters to compute all scalar products, which are then added up to give the scalar result.

For the **matrix multiplication** nets we used big matrices with total side length

$$4 \times (2 \cdot \mathbf{4} + 1) + 4 \times (2 \cdot \mathbf{5} + 1) = 80,$$

i.e. 4 copies of $L = 4$ and 4 copies of $L = 5$. Since the $(2 \cdot 5 + 1) \times (2 \cdot 5 + 1)$–matrices correspond to the representation

$$\mathcal{H}^{(5)} \otimes \mathcal{H}^{(5)} = \mathcal{H}^{(0)} \oplus \ldots \oplus \mathcal{H}^{(10)},$$

this corresponds again to a maximal $L = 10$ being used, matching the Clebsch–Gordan setting. The number of matrices is 64, which is a bit larger than the 25 channels used in the Clebsch–Gordan setting, but on the other hand this only contains 16 matrices of the full size containing a $\mathcal{H}^{(10)}$ component, a bit less than the 25 in the Clebsch–Gordan case. (We cannot give fully equivalent settings if we want to use also non–square constituent matrices, but this seems to be a reasonable approximation which also gives comparable accuracies.)

As in the Clebsch–Gordan case, we start with computing the spherical harmonics and combine their sums into vectors in $\mathcal{H}^{(0)}, \ldots, \mathcal{H}^{(10)}$; again we use 5 factors (i.e. matrices in this case). After computing their matrix product, we take the traces of the $2 \cdot (4 \times 4) = 32$ square sub–matrices that occur in the big matrix, and combine them in a linear combination (with learnable coefficients), this is the direct equivalent of combining the $\mathcal{H}^{(0)}$–components as in the Clebsch–Gordan case (although without the performance optimization).

We trained with stochastic gradient descent for 40 episodes on the training set (with learning rate and batch size tuned separately for the two cases), and evaluated on the test set. Averaging over 10 runs gave the learning curves reported in the main part.

As is to be expected, more / larger matrices usually give lower error, but longer execution

time. Below we add as an example a network with matrices of size

$$4 \times (2 \cdot \mathbf{1} + 1) + 4 \times (2 \cdot \mathbf{2} + 1) + 4 \times (2 \cdot \mathbf{4} + 1) + 4 \times (2 \cdot \mathbf{6} + 1) = 120$$

In the resulting learning curves (averaged over 10 runs)

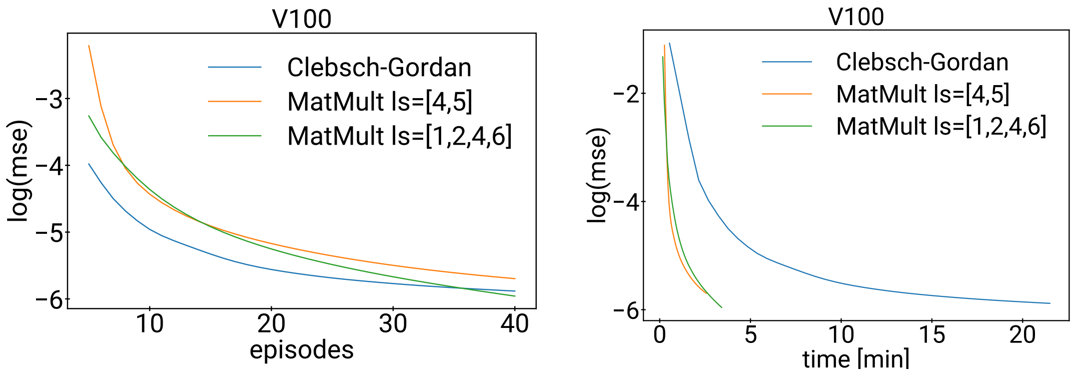


we can see on the left hand side that the accuracy using these Matrix Multiplication nets for the same number of episodes is similar to the Clebsch–Gordan nets, but on the right hand side we can see that the Matrix Multiplication nets obtain this accuracy much faster.

## P.3 Experiment on atomistic simulation

In this experiment we want to show that we can use this method to obtain interesting results on real world data. We use the data set MD-22 published with ([20]), and compare the results with the accuracies reported there and in ([21]).

While the previous experiment on a toy data set tried to use the "same" architecture for both a Clebsch–Gordan version and a Matrix Multiplication version and observed the speed difference, this chemistry experiment does not focus on speed, but on the simplicity of the architecture: We only learn a linear combination of polynomial features (strictly speaking, for the radial part we use polynomials of the *log* radius [and a cutoff], as this seems to be more aligned to the physically meaningful functions of the radius). However, we will see that our algorithm allows using a large number of these polynomial features, so in the end this

simple local algorithm can still compete with more complicated ones developed specifically for such chemistry problems. One motivation for a simple architecture would be to directly study the benefit of higher body orders and higher order features in the simplest possible architecture to obtain insights into the nature of the energy function that is learned.

The MD-22 data set / benchmark highlights one specific task – learning the potential energy surface / force field for one large molecule from a limited set of conformations of this molecule. This means in particular that the learned force field does not have to generalize to a wide range of other molecules (which may be an advantage for "smaller" models like So3krates), but on the other hand it has to learn effects in larger molecules (which may be an advantage for "global" models like sGDML). So this particular experiment is just a first sanity check for the use of our features for atomistic simulation in the simplest architecture. A more realistic evaluation of our features on different data sets will use our features in different architectures, implementation choices and training procedures to tune the architectures for speed or accuracy or broad applicability on small or large training sets of small or large molecules; such a practical evaluation of different architectures is beyond the scope of the present paper establishing the theoretical background of the features.

In this experiment with the Matrix Multiplication features on the MD-22 data set, we use the architecture specified in Appendix E to learn "energy contributions" for each atom in a molecule, these contributions are summed over all atoms to give the energy of the configuration. To obtain the force acting on one atom, the derivative of the energy in direction of the position of this atom is computed. This is done by automatic differentiation in JAX. The network is trained mainly on the forces: We use a mean square error as the loss function on both forces and energies, but with a scaling that puts almost all the weight on the forces.

Since these energy contributions should depend on the element type of each atom, we ideally would learn different functions for each atom type. However, as a more parsimonious solution

we just add learnable additive constants to all vectors and sub–matrices which depend on the element.

In contrast to the previous synthetic data experiment, the points are now in $\mathbb{R}^3$, and we use as radial functions Chebyshev polynomials of the log radius and a cutoff radius of 6 Å(so we are effectively in our case 2ii). This is implemented in E3x as `e3x.nn.functions.chebyshev.exponential_chebyshev`.

It seems to be beneficial to start with a smaller number of radial basis functions and increase their number during the training, this increases the expressivity of the radial basis functions only gradually and makes the end result a bit smoother. We start with the first 12 Chebyshev polynomials as radial basis functions, and add a new radial basis function every 4 epochs. Similarly, we start with products of $b = 4$ matrices and increase every 5 epochs by one, up to a maximal $b = 12$.

The sub–matrices have side lengths 1,3,...,11, corresponding to $L = 0, 1, ..., 5$, so the maximal $L$ occurring in the sub–matrices is $L = 10$. We use each of these lengths 3 times, so the big matrix has a side length of

$$3 \cdot \Big( (2 \cdot 0 + 1) + (2 \cdot 1 + 1) + ... + (2 \cdot 5 + 1) \Big) = 108$$

We use $n_{mat} = 8$ of such matrix products, and we use $n_{vec} = 12$ vectors on each end of the product, but in slight deviation to the original algorithm as specified in Appendix E we do not reuse the vectors, so we "only" get

$$n_{mat} \cdot r \cdot n_{vec} = 8 \cdot (6 \cdot 3) \cdot 12 = 1728$$

invariants from the full products of 12 matrices. We also do the same for each partial product of 1,2,...,11 matrices that we obtain while computing the full products, so in total we get $12 \cdot 1728 = 20736$ invariants, of which we then learn the best linear combination.

For the two largest molecules we use slightly smaller networks, as specified in Table 3.

Table 3: Size of the network

| | small | buckyball catcher | nanotubes |
|---|---|---|---|
| $n_{mat}$ | 8 | 5 | 3 |
| $n_{vec}$ | 12 | 10 | 10 |
| $L_{max}$ | 10 | 10 | 8 |
| # repeats $L$ | 3 | 3 | 4 |
| # invariants | 20,736 | 10,800 | 7,200 |

Maybe surprisingly, these simple networks using only a linear combination of the invariants, no message passing, no nonlinearities (apart from the matrix multiplication), give results that are similar to those of the more sophisticated methods, see Table I in the main part.

# References


(1) Matsumura, H. *Commutative algebra, second edition*; Benjamin/Cummings Publishing Company, Reading, Massachusetts, 1980.

(2) Pozdnyakov, S. N.; Willatt, M. J.; Bartók, A. P.; Ortner, C.; Csányi, G.; Ceriotti, M. Incompleteness of atomic structure representations. *Physical Review Letters* **2020**, *125*, 166001.

(3) Shapeev, A. V. Moment tensor potentials: A class of systematically improvable interatomic potentials. *Multiscale Modeling & Simulation* **2016**, *14*, 1153–1173.

(4) He, K.; Zhang, X.; Ren, S.; Sun, J. Deep residual learning for image recognition. *2016 IEEE Conference on Computer Vision and Pattern Recognition (CVPR)* **2015**, 770–778.

(5) Pozdnyakov, S. N.; Willatt, M. J.; Bartók, A. P.; Ortner, C.; Csányi, G.; Ceriotti, M. Incompleteness of atomic structure representations. *Physical Review Letters* **2020**, *125*, 166001.

(6) Unke, O. T.; Maennel, H. E3x: $E(3)$-equivariant deep learning made easy. arXiv, 2022; `https://doi.org/10.48550/arXiv.2401.07595`.

(7) Guillemin, V.; Pollack, A. *Differential topology*; AMS Chelsea Publishing, Providence, Rhode Island, 2010.

(8) Clarkson, K. L. Tighter bounds for random projections of manifolds. Proc. 24th Annual Symp. on Computational Geometry. 2008; pp 39–48.

(9) Baraniuk, R. G.; Wakin, M. B. Random projections of smooth manifolds. *Foundations of Computational Mathematics* **2009**, *9*, 5177.

(10) Mukai, S. *An Introduction to Invariants and Moduli*; Cambridge Studies in Advanced Mathematics; Cambridge University Press, 2003.

(11) Pflaum, M. *Analytic and geometric study of stratified spaces: Contributions to analytic and geometric aspects*; Analytic and Geometric Study of Stratified Spaces; Springer, Berlin, 2001.

(12) Goresky, M.; MacPherson, R. *Stratified Morse theory*; Springer Berlin Heidelberg, 1988.

(13) Kamke, T.; Kemper, G. Algorithmic invariant theory of nonreductive groups. *Qualitative Theory of Dynamical Systems* **2012**, *11*, 79–110.

(14) Bochnak, J.; Coste, M.; Roy, M. *Real algebraic geometry*; Springer Berlin Heidelberg, 2013.

(15) Shiota, M. *Geometry of subanalytic and semialgebraic sets*; Birkhäuser, Boston, 1997.

(16) Valette, G. On subanalytic geometry. `http://www2.im.uj.edu.pl/gkw/sub.pdf`, (accessed 2024-10-18), 2023.

(17) Kosmann-Schwarzbach, Y.; Singer, S. *Groups and Symmetries: From Finite Groups to Lie Groups*; Universitext; Springer New York, 2009.

(18) Procesi, C. *Lie Groups: An Approach through Invariants and Representations*; Springer New York, 2006.

(19) Neusel, M.; Smith, L. *Invariant theory of finite groups*; American Mathematical Society, Providence, Rhode Island, 2002.

(20) Chmiela, S.; Vassilev-Galindo, V.; Unke, O. T.; Kabylda, A.; Sauceda, H. E.; Tkatchenko, A.; Müller, K.-R. Accurate global machine learning force fields for molecules with hundreds of atoms. *Science Advances* **2023**, *9*.

(21) Frank, T.; Unke, O.; Müller, K.-R. So3krates: Equivariant attention for interactions on arbitrary length-scales in molecular systems. Advances in Neural Information Processing Systems. 2022; pp 29400–29413.
